# Targeted HDAC8 inhibition with non-hydroxamate [1,2,4]triazolo[4,3-a] quinoline compounds

**DOI:** 10.1038/s41598-026-38490-y

**Published:** 2026-02-20

**Authors:** N. V. M. Rao Bandaru, Ashna Fathima, Suryansh Sengar, Markus Schweipert, Kosana Sai Chaitanya, Muzaffar-Ur-Rehman Mohammed, Suraj T. Gore, Trinath Jamma, Vivek Sharma, Chandrasekhar Abbineni, Franz-Josef Meyer-Almes, Kondapalli Venkata Gowri Chandra Sekhar

**Affiliations:** 1 Department of Chemistry, Birla Institute of Technology and Science, Pilani, Hyderabad Campus, Jawahar Nagar, Hyderabad, Telangana 500 078 India; 2 Department of Biological Sciences, Birla Institute of Technology and Science, Pilani, Hyderabad Campus, Jawahar Nagar, Hyderabad, Telangana 500 078 India; 3 Aurigene Oncology Limited, 39-40 KIADB Industrial Area Electronic City Phase II, Hosur Road, Bangalore, 560 100 India; 4 Department of Chemical Engineering and Biotechnology, University of Applied Sciences Darmstadt, Haardtring 100, 64295 Darmstadt, Germany; 5 European University of Technology, European Union, Darmstadt, Germany; 6 Department of Pharmacy, Birla Institute of Technology and Science, Pilani Campus, Pilani, Rajasthan 333031 India

**Keywords:** Histone deacetylase, 1,2,4-Triazolo[4,3-a] quinoline, Inhibitors, α-Amino amides hydroxamate, Biochemistry, Enzymes, Isoenzymes, Drug development

## Abstract

Histone deacetylase 8 (HDAC8) is a key enzyme involved in regulating gene expression and tumor development, positioning it as an attractive target for neuroblastoma. In this work, we designed and synthesized a novel series of substituted [1,2,4]Triazolo[4,3-a]quinoline derivatives to investigate their potential as HDAC8 inhibitors. Structural insights into their inhibitory activity were gained through molecular docking studies, highlighting critical interactions within the HDAC8 active site. To assess the stability of these interactions, molecular dynamics simulations were performed, confirming that the compounds maintained strong and stable binding within the HDAC8 enzyme. The most promising inhibitors **9h** and **9m** demonstrated significant efficacy in IMR-32 neuroblastoma cells, but had much weaker effects on the HCT116 and MCF7 cancer cell lines, as well as on the normal control cell line, HEK293. Further biological evaluations, including colony formation and cell migration assays, revealed their potential to inhibit the growth and spread of neuroblastoma tumor cells. Additional studies on cell cycle progression, apoptosis induction, and SMC3 acetylation indicated increased acetylation levels without altering total SMC3 protein levels, suggesting effective HDAC8 target engagement. In summary, comprehensive molecular modelling and biological assessments have demonstrated the strong potential of these non-hydroxamate-based HDAC8 inhibitors for treating neuroblastoma.

**Supplementary Information:**

The online version contains supplementary material available at 10.1038/s41598-026-38490-y.

## Introduction

Acetylation and deacetylation of lysine residues on histones and other proteins play a crucial role in post-translational modifications and modulation of cellular processes at multiple levels^1–4^. Histone acetylase (HAT) enzymes are responsible for acetylation of lysine residues on histone proteins, and Histone deacetylase (HDAC) enzymes are accountable for deacetylation of lysine residues on histone and non-histone proteins^5–7^. HDACs are classified into four subfamilies: class I ((HDAC 1,2,3 and 8), class II (HDAC 4, 5, 6, 7, 9, and 10), class III (Sirt 1 to Sirt 7), and class IV (HDAC 11). Class I, II, and IV are Zn^2+^dependent enzymes, whereas class III are NAD^+^ dependent enzymes^8–10^. HDACs are established as attractive targets for many diseases, including neurodegenerative disorders, cancer, and inflammatory diseases^11–16^. So far, FDA has approved six pan HDAC inhibitors for T-cell lymphoma and multiple myeloma^17–19^. However, isoform-selective HDAC inhibitors have attained great interest due to the poor therapeutic index of pan-HDAC inhibitors^20^. Notably, HDAC8 attains attention owing to its unique features in the binding site of the pocket and its prominent role in specific cancers and neurodegenerative diseases^21–23^. HDAC8 belongs to class I zinc-dependent enzyme, which shuttle between cytoplasm and nucleus^24,25^. HDAC8 has many interaction partner proteins such as p53, AT-rich interactive domain-containing protein 1A (ARID1a), estrogen-related receptor α (ERRα), and the structural maintenance of chromosome three protein (SMC3)^26–28^. Besides its enzymatic activity, the scaffolding function of HDAC8 is also crucial for cellular signaling processes^29–31^. Aberrant expression of HDAC8 shows a notable correlation with many cancers, such as T-cell lymphoma, colon cancer, breast cancer, lung cancer, acute myeloid leukemia, hepatocellular carcinoma (HCC), and childhood neuroblastoma^32–37^. In the current study, we show the design and synthesis of a novel class of non-hydroxamate-based HDAC8 inhibitors. These HDAC8 inhibitors intercept cancer cell survival in vitro, suggesting a promising role for these molecules as anticancer agents.

**Fig. 1:**
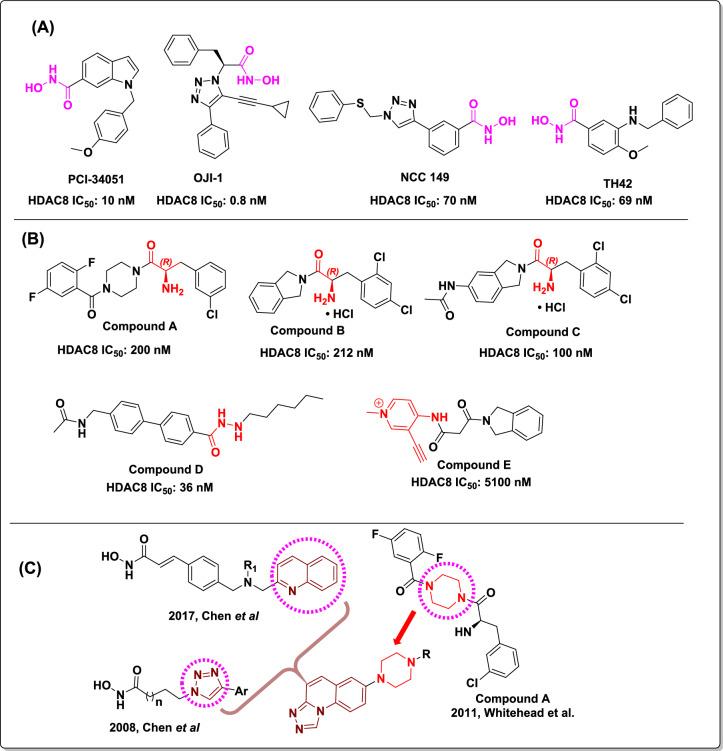
(**A**) Reported hydroxamate-based HDAC8 inhibitors. (**B**) Reported non-hydroxamate-based HDAC8 inhibitors. (**C**) Design strategy for novel triazole quinoline as cap group in our series.

Pharmacophores of reported HDAC inhibitors have three main components: A zinc-binding group (ZBG) that chelates with the zinc ion in the active site of the protein, a cap group that interacts with residues on the protein’s outer surface, and a linker that connects the cap group and ZBG^19^. Several HDAC8 inhibitors have been reported with the hydroxamate group as the zinc chelating group (representative examples in Fig. 1A). PCI-34051, a well-known selective HDAC8 inhibitor reported in 2008 by Balasubramanian et al.,^38^ shows the phenotypic response in cell lines derived from T-cell lymphomas or leukemia. In 2012, using click chemistry, Suzuki et al. reported that a triazole compound NCC 149 was a selective HDAC8 inhibitor^39^. In 2016, Ingham et al. identified OJI-1 as a selective HDAC8 inhibitor by repurposing the existing library compounds to Zn (II) Chelating moieties^40^. In 2017, Heimburg et al. identified TH42 as a selective HDAC8 inhibitor using a structure-based design plan^41^. The higher affinity of hydroximic acids to chelate with metal confers limitations to their efficacy and also leads to toxicity, which restricts the scope of current hydroxamate-based inhibitors^42^. Hence, zinc chelating groups other than hydroximic acid attract the medicinal chemist’s attention to inhibit HDAC8 activity and to mitigate the side effects^43,44^. Some of the reported non-hydroxamate HDAC8 inhibitors were reported to attain more selectivity towards HDAC8 (representative examples in Fig. 1B). In 2011, Whitehead et al. reported α-amino amide derivatives with an alternative Zinc chelating group. These inhibitors occupy the acetate release channel of HDAC8, which offers selectivity towards HDAC8 protein compared to other isoforms^45^. In their efforts, compound **A** and the free base of compound **B** showed good potency and selectivity towards HDAC8. SAR exploration of these α-amino amides was carried out by Greenwood et al. in 2020. This group used compound B as a reference compound and explored the SAR of α-amino amide derivatives, including SAR around the isoi.7 fdoline ring. They have also explored the cap group modifications, such as tetrahydroisoquinoline, and used the flexible linkers. However, the isoindoline ring only retained the HDAC8 potency, and 5-substituted isoindoline derivative compound **C** showed good potency^46^. In addition to these efforts, in 2022, the Ping Sun group reported alkyl hydrazide as an HDAC8 competitive inhibitor and compound **D** showed effective T-cell modulation^47^. In 2023, our group reported a novel allosteric covalent HDAC8 inhibitor by using electrophile fragment screening and connecting the intial fragment-hit compound to the known isoindoline cap group with an appropriate linker to find compound **E**, which showed selectivity towards HDAC4 and HDAC8^48^.

## Rational drug design

Traditional inhibitors of HDAC8, which consist of hydroxamic acid as a zinc-chelating group, have limited therapeutic potential due to their inferior selectivity over other HDAC paralogues, as well as associations with genotoxicity and mutagenicity^43,44^. We were mainly attracted to the zinc chelating group reported by Whitehead et al. and Greenwood et al., owing to its distinct binding mode and availability of crystal structure with these probes; these α-amino amide probes not only exhibit bidentate interaction with Zinc but also extend their binding into a deeper acetate release channel. These features offer a selectivity handle for targeting HDAC8^45,46^. To the best of our knowledge, though these probes showed biochemical activity towards HDAC8, phenotypic response and mechanistic engagement in a cellular context were not established. Potent compounds from both groups have the standard feature of isoindoline as a cap group or interacting partner of the outer surface area of HDAC8. We aimed to explore the cap groups to establish the phenotypic response of these compounds. Towards this, we designed a novel triazolo-quinoline cap group using structure-guided scaffold hopping strategies derived from well-known HDAC cap groups, as illustrated in Fig. 1C.

**Fig. 2:**
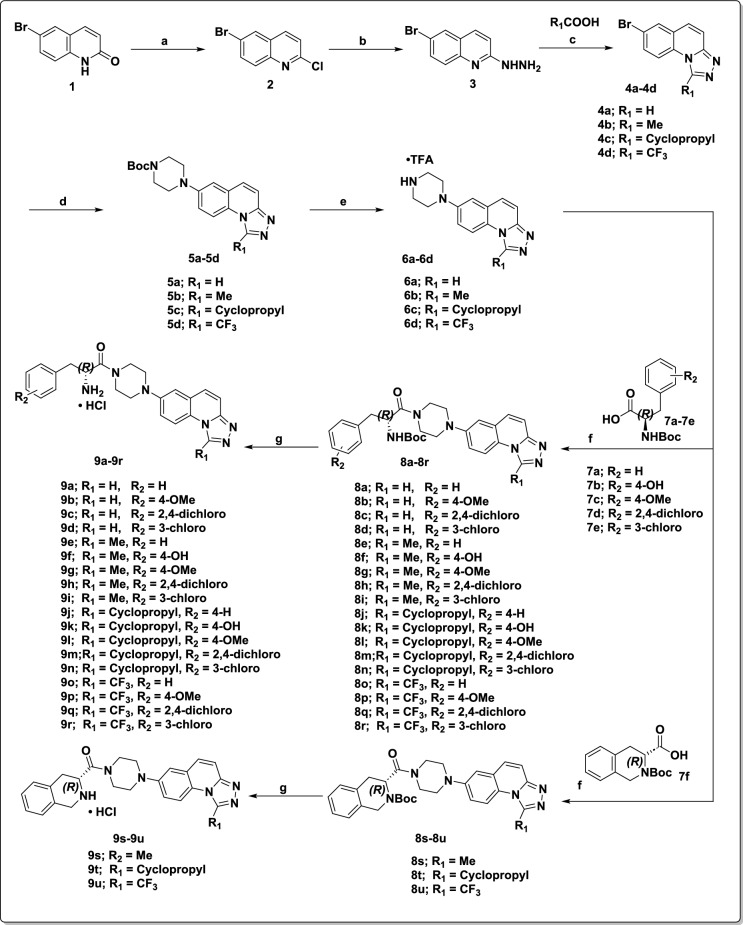
The synthetic route for compounds 9a to 9u. Reagents and conditions: (**a**) POCl_3_, 110 °C, 16 h; (**b**) NH_2_NH_2_·H_2_O, 100 °C, 16 h; (**c**) R_1_COOH, 120 °C, 16 h; (**d**) *tert*-butyl piperazine-1-carboxylate, Pd_2_dba_3_, Xantphos, Cs_2_CO_3_, 1,4-dioxane, 110 °C, 16 h; (**e**) TFA, CH_2_Cl_2_, RT, 4 h; (**f**) corresponding carboxylic acid, HATU, DIPEA, DMF, RT, 2 h; (**g**) 4N HCl in 1,4-dioxane, RT, 2 h.

We have selected quinoline moiety to maintain hydrophobic interactions and the triazole group to mimic the N-acetyl group in compound C. Then we selected piperazine as the linker group from compound **A** and connected it to the 7^th^ position of triazolo quinoline to maintain the linearity^45,49–52^. In this study, we reported a series of twenty-one compounds **9a** to **9u** having the α-amino amide as Zn chelating group, triazolo quinoline as the cap group, and piperazine as a linker. Compounds **9a** to **9u** were synthesized as represented in Fig. 2**.**

We systematically varied four substituents (R_1_ = H, Me, Cyclopropyl, and trifluoro methyl) on the triazole ring of the triazole quinoline cap group. Additionally, we explored various substituents on the phenyl group of phenylalanine (**9a** to **9r**) as well as synthesized constrained phenylalanine analogues (**9s** to **9u**). We maintained the chirality as R configuration for all the amino acids, as reported by Whitehead group^45^.

## Results and discussion

### Synthesis of designed compounds

Synthesis of 1,2,4-triazolo[4,3-a]quinoline derivatives began with the treatment of 6-bromoquinoline **1** with POCl_3_ followed by hydrazine hydrate to afford the 6-bromo-2-hydrazinylquinoline as **intermediate 3**. This **intermediate 3** was then treated with corresponding carboxylic acids to afford the 1,2,4-triazolo[4,3-a] quinoline derivatives **4a** to **4d**^53^. The resultant intermediate **4a** to **4d** were treated with Boc-piperazine under Buchwald conditions, followed by deprotection of -Boc using TFA to afford the 7-(piperazin-1-yl) triazolo quinoline intermediates **6a** to **6b**. These intermediates were treated with corresponding Boc protected α amino acids to afford the intermediates **8a** to **8u,** which were subjected to deprotection of -Boc using 4 M HCl in 1,4-dioxane to afford the corresponding final compounds **9a** to **9u** as hydrochloride salts. All the synthesized derivatives are characterized for their structural confirmation and the ^1^H, ^13^C NMR, HPLC and HRMS spectras are provided in the supporting information file (Figs. S1–S84).

### HDAC8 inhibition and iso-enzyme selectivity

All twenty-one compounds were screened for HDAC8 inhibitory activity; the results are listed in Table 1. Among these, seven compounds (**9r, 9m, 9d, 9h, 9c, 9n, and 9i**) featuring 3-chloro and 2,4-dichloro substituents showed significant HDAC8 inhibitory activity. The corresponding dose–response curves are presented in the supporting information (Figs. S85 and S86). We further evaluated the selectivity of most active compounds (**9r**, **9m**, **9d** and **9h**) against HDAC1 as a representative of class I HDAC enzymes, HDAC4, a member of class IIa, HDAC6 and HDAC10 from class IIb and HDAC11, the only member of class IV. None of the tested compounds were active against the HDAC panel, except for HDAC8, demonstrating the selectivity of these compounds (Table 2). Compounds **9r** and **9m** notably demonstrated comparable HDAC8 inhibitory activity ≤ 0.4 µM comparable to reference compound **B**. Additionally, SAR analysis revealed that compounds bearing 3-chloro and 2,4-dichloro substituents on the phenyl ring of α amino acids are more potent compared to other derivatives in the series. This trend is consistent with findings from the Novartis group, which reported that chloro-substituted compounds enhance HDAC8 selectivity and potency by providing additional interactions with the side-chain amino acids in the acetate release channel^45^. These findings suggest that our compounds also potentially fit into the deep acetate release pocket of the HDAC8.

**Table 1:** HDAC8 inhibitory activity of compounds **9a** to **9u**


Entry	R_1_	R_2_	HDAC8 *IC_50_ (µM)
**9a**	H	H	27 ± 3
**9b**	H	4-OMe	> 35
**9c**	H	2,4-Dichloro	1.1 ± 0.2
**9d**	H	3-Chloro	0.72 ± 0.01
**9e**	Me	H	8.3 ± 0.8
**9f**	Me	4-OH	> 35
**9g**	Me	4-OMe	> 35
**9h**	Me	2,4-Dichloro	0.80 ± 0.20
**9i**	Me	3-Chloro	1.9 ± 0.1
**9j**	Cyclopropyl	H	> 35
**9k**	Cyclopropyl	4-OH	17 ± 2
**9l**	Cyclopropyl	4-OMe	9 ± 1
**9m**	Cyclopropyl	2,4-Dichloro	0.40 ± 0.20
**9n**	Cyclopropyl	3-Chloro	11 ± 0.1
**9o**	CF_3_	H	> 35
**9p**	CF_3_	4-OMe	> 35
**9q**	CF_3_	2,4-Dichloro	3 ± 1
**9r**	CF_3_	3-Chloro	0.30 ± 0.20
**9s**	Me	H	> 35
**9t**	Cyclopropyl	H	> 35
**9u**	CF_3_	H	> 35
**Cpd B**			0.20 ± 0.03

*IC_50_ values are presented as the mean ± SD. The assay was performed in triplicate. IC_50_ graphs are provided in the SI section.

**Table 2:** Isoenzyme-selectivity of the most potent HDAC8 inhibitors.

ID	*IC_50_ (µM)								
	HDAC1	HDAC2	HDAC3	HDAC4	HDAC6	HDAC7	HDAC8	HDAC10	HDAC11 11
**9d**	> 35	> 35	18 ± 1	> 35	> 35	> 35	0.72 ± 0.10	> 35	> 35
**9h**	> 35	> 35	1.1 ± 0.1	> 35	> 35	> 35	0.80 ± 0.20	> 35	> 35
**9m**	27 ± 3	34 ± 4	0.73 ± 0.03	> 35	> 35	> 35	0.40 ± 0.20	> 35	> 35
**9r**	> 35	> 35	15 ± 1	> 35	> 35	> 35	0.30 ± 0.20	> 35	> 35
**Cpd B**	1.7^**#**^	–	–	> 50	> 30^**#**^	–	0.20 ± 0.03	–	–

*IC_50_ values are presented as the mean ± SD. The assay was performed in triplicate, ^**#**^ reported values^45^ for compound **B**. IC_50_ graphs are provided in the SI section.

### Molecular docking and free-energy calculations

**Fig. 3:**
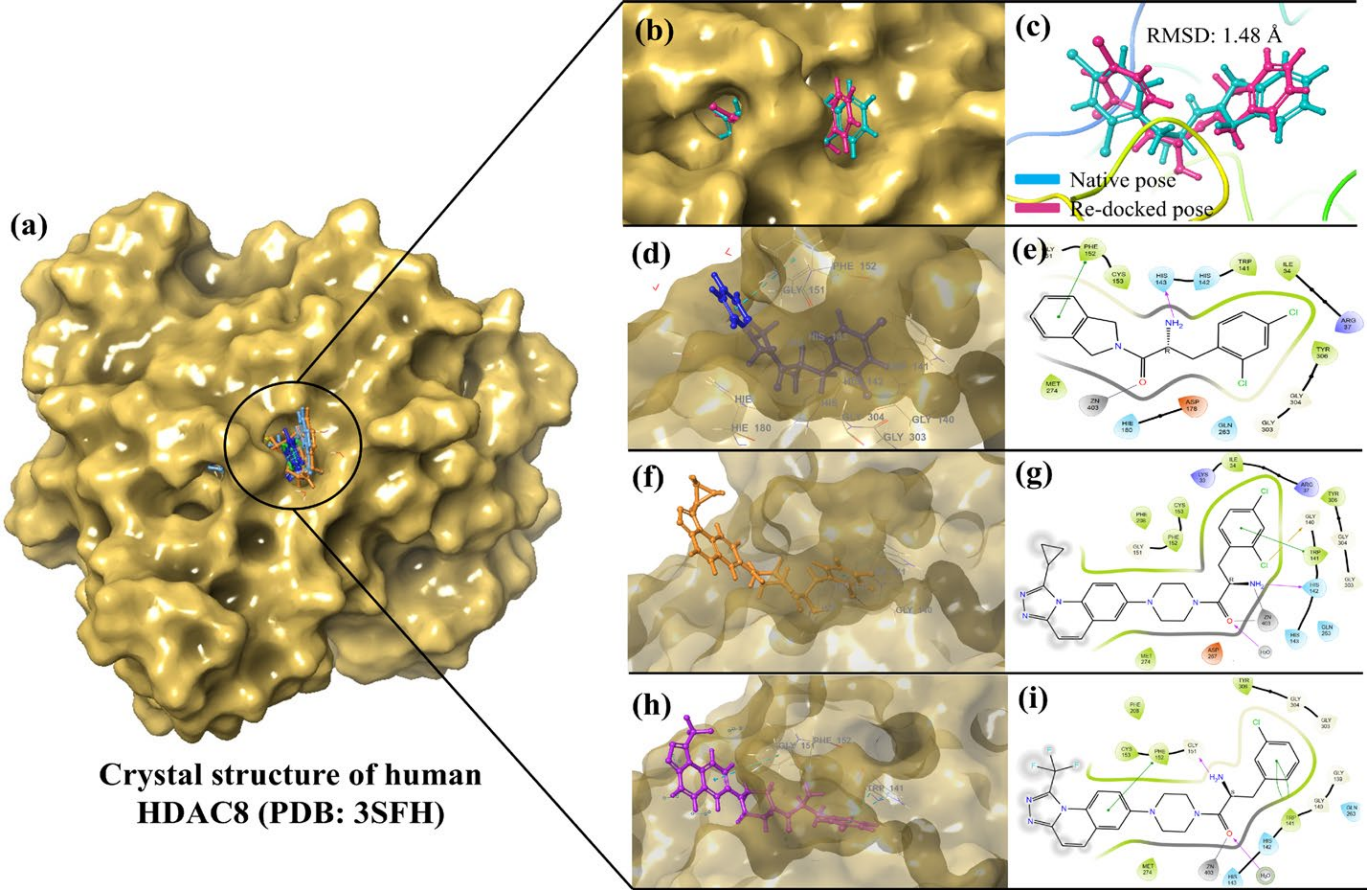
(**a**) Binding poses of compound **B** (native pose in blue and re-docked pose in pink), **9m** and **9r**; 3D (b) and 2D (c) images showing the interactions of the compound **B **(**d, e**); compound **9m **(**f, g**) and compound **9r **(**h, i**) with the catalytic zinc ion and catalytic triad residues.

HDAC8 protein is a zinc-dependent metalloenzyme that catalyzes the deacetylation of a lysine residue on the histone protein, resulting in gene repression^54^. The catalytic triad, which includes Asp101, His142, and Tyr306, interacts with the Zinc ions and forms a pseudo tetrahedral geometry that helps in stabilizing the transition state and activating the water molecular for hydrolysis of the acetyl groups, resulting in cleavage and forming acetate byproduct^55^. This catalytic function can be disrupted by preventing any of the interaction of catalytic triad with the zinc ions, thereby destabilizing the protein and preventing the hydrolysis of acetyl groups^54^. To determine the binding pose and find out the type of interactions the most active compounds, **9r** and **9m**, have, we decided to carry out molecular docking studies. Prior to the docking of the most active compounds, we first re-docked the Compound **B** (co-crystal ligand) into the generated active site grid to validate the docking protocol begin followed, and the re-dock score was found to be − 9.617 kcal/mol. The re-docked pose was then superimposed on the native ligand pose to observe the deviation from the experimental pose, and the RMSD was found to be 1.48 Å (Fig. 3a–c), showing the interactions of compound B in 3D and 2D images (Fig. 3b, c). This validates our protocol as the RMSD < 2.0 Å is considered to be acceptable.

From our molecular docking studies of Compound **B,** the most active compounds **9m** and **9r**, it was observed that the compound **B** was able to have a H-bond interaction with the His143 (1.08 Å); a π–π stacking interaction with Phe152 (4.55 Å); and a metal-coordination interaction was seen between the zinc ion and the oxygen atom from the amide group of the compound **B** with a distance of 1.75 Å (Fig. 3d, e). In the case of compound **9m**, (docking score: − 10.794 kcal/mol), a halogen interaction (3.36 Å) was seen between 2-chloro from dichloro ring of the ligand with Gly140 of the active site; a π–π interaction with Trp141 (4.22 Å); a H-bond interaction with one of the catalytic triads, His142 (1.84 Å); a H-bond interaction with water molecule (1.78 Å); and two metal-coordinations between zinc ion and the amide group of **9m** with a distance of 2.35 and 2.10 Å. The most active compound **9r**, has shown three π–π interactions, two with Trp141 (3.62 Å and 4.0 Å), one with Phe152 (5.38 Å); two H-bond interactions, one between the free energy scoring obtained is based on the sum of difference in minimization (ΔE_MM_), solvation (ΔG_solv_) and surface area (ΔG_SA_) energies as shown in Eq. 1. The binding free energy (ΔG_bind_) of the protein–ligand complex determines the ligand’s affinity towards the protein. More negative ΔG_bind_ indicates stronger binding affinity, while less negative indicates weaker affinity of the ligand. The ΔG_bind_ is calculated from the difference in the free energy of the complex (ΔG_complex_) and the sum of individual free energies of the ligand (ΔG_ligand_) and the protein (ΔG_protein_) as shown in Eq. 2^56^. The binding free energy values were calculated for the co-crystal ligand, **9m** and **9r,** and the results suggests that the **9m** and **9r** has ΔG _bind_ values better than the co-crystal, and likely to have similar affinity towards the protein (Table 3).

1 $$\Delta G = \Delta E_{MM} + \Delta G_{solv} + \Delta G_{SA}$$

2 $$\Delta G_{bind} = \Delta G_{complex} - \left( {\Delta G_{ligand} + \Delta G_{protein} } \right)$$

**Table 3:** MM-GBSA values of the co-crystal and the most potent compounds*

Compound	ΔG_coulomb_	ΔG_covalent_	ΔG_H-bond_	ΔG_Lipo_	ΔG_packing_	ΔG_solvent_	ΔG_vdW_	ΔG_bind_ (∑ΔG)
Compound **B**	− 20.42	6.40	− 0.58	− 35.27	− 2.63	31.38	− 43.24	− 64.36
**9m**	− 41.66	8.08	− 0.52	− 45.91	− 2.15	29.67	− 48.69	− 101.18
**9r**	− 30.37	11.92	− 0.47	− 37.72	− 1.36	37.59	− 45.83	− 66.24

*All ΔG values are reported in kcal /mol.

### Molecular dynamics and principal component analysis

**Fig. 4:**
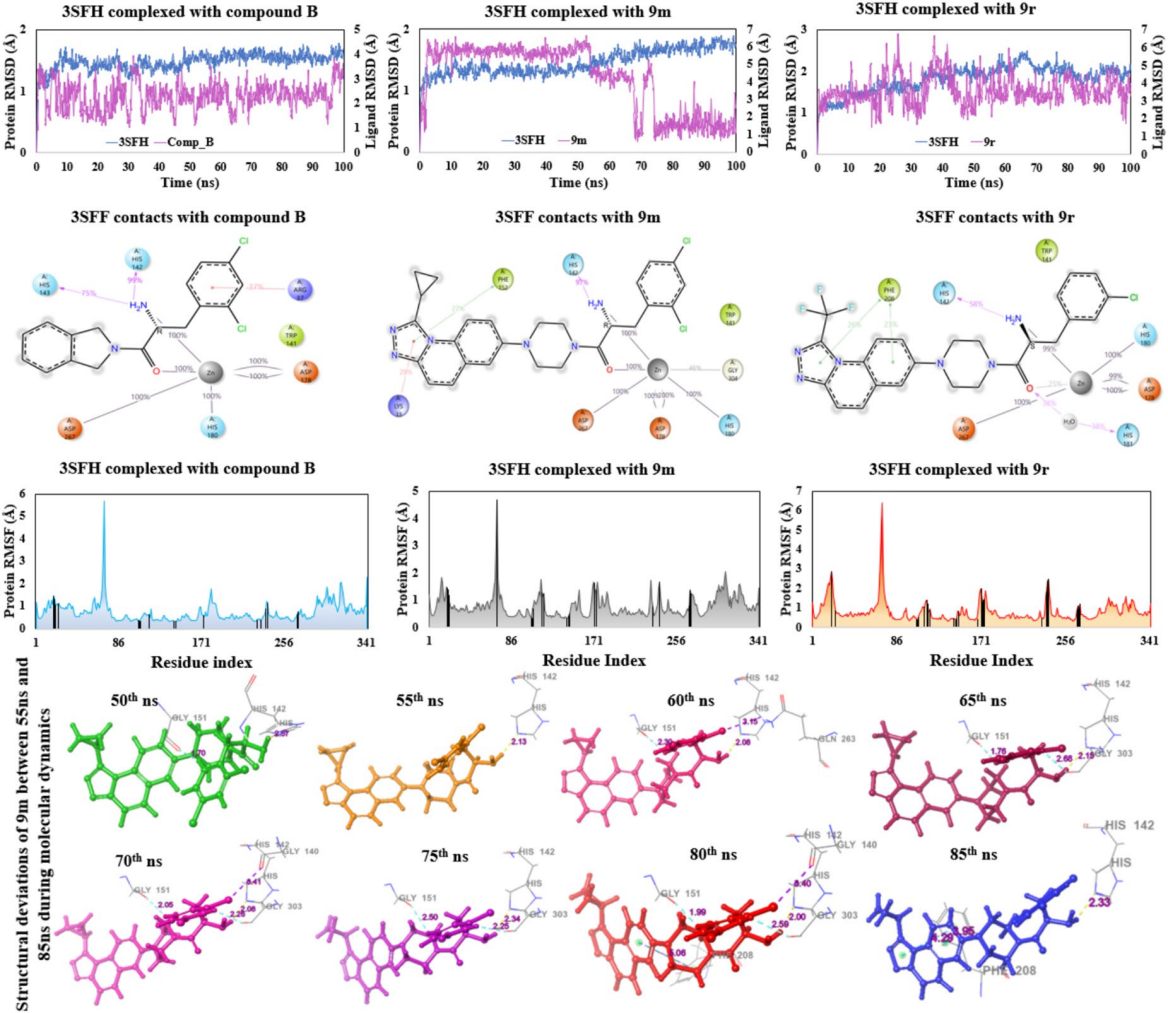
Representation of molecular dynamics results. The top row illustrates the RMSD plot, middle row illustrates the protein–ligand contacts, and the bottom row illustrates the RMSF plots with interacting residues (as black lines) of the compound **B**, **9m** and **9r,** respectively. At the bottom, conformational snapshots of compound **9m** are represented, showing the deviations from 50 to 80 ns during the molecular dynamics simulation.

Molecular dynamics (MD) studies were conducted on compounds **9m** and **9r**. For comparative analysis, Compound **B** was also subjected to MD studies for a duration of 100 ns. Compound **B** has demonstrated a stable RMSD plot for the entire simulation, ranging from 1.5 Å at the initial frame to 3.5 Å at the last frame (100^th^ ns). From the ligand–protein contacts, it is clear that the zinc ion, as expected, forms several metal-co-ordination interactions with the residues (Asp178:200%, His180: 100%, and Asp267: 100%) of the active site, and two additional interactions are also seen with the co-crystal ligand with 100% interaction contribution. One of the catalytic triads (His142) had shown 99% of H-bond interactions with the co-crystal ligand instead of the zinc ion, indicating it has shown no interaction with zinc ion for the entire MD run, which is essential for destabilizing the protein structure and inhibiting the enzymatic function. Additionally, His143 has shown 75% of H-bond interactions with the free amino group of the co-crystal ligand. In the case of compound **9m,** the deviations in the RMSD plot ranged from 5.6 Å initially to 6.4 Å until 55 ns, then decreased to 4.0 Å, deviating between 4.0 Å and 4.8 Å for the next 20 ns. Thereafter, the complex stabilized as the deviations were between 0.8 Å and 2.4 Å until the end. There were no significant changes found in the protein residues during the simulation. The deviations were due to the side chain of the ligand, not the protein, as evident from the RMSD as well as RMSF plots, both of which remained in the range of 0 and 2.0 Å. For the ease of interpretation and visualization, ligand poses of **9m** at an interval of 5 ns between 55 and 80 ns were extracted and the conformations revealed that the compound **9m** had never left the active site pocket and the deviations are due to the flexible 7-(piperazin-1-yl) side chain during the entire simulation. Furthermore, two H-bond interactions were seen after 55 ns each with Gly206 and Gly304, resulting in a sharp decrease in the RMSD from 4.8 Å to ~ 1.0 Å, indicating the ligand had attained a stable conformation (Fig. 4). The ligand–protein contacts are similar to co-crystal except the residue His143 as it has shown no interactions with **9m**. With the zinc ion, Asp178 has shown 200%, His180 has 100%, Asp267 has 100% and Gly304 has shown 45% of metal-coordination interactions. Two 100% interactions are also seen with the free amino group and amide oxygen of compound **9m**. Additionally, a π-cation interaction with Lys33 (28%), a H-bond interaction between free amino group of **9m** and His142 for over 97% and a π–π interaction between Phe152 and 1,2,4-triazolo[4,3-a] quinoline ring of **9m** (27%) is seen over the course of the simulation.

The RMSD of compound **9r** showed a stable plot ranging between 2.4 Å and 4.8 Å for the entire 100 ns simulation time, and the ligand–protein contacts show residual interactions similar to the co-crystal and compound **9m**. The zinc ion has metal-coordination interactions with Asp178 (199%), His180 (100%) and Asp267 (100%), additionally two interactions are also seen with the compound **9r** one with the amide oxygen (25%) and another with the free amino group (99%). One of the catalytic triad residue, His142 has 58% of H-bond interaction, another H-bond interaction (water-mediated) is seen with His181 (38%). Furthermore, two π–π interaction between Phe208 and 1,2,4-triazolo[4,3-a] quinoline ring is seen with 23% and 26% contribution. The RMSF plot in all the three complexes has shown stability as their value ranges from 0.4 Å to 2.0 Å for the entire duration and there were no huge fluctuations among all the residues except for Gln84 and Gly107 (Fig. 4). This is because, the residues between Gln84 and Gly107 are missing, and as a result the terminals show higher fluctuations during the MD run.

**Fig. 5:**
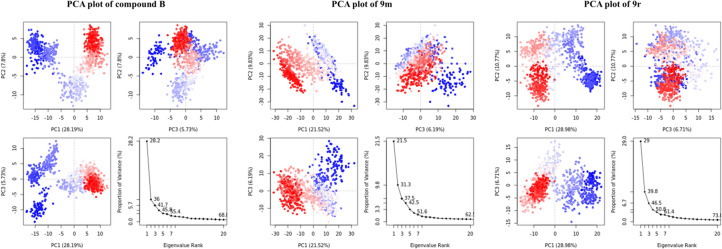
Representation of principal component analysis in which the fourth plot in each PCA is the scree plot, depicts the percentage of variance captured by each principal component. The coloured dots indicate changes in energy states of different conformations during the MD simulations.

The PCA analysis helps in analysing the change in conformations during the dynamic simulation. Each dot the in the plot represents a single conformation of the protein–ligand complex at a specific time; red dots represent a negative correlation with high energy, whereas blue dots indicate a positive correlation with low energy, and the white dots represent the transition states. The clustering of the dots represents similar conformations, while more considerable distances show transitional states. The screen plot in Fig. 5 shows the eigenvalue revealing the % of variance captured in the number of components. Our analysis reveals that the % variance captured by the first principal component (PC1) in the case of compound B is 28.2%, while for compounds **9m** and **9r**, the captured variance is 21.5% and 29% respectively, indicating most of the variance in the dataset is captured by the PC1. The elbow point in the scree plot shows a sharp decline in variance contribution after first few principal components. Thereafter, the subsequent components show a steep drop in variance contribution, indicating a larger number of PCs are required to explain higher variance. The pattern observed in our analysis shows more than 50% of the variance is captured within the first seven PCs for compound** B** and **9m** while > 60% is captured in the case of compound **9r**. For capturing more than 90% variance, a substantial number (> 20) of PCs are required for a comprehensive representation of the data.

### Structure–activity relationship studies

The structure–activity relationship (SAR) analysis of the synthesised HDAC8 inhibitors demonstrates that their inhibitory potency is highly influenced by the type and position of substituents on the core scaffold. The core scaffold with hydrogen atoms at both R_1_ and R_2_ **(9a)** exhibited only moderate activity, while the introduction of 3-chloro (**9d, 9i, 9n** and **9r**) or 2,4-dichloro (**9c, 9h, 9m** and **9q**) groups at the R_2_ position dramatically increased the activity by lowering the IC₅₀ values. Conversely, compounds containing electron-donating substituents such as methoxy (**9b, 9g, 9l** and **9p)** or hydroxy (**9f.** and **9k)** or unsubstituted (**9e, 9j, 9o, 9s, 9t** and **9u)** at R_2_ showed poor inhibitory activity irrespective of the R_1_ group. Further improvements were observed when hydrophobic groups such as methyl, cyclopropyl, or trifluoromethyl were introduced at R_1_ alongside halogenated R_2_ substituents. Notably, combinations like cyclopropyl or trifluoromethyl at R_1_ with 2,4-dichloro or 3-chloro at R_2_ produced the most potent inhibitors. Overall, these SAR observations indicate that the compounds bearing halogen atoms, especially 3-chloro (**9r)** or 2,4-dichloro (**9m)** groups at the R_2_ position, combined with hydrophobic or electron-withdrawing groups at R_1_, are crucial for maximising both HDAC8 potency and selectivity over other isoforms. Similar results were observed from the binding interactions in our molecular docking and stable RMSD plots from the MD simulations, proving the correlation between the enzyme activity results and docking.

### In-vitro cell growth inhibitory activity and clonogenic assay

**Fig. 6:**
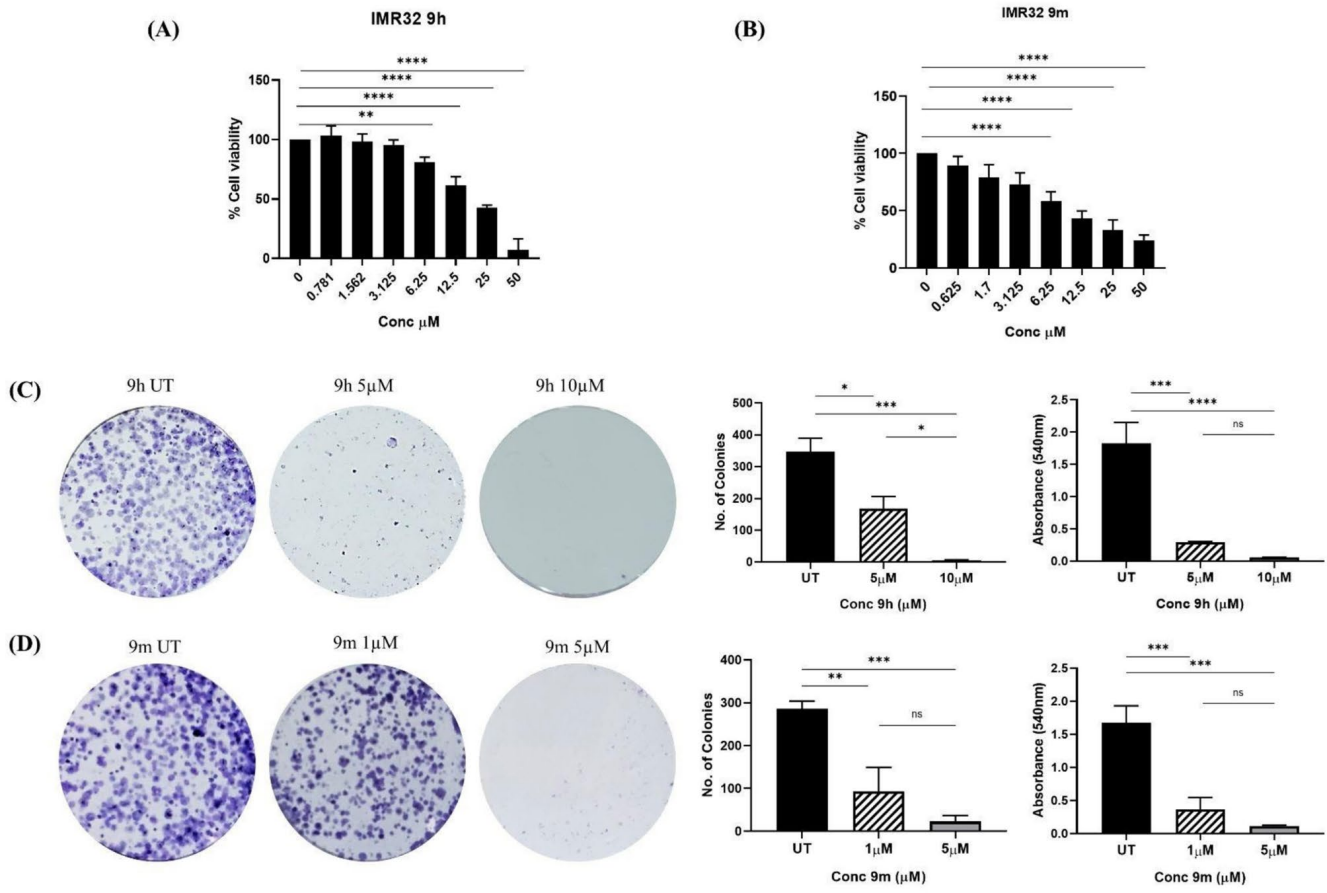
(**A**) Cell viability dose–response bar graphs for compound **9h** in IMR-32 cells by MTT assay (**B**) Cell viability dose–response bar graphs for compound **9m** in IMR-32 cells by MTT assay (**C**) Clonogenic assay of compound **9h** in IMR-32 cells at a concentration of 5 and 10 µM and quantification of the number of colonies and Absorbance of Crystal violet stain taken by the colonies in both control and treated cells (**D**) Clonogenic assay of compound **9m** in IMR-32 cells at a concentration of 1 and 5 µM and quantification of the number of colonies and Absorbance of Crystal violet stain taken by the colonies in both control and treated cells. The data are expressed as the mean ± SEM based on three independent experiments. (*p < 0.05, ** *p* < 0.01, *** *p* < 0.001, **** *p* < 0.0001).

We evaluated the phenotypic response of the active compounds in various cancerous cells; the results are reported in Table 4. We screened these compounds in HDAC8-dependent cell lines such as colon cancer (HCT116)^29^, breast cancer (MCF7)^37^, neuroblastoma cancer (IMR-32)^33^, and a normal cell line (HEK-293 T). Compounds **9h** and **9m** significantly impacted the viability of tested cell lines. Compound **9h** and **9m** exhibited the most promising anti-proliferative activity in IMR-32 cell line with an IC_50_ of 17.4 µM (Fig. 6A) and 9.92 µM (Fig. 6B), respectively. These two compounds (**9h, 9m**) were further evaluated to assess their long-term effect on IMR-32 cell line in a 2D-clonogenic assay, and the results are shown in Fig. 6C and D.

**Table 4:** The potency of selected compounds against cancer cell lines and the normal control cell line HEK293.

Entry	IMR-32 *IC_50_ (µM)	HCT116 *IC_50_ (µM)	MCF7 *IC_50_ (µM)	HEK293 *IC_50_ (µM)
**9c**	28.91 ± 8.06	47.32 ± 6.47	> 50	69.57 ± 10.70
**9d**	> 50	> 50	> 50	> 50
**9h**	17.38 ± 2.74	27.76 ± 2.06	36.83 ± 11.68	39.99 ± 9.05
**9i**	> 50	> 50	> 50	> 50
**9m**	9.92 ± 3.69	23.05 ± 12.47	24.12 ± 15.7	37.92 ± 10.19
**9n**	> 50	> 50	31.2	> 50
**9q**	> 50	> 50	> 50	> 50
**9r**	> 50	> 50	> 50	> 50
**SAHA**	1.62 ± 1.2	12.65 ± 13.97	10.07 ± 1.90	17.01 ± 5.82
**Trichostatin A**	8.24 ± 3.15	28.46 ± 20.81	31.77 ± 14.80	1.26 ± 0.28

*IC_50_ values are presented as the mean ± SD. The assay was performed in triplicate.

Indeed, in a dose-dependent manner (9–12 days), these compounds significantly affected the formation of colonies in IMR-32 cells. When IMR-32 cells were treated with compound **9h** at 5 and 10 µM concentrations, respectively, their capacity to form colonies was significantly reduced (Fig. 6C**).** Compound **9m** exhibited a similar pattern of results at 1 and 5 µM concentrations, respectively (Fig. 6D**).**

### Wound healing assay

**Fig. 7:**
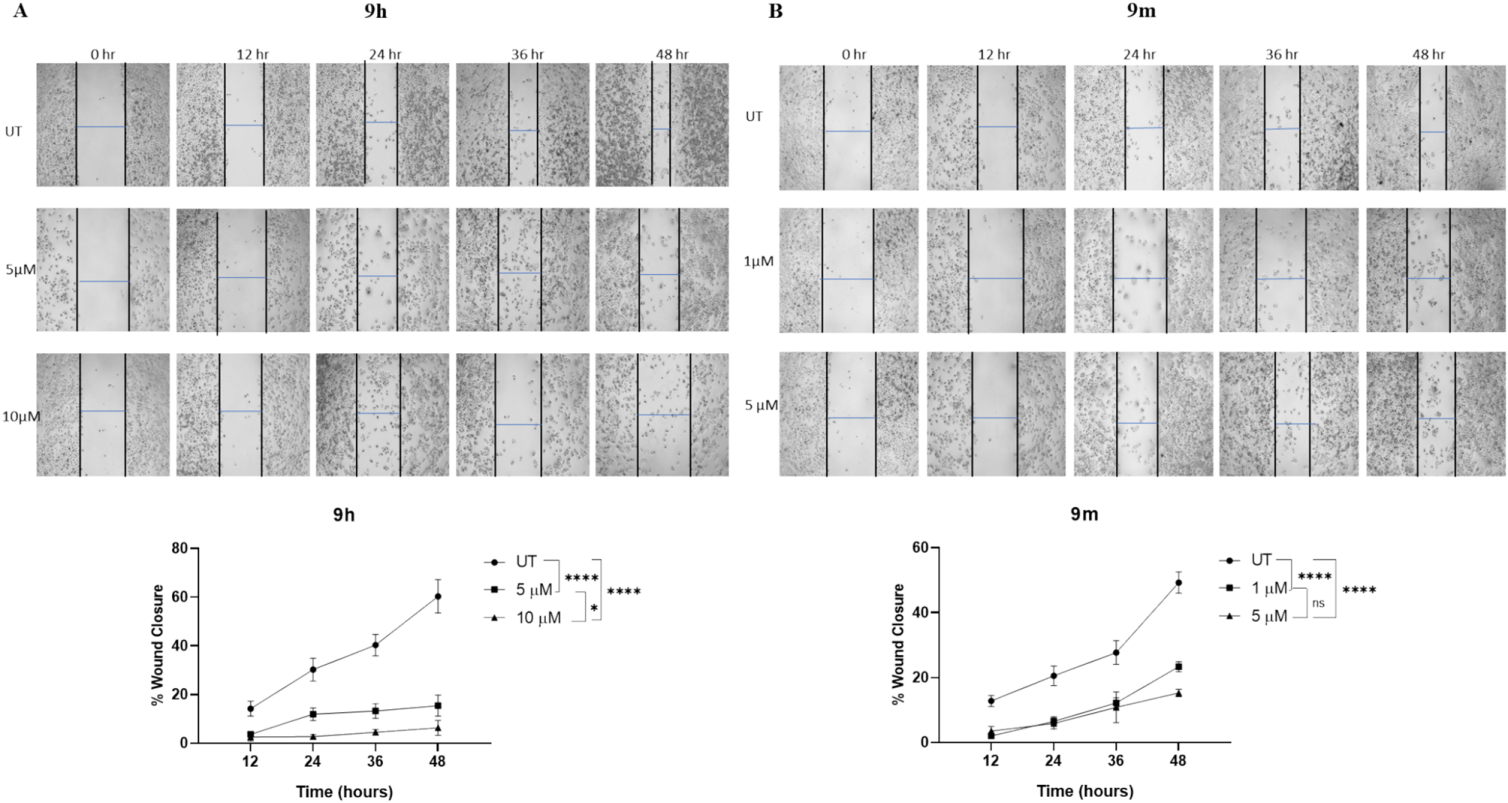
(**A**) IMR-32 cells were treated with the inhibitor **9h** for 48 h, and the percentage of wound closure was quantitatively analysed. (**B**) IMR-32 cells were treated with the inhibitor **9m** for 48 h, and % of wound closure was quantitatively analysed. The results are expressed as the mean ± SEM from triplicate experiments (*p < 0.05, ** *p* < 0.01, *** *p* < 0.001, **** *p* < 0.0001).

In subsequent studies, we investigated the effect of the **9h** and **9m** potent compounds on the metastatic progression of IMR-32 cells using a wound healing assay for up to 48 h. Percentage of wound healing was measured. Results are shown in Fig. 7. Both compounds slow down the rate of cell migration in IMR-32 cells at tested concentrations.

### Apoptosis assay and cell cycle arrest analysis

**Fig. 8:**
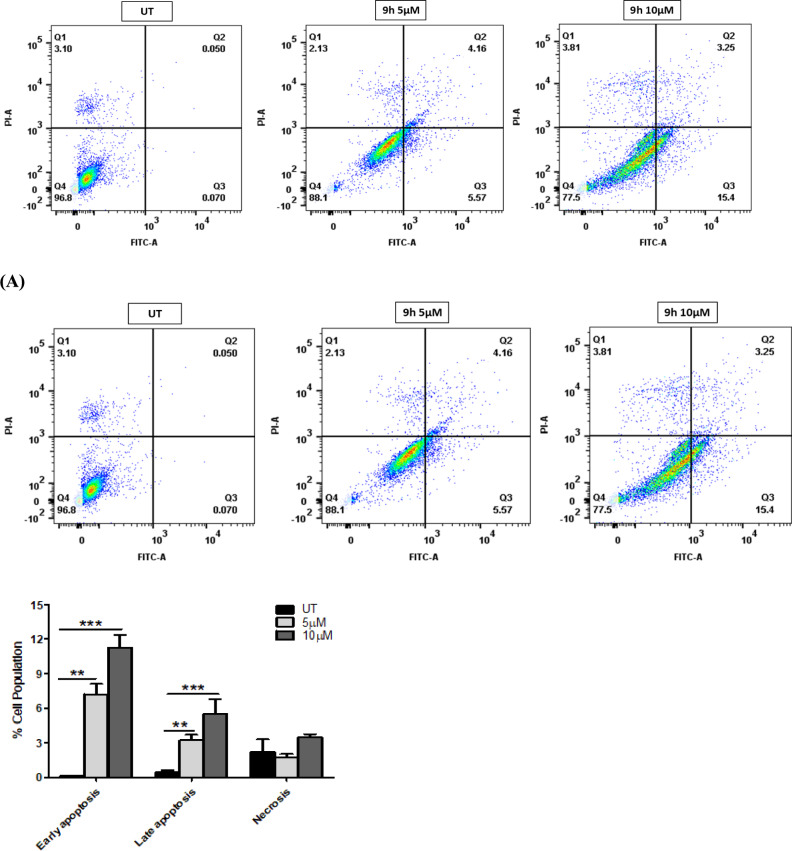

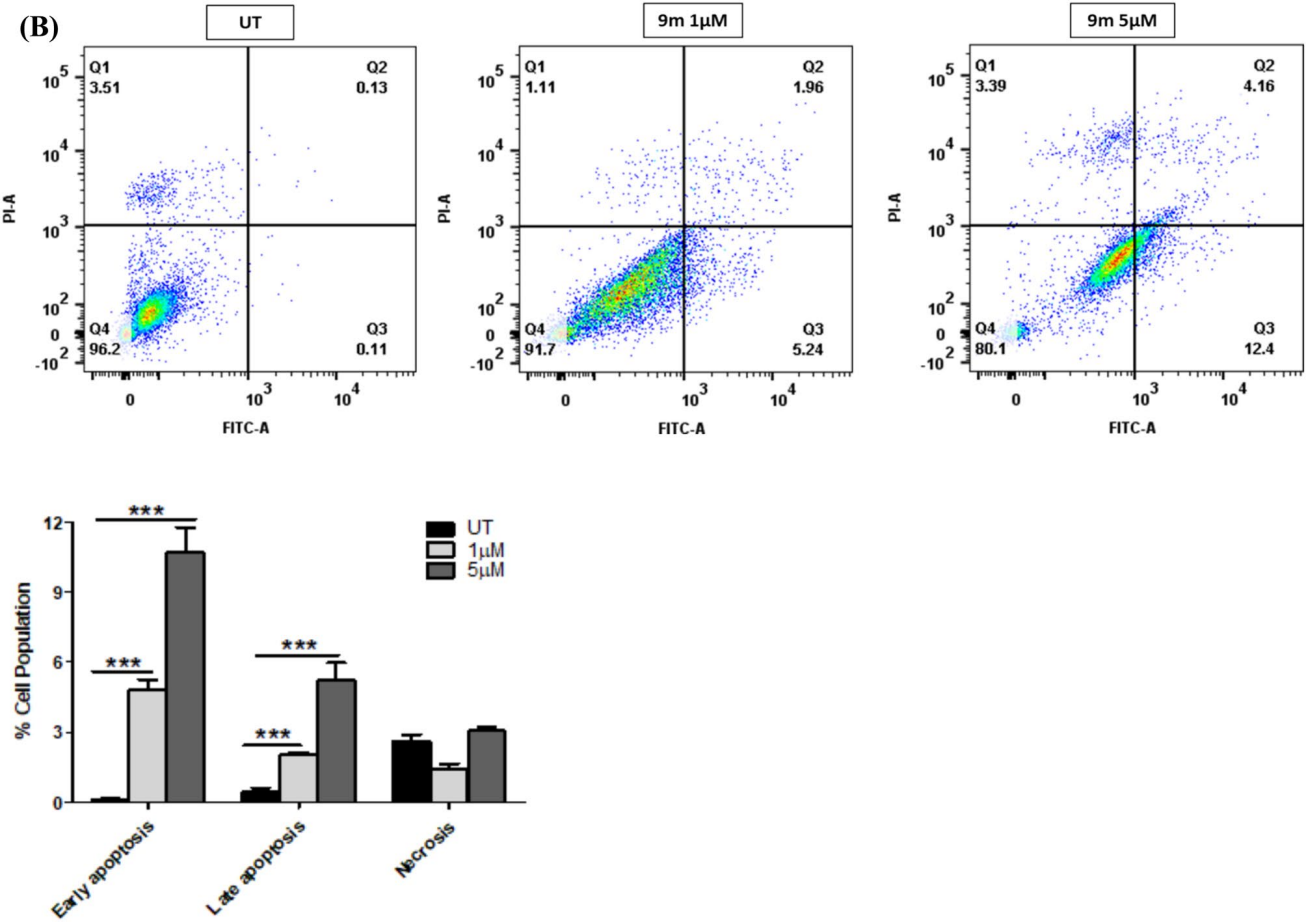

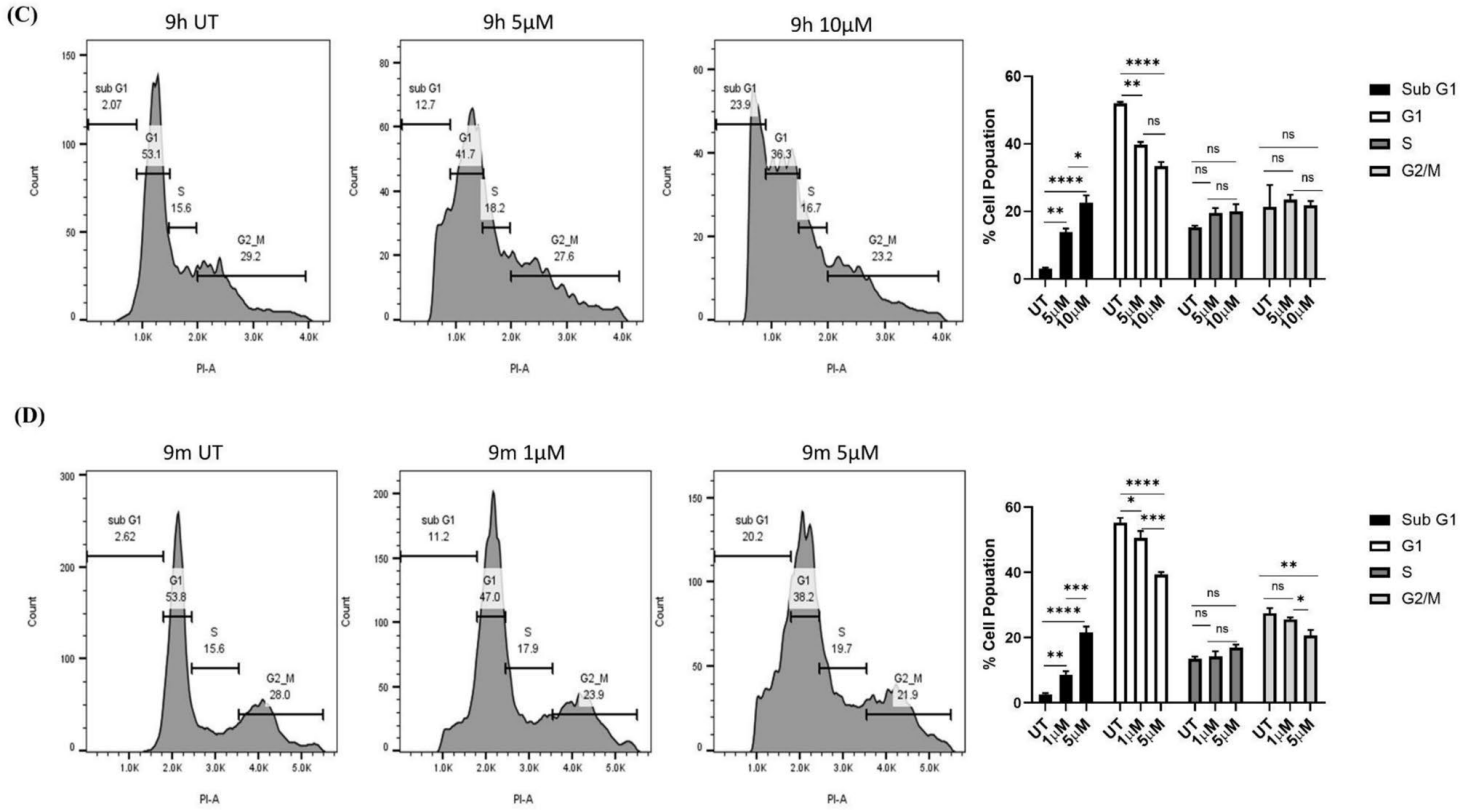
(**A**) The effect of **9h** treatment on apoptosis in IMR-32 cells as analysed using Annexin V-FITC/PI staining at 5 and 10 µM and quantification represented the percentage of apoptosis in treated cells compared to control cells. (**B**) The effect of **9m** treatment on apoptosis in IMR-32 cells as analysed using Annexin V-FITC/PI assay and quantification illustrating the percentage apoptosis/necrosis of treated cells as compared to control cells. (**C**) Effect of **9h** treatment on cell cycle progression in IMR-32 cells. Cells were treated with the compound **9h** at concentrations of 5 and 10 µM for 48 h and quantification of cell population in each phase of cell cycle (**D**) Effect of **9m** treatment on the cell cycle progression in IMR-32 cells. Cells were treated with the compound **9m** at concentrations of 1 and 5 µM for 48 h, and the quantification of cell population in each phase of cell cycle. The data are expressed as the mean ± SEM, based on three independent experiments. (*p < 0.05, ** *p* < 0.01, *** *p* < 0.001, **** *p* < 0.0001).

To analyze the cytotoxic activity (Apoptotic effect) of compounds **9h** and **9m** in IMR-32 cells, an Annexin V-FITC/propidium iodide assay was conducted, as described previously^57^. IMR-32 cells were treated with compound **9h** at 5 and 10 µM concentrations and compound **9m** at 1 and 5 µM concentrations for 48 h. Our study demonstrated that compounds **9h** and **9m** induced cell death through early apoptosis followed by late apoptosis stages in IMR-32 cells when compared to the control groups (Fig. 8). Using flow cytometry as previously described, we further evaluated the impact of **9h** and **9m** on the distribution of the cell cycle in IMR-32 cells^57^. Compound **9h** treatment of IMR-32 cells at 5 and 10 µM concentrations raised the proportion of cells in the Sub G1 phase by ~ 12% and 24%, respectively, while the control had 2% cells. Additionally, treatment with **9m** at 1 and 5 µM concentrations raised the percentage of G1 phase cells to around 11% and 20%, respectively, from approximately 3% in the control cells. These findings suggest that compounds **9h** and **9m** induce cell cycle arrest at the SubG1 phase in IMR-32 cells.

### Western blot analysis

**Fig. 9:**
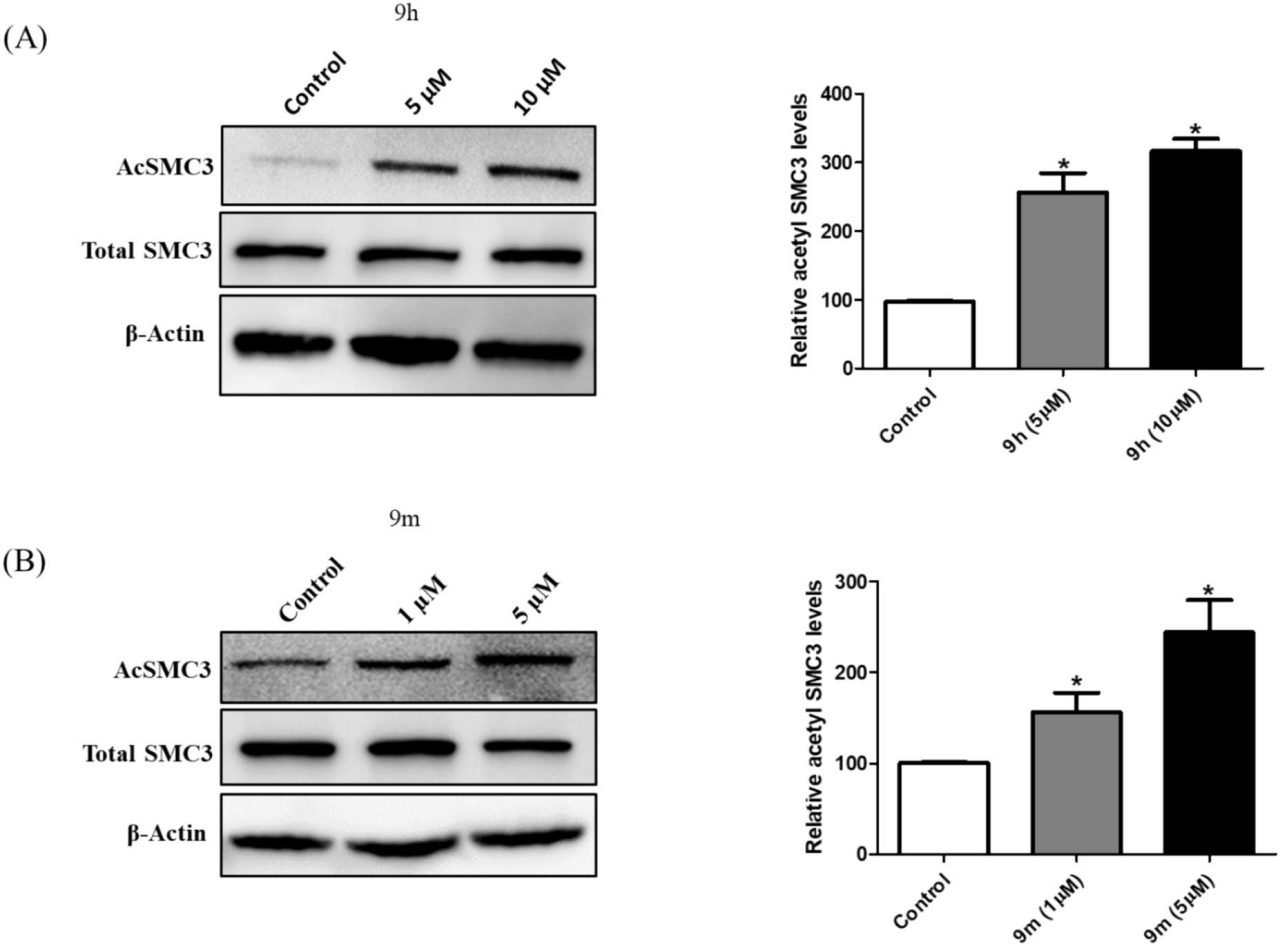
(**A**) Western Blot analysis of compound **9h** at indicated doses on the acetylation of SMC3 compared to total SMC3 and its densitometric analysis. (**B**) Western Blot analysis of compound **9m** at indicated doses on the acetylation of SMC3 compared to total SMC3 and its densitometric analysis. Densitometry is performed using ImageJ. Data are presented as the mean ± standard deviation of three independent experiments (∗ *p* < 0.05).

HDAC8 reverses the acetylation of the SMC3 done by ESCO1/2 during the S phase^58,59^. Inhibition of HDAC8 results in the accumulation of Ac-SMC3^60^. Hence, we evaluated the effect of **9h** and **9m** treatment on the acetylated form of SMC3 in IMR-32 cells. An increase in acetyl SMC3 expression was observed in IMR-32 cells upon treatment with compounds **9h** and **9m** compared to the total SMC3, and there is no change in the total SMC3 levels (Fig. 9A and B). The corresponding original blots are presented in the supporting information (Figs. S87 and S88).

## Conclusion

In this study, we identified a new class of potent, HDAC8-selective inhibitors based on the [1,2,4]Triazolo[4,3-a]quinoline scaffold. These compounds demonstrated strong selectivity for HDAC8 over other HDAC isoenzymes and exhibited significant anti-neuroblastoma activity. Molecular docking studies revealed key interactions within the HDAC8 active site. Mechanistic investigations further demonstrated that the anticancer effects are mediated through the modulation of SMC3 acetylation, confirming successful HDAC8 targeting. Additional assays, including cytotoxicity and cell migration studies, validated their promising therapeutic potential. These non-hydroxamate-based inhibitors show considerable promise for treating neuroblastoma with high HDAC8 expression. Continued structural optimization, guided by docking and dynamics insights, could enhance their potency and facilitate the development of lead compounds for future therapies.

## Methods

### Chemistry

All the chemical reagents and solvents were purchased from commercial suppliers and used without further purification. All reactions were performed under an argon atmosphere in oven-dried glassware with magnetic stirring. All reactions were monitored by thin-layer chromatography (TLC) on 0.25 mm silica gel plates (60GF-254), and developed chromatograms were visualized by UV light. Proton nuclear magnet resonance (^1^H NMR) spectra were recorded at 400 MHz on Varian or Bruker instrumentation. ^13^C NMR spectra were recorded at 101 MHz (Varian). Trimethylsiane (TMS) or residual solvent signals were used as an internal reference to calibrate the chemical shift δ values, and δ values were expressed in parts per million (ppm). Coupling constant *J* values were reported in hertz (Hz). The multiplicity of ^1^H NMR signals was reported as singlet (s), doublet (d), doublet of doublet (dd), triplet (t), quartert (q), broad singlet (brs), and multiplet (m). Liquid chromatography-mass spectra (LCMS) were obtained from Agilent 1100-LC/MSDVL. High-resolution mass spectroscopy (HRMS) analysis was conducted on a thermos orbitrap Q-Exactive mass spectrometer. In the case of TFA and HCl salts of compounds, HRMS and LC–MS values were calculated for the free form of the base. The purity of the final compounds was determined by high-performance liquid chromatography (HPLC). Purity was measured by UV absorbance at 254 nm.

### General procedures for preparation of intermediates

#### Synthesis of 6-Bromo-2-chloroquinoline (2)

The stirred solution of 6-bromoquinolin-2(1H)-one (15 g, 66.96 mmol, 1equiv.) and POCl_3_ (75 ml, 5 vol) was heated at 110 °C for 16 h. Reaction progress was monitored by TLC, and after completion of the starting material, the reaction mixture was concentrated under reduced pressure. The resultant crude was poured into a saturated sodium bicarbonate solution (300 mL) and filtered. The obtained solid was washed with water and dried under a high vacuum to afford the 13.4 g (yield: 82%) of intermediate **2** as an off-white solid.

^*1*^*H NMR (400 MHz, DMSO-d*_*6*_*)*: δ ppm 8.44 (d, *J* = 8.8 Hz, 1H), 8.37 (d, *J* = 2.0 Hz, 1H), 7.95–7.90 (m, 2H), 7.67 (d, *J* = 8.4 Hz, 1H); LCMS (ES, m/z) calculated for C_9_H_6_BrClN^+^[(M + H)^+^] 241.9, observed 241.9.

#### Synthesis of 6-Bromo-2-hydrazinylquinoline (3)

The stirred suspension of compound **2** (13 g, 53.94 mmol, 1 equiv.) and hydrazine hydrate (16.8 mL, 80% in H_2_O, 82.86 mmol, 5 equiv.) was heated at 100 °C for 16 h. Reaction progress was monitored by TLC, and after completion of the starting material, the reaction mixture was concentrated under reduced pressure. The resultant crude was diluted with ethyl acetate (150 mL) and washed with water. The organic layer was dried over Na_2_SO_4_, filtered, and concentrated under reduced pressure to afford the 10.5 g (yield: 82%) of intermediate **3** as an off-white solid.

^*1*^*H NMR (400 MHz, DMSO-d*_*6*_*)*: δ ppm 8.23 (brs, 1H), 7.88–7.84 (m, 2H), 7.57 (dd, *J* = 8.4, 2.4 Hz, 1H), 7.45 (d, *J* = 8.8 Hz, 1H), 6.88 (d, *J* = 8.8 Hz, 1H), 4.34 (brs, 2H); LCMS (ES, m/z) calculated for C_9_H_9_BrN_3_^+^ [(M + H)^+^] 238, observed 238.

#### Synthesis of compounds 4a-4d

The stirred suspension of compound **3** (2.5 g, 10.5 mmol, 1.0 equiv.) and corresponding carboxylic acid (5 vol) in a sealed tube was heated to 120 °C for 16 h. Reaction progress was monitored by TLC, and after completion of the starting material, the reaction mixture was concentrated under reduced pressure. The resultant crude was poured into ice-cold water, and then the aqueous phase was extracted with ethyl acetate (2 × 100 mL). The organic layer was dried over Na_2_SO_4_, filtered, and concentrated under reduced pressure to afford the crude. The resultant oil was purified by combi flash using 80% EtOAc in Hexane as an eluant to afford the intermediate compounds **4a-4d**.

##### Synthesis of 7-Bromo-[1,2,4] triazolo[4,3-a] quinoline (4a)

Intermediate **3** was treated with HCOOH according to general procedure **4a-4d**. Compound **4a** was obtained as 1.2 g (yield: 47%) of a pale yellow solid.

^*1*^*H NMR (400 MHz, DMSO-d*_*6*_*):* δ ppm 9.98 (s, 1H), 8.41 (d, *J* = 8.8 Hz, 1H), 8.31 (d, *J* = 2.4 Hz, 1H), 7.97 (dd, *J* = 8.4, 2.4 Hz, 1H), 7.78–7.76 (m, 2H); LCMS (ES, m/z) calculated for C_10_H_7_BrN_3_^+^ [(M + H)^+^] 247.9, observed 247.9.

##### Synthesis of 7-Bromo-1-methyl-[1,2,4]triazolo[4,3-a]quinoline (4b)

Intermediate **3** was treated with AcOH according to general procedure **4a-4d**. Compound **4b** was obtained as 1.6 g (yield: 58%) of a pale yellow solid.

^*1*^*H NMR (400 MHz, DMSO-d*_*6*_*)*: δ ppm 8.30–8.28 (m, 2H), 7.89 (dd, *J* = 9.2, 2.4 Hz, 1H), 7.72–7.70 (m, 2H), 3.05 (s, 3H); LCMS (ES, m/z) calculated for C_11_H_9_BrN_3_^+^ [(M + H)^+^] 261.9, observed 261.8.

##### Synthesis of 7-Bromo-1-cyclopropyl-[1,2,4]triazolo[4,3-a]quinoline (4c)

Intermediate **3** was treated with cyclopropyl carboxylic acid according to general procedure **4a-4d**. Compound **4c** was obtained as 1.3 g (yield: 46%) of a pale yellow solid.

^*1*^*H NMR (400 MHz, DMSO-d*_*6*_*)*: δ ppm 8.78 (d, *J* = 8.8 Hz, 1H), 8.31 (d, *J* = 2.4 Hz, 1H), 7.92 (dd, *J* = 9.2, 2.4 Hz, 1H), 7.75–7.69 (m, 2H), 2.63–2.61(m, 1H), 1.29–1.23 (m, 2H), 1.18–1.14 (m, 2H); LCMS (ES, m/z) calculated for C_13_H_11_BrN_3_^+^ [(M + H)^+^] 288.0, observed 287.9.

##### Synthesis of 7-Bromo-1-(trifluoromethyl)-[1,2,4]triazolo[4,3-a]quinoline (4d)

Intermediate **3** was treated with CF_3_COOH according to general procedure **4a-4d**. Compound **4d** was obtained as 1.5 g (yield: 45%) of a pale yellow solid.

^*1*^*H NMR (400 MHz, DMSO-d*_*6*_*)*: δ ppm 8.49 (d, *J* = 2.4 Hz, 1H), 8.13–7.99 (m, 4H); LCMS (ES, m/z) calculated for C_11_H_6_BrF_3_N_3_^+^ [(M + H)^+^] 315.9, observed 316.0.

#### General procedure for the synthesis of compounds 5a-5d

To a stirred solution of intermediate **4a-4d** (1.0 equiv.) in 1,4-dioxane (10 volumes), were added cesium carbonate (3.0 equiv.) and 1-Boc-piperazine (1.5 equiv.) and then degassed the reaction mixture with argon for 5 min. After degassing the reaction mixture were added Pd_2_(dba)_3_ (0.1 equiv.) and Xantphos (0.2 equiv.) and again degassed the reaction mixture for another 10 min. The resultant reaction mixture was stirred at 110 °C for 16 h. Progress of the reaction was monitored by TLC; after completion of the starting material, the reaction mixture was diluted with ethyl acetate and washed with water. The organic layer was dried over Na_2_SO_4_, filtered, and concentrated under reduced pressure to afford the crude compound. The resultant crude was purified by combi flash using 1% MeOH in DCM as an eluant to afford the intermediate compounds **5a-5d**.

##### Synthesis of ***tert***-Butyl 4-([1,2,4] triazolo[4,3-***a***] quinolin-7-yl) piperazine-1-carboxylate (5a)

Intermediate **4a** was treated with 1-Boc-piperazine according to general procedure **5a-5d**. Compound 5**a** was obtained as 1 g (yield: 76%) of a pale yellow solid.

^*1*^*H NMR (400 MHz, DMSO-d*_*6*_*)*: δ ppm 9.87 (s, 1H), 8.30 (d, *J* = 9.2 Hz, 1H), 7.68–7.67 (m, 2H), 7.52–7.47 (m, 2H), 3.53–3.51 (m, 4H), 3.24–3.22 (m, 4H), 1.44 (s, 9H); LCMS (ES, m/z) calculated for C_19_H_24_N_5_O_2_^+^ [(M + H)^+^] 354.1, observed 354.0.

##### Synthesis of ***tert***-Butyl 4-(1-methyl-[1,2,4]triazolo[4,3-***a***]quinolin-7-yl)piperazine-1-carboxylate (5b)

Intermediate **4b** was treated with 1-Boc-piperazine according to general procedure **5a-5d**. Compound 5**b** was obtained as 1.1 g (yield: 78%) of a pale yellow solid.

^*1*^*H NMR (400 MHz, DMSO-d*_*6*_*)*: δ ppm 8.21 (d, *J* = 9.2 Hz, 1H), 7.76–7.68 (m, 2H), 7.45–7.41 (m, 2H), 3.47–3.45 (m, 4H), 3.18–3.14 (m, 4H), 3.01 (s, 3H), 1.43 (s, 9H); LCMS (ES, m/z) calculated for C_20_H_26_N_5_O_2_^+^ [(M + H)^+^] 368.2, observed 368.2

##### Synthesis of ***tert***-Butyl 4-(1-cyclopropyl-[1,2,4]triazolo[4,3-***a***]quinolin-7-yl)piperazine-1-carboxylate (5c)

Intermediate **4c** was treated with 1-Boc-piperazine according to general procedure **5a-5d**. Compound 5**c** was obtained as 0.8 g (yield: 61%) of a pale yellow solid.

^*1*^*H NMR (400 MHz, DMSO-d*_*6*_*)*: δ ppm 8.64 (d, *J* = 9.2 Hz, 1H), 7.59 (d, *J* = 9.6 Hz, 1H), 7.53 (d, *J* = 9.6 Hz, 1H), 7.46 (d, *J* = 2.4 Hz, 1H), 7.40 (dd, *J* = 9.4, 3.0 Hz, 1H), 3.49–3.47 (m, 4H), 3.23–3.20 (m, 4H), 2.56–2.54 (m, 1H), 1.40 (s, 9H), 1.23–1.18 (m, 2H), 1.10–1.07 (m, 2H); LCMS (ES, m/z) calculated for C_22_H_28_N_5_O_2_^+^ [(M + H)^+^] 394.2, observed 394.2

##### Synthesis of ***tert***-Butyl 4-(1-(trifluoromethyl)-[1,2,4] triazolo[4,3-***a***]quinolin-7-yl)piperazine-1-carboxylate (5d)

Intermediate **4d** was treated with 1-Boc-piperazine according to general procedure **5a-5d**. Compound 5**d** was obtained as 0.9 g (yield: 69%) of a pale yellow solid.

^*1*^*H NMR (400 MHz, DMSO-d*_*6*_*)*: δ ppm 8.01–7.93 (m, 2H), 7.85 (d, *J* = 9.6 Hz, 1H), 7.63–7.59 (m, 2H), 3.53–3.51 (m, 4H), 3.25–3.23 (m, 4H), 1.42 (s, 9H); LCMS (ES, m/z) calculated for C_20_H_23_F_3_N_5_O_2_^+^ [(M + H)^+^] 422.1, observed 422.2.

#### Synthesis of general procedure for the synthesis of compounds 6a-6d

Trifluoroacetic acid (4 equiv.) was added to a stirred solution of compound **5a-5d** (1 equiv.) in dichloromethane (10 volumes) at °C and then stirred at RT for 4 h. Progress of the reaction was monitored by TLC, after completion of the starting material reaction mixture was concentrated under reduced pressure to afford the intermediate compounds **6a-6d** as TFA salt.

##### Synthesis of 4-([1,2,4] triazolo[4,3-***a***] quinolin-7-yl) piperazin-1-ium 2,2,2-trifluoroacetate (6a)

Intermediate **5a** was treated with TFA according to general procedure **6a-6d**. Compound **6a** was obtained as 0.9 g (yield: 87%) of a pale yellow solid.

^*1*^*H NMR (400 MHz, DMSO-d*_*6*_*)*: δ ppm 10.28 (s, 1H), 9.55 (brs, 1H), 8.50 (d, *J* = 9.2 Hz, 1H), 8.09 (d, *J* = 9.6 Hz, 1H), 7.85 (d, *J* = 9.6 Hz, 1H), 7.73–7.65 (m, 2H), 3.61–3.58 (m, 4H), 3.28–3.26 (m, 4H); LCMS (ES, m/z) calculated for C_14_H_16_N_5_^+^ [(M + H)^+^] 254.1, observed 254.0

##### Synthesis of 4-(1-methyl-[1,2,4]triazolo[4,3-***a***]quinolin-7-yl)piperazin-1-ium 2,2,2-trifluoroacetate (6b)

Intermediate **5b** was treated with TFA according to general procedure **6a-6d**. Compound **6b** was obtained as 1 g (yield: 91%) of a pale yellow solid.

^*1*^*H NMR (400 MHz, DMSO-d*_*6*_*)*: δ ppm 9.01 (brs, 1H), 8.29 (d, *J* = 9.2 Hz, 1H), 7.81 (d, *J* = 9.6 Hz, 1H), 7.74 (d, *J* = 9.6 Hz, 1H), 7.58–7.56 (m, 2H), 3.52–3.48 (m, 4H), 3.21–3.18 (m, 4H), 3.02 (s, 3H); LCMS (ES, m/z) calculated for C_15_H_18_N_5_^+^ [(M + H)^+^] 268.2, observed 268.1

##### Synthesis of 4-(1-cyclopropyl-[1,2,4]triazolo[4,3-***a***]quinolin-7-yl)piperazin-1-ium 2,2,2-trifluoroacetate (6c)

Intermediate **5c** was treated with TFA according to general procedure **6a-6d**. Compound **6c** was obtained as 0.75 g (yield: 91%) of a pale yellow solid.

^*1*^*H NMR (400 MHz, DMSO-d*_*6*_*)*: δ ppm 8.88 (brs, 1H), 8.75 (d, *J* = 9.2 Hz, 1H), 7.83 (d, *J* = 9.6 Hz, 1H), 7.69 (d, *J* = 9.6 Hz, 1H), 7.62 (d, *J* = 2.8 Hz, 1H), 7.54 (d, *J* = 9.6 Hz, 1H), 3.54–3.51 (m, 4H), 3.28–3.26 (m, 4H), 2.73–2.71 (m, 1H), 1.31–1.28 (m, 2H), 1.18–1.12 (m, 2H); LCMS (ES, m/z) calculated for C_17_H_20_N_5_^+^ [(M + H)^+^] 294.2, observed 294.2

##### Synthesis of 4-(1-(trifluoromethyl)-[1,2,4]triazolo[4,3-***a***]quinolin-7-yl)piperazin-1-ium 2,2,2-trifluoroacetate (6d)

Intermediate **5d** was treated with TFA according to general procedure **6a-6d**. Compound **6d** was obtained as 0.8 g (yield: 87%) of a pale yellow solid.

^*1*^*H NMR (400 MHz, DMSO-d*_*6*_*)*: δ ppm 8.13–8.01 (m, 2H), 7.88 (d, *J* = 9.6 Hz, 1H), 7.71–7.68 (m, 2H), 3.55–3.53 (m, 4H), 3.28–3.25 (m, 4H); LCMS (ES, m/z) calculated for C_15_H_15_F_3_N_5_^+^ [(M + H)^+^] 322.1, observed 322.1

#### Synthesis of general procedure for the synthesis of compounds 8a-8u

To the stirred solution of compound **6a-6d** (1 equiv.) in DMF (5 volumes) was treated with corresponding amino acid (1.1 equiv.) and DIPEA (3 equiv.) at 0 °C and then allowed the reaction mixture to stir at RT for 2 h. Progress of the reaction mixture was monitored by TLC. After completion of the reaction, the reaction mixture was poured into ice water, and the solid was obtained. The obtained solid was purified by combi flash using 2% MeOH in DCM as an eluent to afford the compounds **8a-8u**.

##### Synthesis of ***tert***-Butyl (R)-(1-(4-([1,2,4]triazolo[4,3-a]quinolin-7-yl)piperazin-1-yl)-1-oxo-3-phenylpropan-2-yl)carbamate (8a)

Intermediate **6a** was treated with commercial compound **7a** according to general procedure **8a-8u**. Compound **8a** was obtained as 0.1 g (yield: 71%) of a yellow solid.

^*1*^*H NMR (400 MHz, DMSO-d*_*6*_*)*: δ ppm 9.85 (s, 1H), 8.29 (d, *J* = 9.2 Hz, 4.0 Hz, 1H), 7.70–7.63 (m, 2H), 7.49–7.42 (m, 2H), 7.27–7.26 (m, 4H), 7.19–7.16 (m, 2H), 4.68–4.65 (m, 1H), 3.66–3.58 (m, 4H), 3.23–3.1 (m, 3H), 2.98–2.81 (m 3H), 1.33 (s, 9H); LCMS (ES, m/z) calculated for C_28_H_33_N_6_O_3_^+^ [(M + H)^+^] 501.2, observed 501.2.

##### Synthesis of ***tert***-Butyl (R)-(1-(4-([1,2,4]triazolo[4,3-a]quinolin-7-yl)piperazin-1-yl)-3-(4-methoxyphenyl)-1-oxopropan-2-yl)carbamate (8b)

Intermediate **6a** was treated with commercial compound **7c** according to general procedure **8a-8u**. Compound **8b** was obtained as 90 mg (yield: 60%) of a yellow solid.

^*1*^*H NMR (400 MHz, DMSO-d*_*6*_*)*: δ ppm 9.86 (s, 1H), 8.29 (d, *J* = 9.2 Hz, 1H), 7.70–7.64 (m, 2H), 7.48–7.45 (m, 1H), 7.41 (s, 1H), 7.18–7.14 (m, 3H), 6.82 (d, *J* = 8.8 Hz, 2H), 4.62–4.60 (m, 1H), 3.65–3.64 (m, 1H), 3.62 (s, 3H), 3.58–3.55 (m, 3H), 3.28–3.07 (m, 3H), 2.88–2.73 (m, 3H), 1.34 (s, 9H); LCMS (ES, m/z) calculated for C9_29_H_35_N_6_O_4_^+^ [(M + H)^+^] 531.2, observed 531.2.

##### Synthesis of ***tert***-Butyl (R)-(1-(4-([1,2,4]triazolo[4,3-a]quinolin-7-yl)piperazin-1-yl)-3-(2,4-dichlorophenyl)-1-oxopropan-2-yl)carbamate (8c)

Intermediate **6a** was treated with commercial compound **7d** according to general procedure **8a-8u**. Compound **8c** was obtained as 0.12 g (yield: 75%) of a yellow solid.

^*1*^*H NMR (400 MHz, DMSO-d*_*6*_*)*: δ ppm 9.86 (s, 1H), 8.30 (d, *J* = 9.2 Hz, 1H), 7.71–7.64 (m, 2H), 7.59–7.57 (m, 1H), 7.52–7.45 (m, 2H), 7.39–7.37 (m, 2H), 7.25–7.23 (m, 1H), 4.75–4.74 (m, 1H), 3.66–3.61 (m, 4H), 3.28–3.26 (m, 2H), 3.18–3.03 (m, 3H), 2.91–2.87 (m, 1H), 1.28 (s, 9H); LCMS (ES, m/z) calculated for C_28_H_31_Cl_2_N_6_O_3_^+^ [(M + H)^+^] 569.1, observed 569.1.

##### Synthesis of tert-Butyl (R)-(1-(4-([1,2,4]triazolo[4,3-a]quinolin-7-yl)piperazin-1-yl)-3-(3-chlorophenyl)-1-oxopropan-2-yl)carbamate (8d)

Intermediate **6a** was treated with commercial compound **7e** according to general procedure **8a-8u**. Compound **8d** was obtained as 0.1 g (yield: 67%) of a yellow solid.

^*1*^*H NMR (400 MHz, DMSO-d*_*6*_*)*: δ ppm 9.85 (s, 1H), 8.29 (d, *J* = 8.8 Hz, 1H), 7.69–7.64 (m, 2H), 7.49–7.43 (m, 2H), 7.35–7.23 (m, 5H), 4.67–4.65 (m, 1H), 3.68–3.58 (m, 4H), 3.28–3.08 (m, 4H), 3.92–2.88 (m, 1H), 2.85–2.82 (m, 1H), 1.31 (s, 9H); LCMS (ES, m/z) calculated for C_28_H_32_ClN_6_O_3_^+^ [(M + H)^+^] 535.2, observed 535.2.

##### Synthesis of ***tert***-Butyl (R)-(1-(4-(1-methyl-[1,2,4]triazolo[4,3-a]quinolin-7-yl)piperazin-1-yl)-1-oxo-3-phenylpropan-2-yl)carbamate (8e)

Intermediate **6b** was treated with commercial compound **7a** according to general procedure **8a-8u**. Compound **8e** was obtained as 0.1 g (yield: 71%) of a yellow solid.

^*1*^*H NMR (400 MHz, DMSO-d*_*6*_*)*: δ ppm 8.19 (d, *J* = 9.2 Hz, 1H), 7.63–7.54 (m, 2H), 7.44–7.43 (m, 1H), 7.38–7.35 (m, 1H), 7.28–7.24 (m, 4H), 7.21–7.17 (m, 2H), 4.69–4.64 (m, 1H), 3.66–3.61 (m, 4H), 3.21–3.18 (m, 3H), 3.02 (s, 3H), 2.95–2.83 (m, 3H), 1.33 (s, 9H); LCMS (ES, m/z) calculated for C_29_H_35_N_6_O_3_^+^ [(M + H)^+^] 515.2, observed 515.2.

##### Synthesis of *tert*-Butyl (R)-(3-(4-hydroxyphenyl)-1-(4-(1-methyl-[1,2,4]triazolo[4,3-a]quinolin-7-yl)piperazin-1-yl)-1-oxopropan-2-yl)carbamate (8f)

Intermediate **6b** was treated with commercial compound **7b** according to general procedure **8a-8u**. Compound **8f.** was obtained as 0.13 g (yield: 92%) of a yellow solid.

^*1*^*H NMR (400 MHz, DMSO-d*_*6*_*)*: δ ppm 9.08 (s, 1H), 8.48 (d, *J* = 9.6 Hz, 1H), 8.29 (d, *J* = 9.6 Hz, 1H), 7.65 (d, *J* = 9.2 Hz, 1H), 7.54 (dd, *J* = 9.4, 2.6 Hz, 1H), 7.43 (d, *J* = 2.8 Hz, 1H), 7.12–7.08 (m, 3H), 6.68 (d, *J* = 8.4 Hz, 2H), 4.62–4.58 (m, 1H), 3.76–3.71 (m, 1H), 3.64–3.54 (m, 1H), 3.48–3.33 (m, 1H), 3.28–3.26 (m, 1H), 3.24–3.21 (m, 1H), 3.12 (s, 3H), 3.08–3.05 (m, 2H), 3.01–2.98 (m, 1H), 2.85–2.76 (m, 1H), 2.62–2.58 (m, 1H), 1.29 (s, 9H); LCMS (ES, m/z) calculated for C_29_H_35_N_6_O_4_^+^ [(M + H)^+^] 531.3, observed 531.4.

##### Synthesis of *tert*-Butyl (R)-(3-(4-methoxyphenyl)-1-(4-(1-methyl-[1,2,4]triazolo[4,3-a]quinolin-7-yl)piperazin-1-yl)-1-oxopropan-2-yl)carbamate (8g)

Intermediate **6b** was treated with commercial compound **7c** according to general procedure **8a-8u**. Compound **8g** was obtained as 0.11 g (yield: 73%) of a yellow solid.

^*1*^*H NMR (400 MHz, DMSO-d*_*6*_*)*: δ ppm 8.38 (d, *J* = 9.6 Hz, 1H), 8.16 (d, *J* = 9.6 Hz, 1H), 7.66 (d, *J* = 9.2 Hz, 1H), 7.54 (dd, *J* = 9.4, 2.6 Hz, 1H), 7.43 (d, *J* = 3.2 Hz, 1H), 7.14–7.13 (m, 3H), 6.82 (d, *J* = 6.8 Hz, 2H), 4.62–4.56 (m, 1H), 3.78–3.75 (m, 1H), 3.68–3.62 (m, 1H), 3.59 (s, 3H), 3.48–3.42 (m, 1H), 3.38–3.33 (m, 1H), 3.19–3.16 (m, 1H), 3.18 (s, 3H), 3.16–3.05 (m, 4H), 2.46–2.42 (m, 1H), 1.31(s, 9H); LCMS (ES, m/z) calculated for C_30_H_37_N_6_O_4_^+^ [(M + H)^+^] 545.2, observed 545.2.

##### Synthesis of *tert*-Butyl (R)-(3-(2,4-dichlorophenyl)-1-(4-(1-methyl-[1,2,4]triazolo[4,3-a]quinolin-7-yl)piperazin-1-yl)-1-oxopropan-2-yl)carbamate (8h)

Intermediate **6b** was treated with commercial compound **7d** according to general procedure **8a-8u**. Compound **8h** was obtained as 0.1 g (yield: 62%) of a yellow solid.

^*1*^*H NMR (400 MHz, DMSO-d*_*6*_*)*: δ ppm 8.20 (d, *J* = 9.2 Hz, 1H), 7.63–7.55 (m, 3H), 7.47–7.46 (m, 1H), 7.42–7.34 (m, 3H), 7.25–7.23 (m, 1H), 4.77–4.71 (m, 1H), 3.67–3.61 (m, 4H), 3.28–3.12 (m, 5H), 3.06 (s, 3H), 2.93–2.87 (m, 1H), 1.28 (s, 9H); LCMS (ES, m/z) calculated for C_29_H_33_Cl_2_N_6_O_3_^+^ [(M + H)^+^] 583.1, observed 583.1.

##### Synthesis of *tert*-Butyl (R)-(3-(3-chlorophenyl)-1-(4-(1-methyl-[1,2,4]triazolo[4,3-a]quinolin-7-yl)piperazin-1-yl)-1-oxopropan-2-yl)carbamate (8i)

Intermediate **6b** was treated with commercial compound **7e** according to general procedure **8a-8u**. Compound **8i** was obtained as 0.12 g (yield: 80%) of a yellow solid.

^*1*^*H NMR (400 MHz, DMSO-d*_*6*_*)*: δ ppm 8.48 (d, *J* = 9.2 Hz, 1H), 8.21 (d, *J* = 9.6 Hz, 1H), 7.87 (d, *J* = 9.6 Hz, 1H), 7.52 (dd, *J* = 9.6, 2.7 Hz, 1H), 7.45 (d, *J* = 2.7 Hz, 1H), 7.31–7.28 (m, 3H), 7.18–7.16 (m, 2H), 4.75–4.68 (m, 1H), 3.76–3.61 (m, 2H), 3.58–3.52 (m, 1H), 3.38–3.36 (m, 1H), 3.27–3.25 (m, 1H), 3.16–3.14 (m, 1H), 3.16 (s, 3H), 3.14–3.11 (m, 3H), 2.66–2.63 (m, 1H), 1.29 (s, 9H); LCMS (ES, m/z) calculated for C_29_H_34_ClN_6_O_3_^+^ [(M + H)^+^] 549.2, observed 549.3.

##### Synthesis of *tert*-Butyl (R)-(1-(4-(1-cyclopropyl-[1,2,4]triazolo[4,3-a]quinolin-7-yl)piperazin-1-yl)-1-oxo-3-phenylpropan-2-yl)carbamate (8j)

Intermediate **6c** was treated with commercial compound **7a** according to general procedure **8a-8u**. Compound **8j** was obtained as 90 mg (yield: 64%) of a yellow solid.

^*1*^*H NMR (400 MHz, DMSO-d*_*6*_*)*: δ ppm 8.67 (d, *J* = 9.2 Hz, 1H), 7.63 (d, *J* = 9.6 Hz, 1H), 7.56 (d, *J* = 9.6 Hz, 1H), 7.45 (d, *J* = 2.8 Hz, 1H), 7.40 (dd, *J* = 9.2, 2.8 Hz, 1H), 7.28–7.26 (m, 4H), 7.21–7.17 (m, 2H), 4.68–4.65 (m, 1H), 3.67–3.61 (m, 4H), 3.28–3.12 (m, 3H), 2.98–2.82 (m, 3H), 2.59–2.57 (m, 1H), 1.33 (s, 9H), 1.28–1.26 (m, 2H), 1.15–1.11 (m, 2H); LCMS (ES, m/z) calculated for C_31_H_37_N_6_O_3_^+^ [(M + H)^+^] 541.3, observed 541.2

##### Synthesis of *tert*-Butyl (R)-(1-(4-(1-cyclopropyl-[1,2,4]triazolo[4,3-a]quinolin-7-yl)piperazin-1-yl)-3-(4-hydroxyphenyl)-1-oxopropan-2-yl)carbamate (8k)

Intermediate **6c** was treated with commercial compound **7b** according to general procedure **8a-8u**. Compound **8k** was obtained as 0.1 g (yield: 71%) of a yellow solid.

^*1*^*H NMR (400 MHz, DMSO-d*_*6*_*)*: δ ppm 9.16 (s, 1H), 8.67 (d, *J* = 9.2 Hz, 1H), 7.63 (d, *J* = 9.6 Hz, 1H), 7.56 (d, *J* = 9.6 Hz, 1H), 7.44 (d, *J* = 2.8 Hz, 1H), 7.40 (dd, *J* = 9.2, 2.8 Hz, 1H), 7.12–7.03 (m, 3H), 6.65 (d, *J* = 8.4 Hz, 2H), 4.60–4.57 (m, 1H), 3.62–3.54 (m, 4H), 3.28–3.11 (m, 3H), 2.94–2.92 (m, 1H), 2.81–2.68 (m, 2H), 2.59–2.57 (m, 1H), 1.34 (s, 9H), 1.25–1.22 (m, 2H), 1.15–1.11 (m, 2H); LCMS (ES, m/z) calculated for C_31_H_37_N_6_O_4_^+^ [(M + H)^+^] 557.2, observed 557.2.

##### Synthesis of tert-Butyl (R)-(1-(4-(1-cyclopropyl-[1,2,4]triazolo[4,3-a]quinolin-7-yl)piperazin-1-yl)-3-(4-methoxyphenyl)-1-oxopropan-2-yl)carbamate (8l)

Intermediate **6c** was treated with commercial compound **7c** according to general procedure **8a-8u**. Compound **8l** was obtained as 0.1 g (yield: 86%) of a yellow solid.

^*1*^*H NMR (400 MHz, DMSO-d*_*6*_*)*: δ ppm 8.78 (d, *J* = 9.2 Hz, 1H), 8.10 (d, *J* = 9.6 Hz, 1H), 7.63 (d, *J* = 9.6 Hz, 1H), 7.54 (dd, *J* = 9.2, 2.0 Hz, 1H), 7.42 (d, *J* = 2.4 Hz, 1H), 7.17–7.14 (m, 3H), 6.94 (d, *J* = 8.6 Hz, 2H), 4.65–4.62 (m, 1H), 3.75–3.73 (m, 1H), 3.71–3.69 (m, 1H), 3.64 (s, 3H), 3.42–3.36 (m, 1H), 3.28–3.26 (m, 1H), 3.01–2.98 (m, 5H), 2.66–2.63 (m, 1H), 2.58–2.56 (m, 1H), 1.29 (s, 9H), 1.27–1.25 (m, 2H), 1.14–1.12 (m, 2H); LCMS (ES, m/z) calculated for C_32_H_39_N_6_O_4_ + [(M + H) +] 571.2, observed 571.3.

##### Synthesis of *tert*-Butyl (R)-(1-(4-(1-cyclopropyl-[1,2,4]triazolo[4,3-a]quinolin-7-yl)piperazin-1-yl)-3-(2,4-dichlorophenyl)-1-oxopropan-2-yl)carbamate (8m)

Intermediate **6c** was treated with commercial compound **7d** according to general procedure **8a-8u**. Compound **8m** was obtained as 0.11 g (yield: 73%) of a yellow solid.

^*1*^*H NMR (400 MHz, DMSO-d*_*6*_*)*: δ ppm 8.68 (d, *J* = 9.2 Hz, 1H), 7.64 (d, *J* = 9.6 Hz, 1H), 7.56–7.54 (m, 2H), 7.48–7.35 (m, 4H), 7.24 (d, *J* = 8.8 Hz, 1H), 4.76–4.74 (m, 1H), 3.82–3.78 (m, 2H), 3.48–3.43 (m, 2H), 3.32–3.12 (m, 6H), 2.62–2.58 (m, 1H), 1.44 (s, 9H), 1.28–1.25 (m, 2H), 1.14–1.12 (m, 2H) LCMS (ES, m/z) calculated for C_31_H_35_Cl_2_N_6_O_3_^+^ [(M + H)^+^] 609.2, observed 609.2.

##### Synthesis of *tert*-Butyl (R)-(3-(3-chlorophenyl)-1-(4-(1-cyclopropyl-[1,2,4]triazolo[4,3-a]quinolin-7-yl)piperazin-1-yl)-1-oxopropan-2-yl)carbamate (8n)

Intermediate **6c** was treated with commercial compound **7e** according to general procedure **8a-8u**. Compound **8n** was obtained as 90 mg (yield: 64%) of a yellow solid.

^*1*^*H NMR (400 MHz, DMSO-d*_*6*_*)*: δ ppm 8.68 (d, *J* = 9.2 Hz, 1H), 7.63 (d, *J* = 10.0 Hz, 1H), 7.57 (d, *J* = 9.6 Hz, 1H), 7.48 (d, *J* = 2.4 Hz, 1H), 7.42 (dd, *J* = 9.2, 2.4 Hz, 1H), 7.36–7.23 (m, 5H), 4.68–4.66 (m, 1H), 3.69–3.62 (m, 4H), 3.28–3.08 (m, 4H), 2.96–2.92 (m, 1H), 2.86–2.78 (m, 1H), 2.59–2.57 (m, 1H), 1.32 (s, 9H), 1.26–1.22 (m, 2H), 1.15–1.11 (m, 2H); LCMS (ES, m/z) calculated for C_31_H_36_ClN_6_O_3_^+^ [(M + H)^+^] 575.2, observed 575.2.

##### Synthesis of *tert*-Butyl (R)-(1-oxo-3-phenyl-1-(4-(1-(trifluoromethyl)-[1,2,4] triazolo[4,3-a]quinolin-7-yl)piperazin-1-yl)propan-2-yl)carbamate (8o)

Intermediate **6d** was treated with commercial compound **7a** according to general procedure **8a-8u**. Compound **8o** was obtained as 90 mg (yield: 64%) of a yellow solid.

^*1*^*H NMR (400 MHz, DMSO-d*_*6*_*)*: δ ppm 8.02–7.96 (m, 3H), 7.86 (d, *J* = 9.6 Hz, 1H), 7.66–7.64 (m, 2H), 7.20–7.16 (m, 5H), 4.69–4.65 (m, 1H), 3.87–3.78 (m, 2H), 3.72–3.58 (m, 3H), 3.48–3.42 (m, 1H), 3.22–3.18 (m, 1H), 2.98–2.82 (m, 3H), 1.36 (s, 9H), LCMS (ES, m/z) calculated for C_29_H_32_F_3_N_6_O_3_^+^ [(M + H)^+^] 569.2, observed 569.2.

##### Synthesis of *tert*-Butyl (R)-(3-(4-methoxyphenyl)-1-oxo-1-(4-(1-(trifluoromethyl)-[1,2,4] triazolo[4,3-a]quinolin-7-yl)piperazin-1-yl)propan-2-yl)carbamate (8p)

Intermediate **6d** was treated with commercial compound **7c** according to general procedure **8a-8u**. Compound **8p** was obtained as 0.1 g (yield: 71%) of a yellow solid.

^*1*^*H NMR (400 MHz, DMSO-d*_*6*_*)*: δ ppm 7.99–7.93 (m, 2H), 7.85 (d, *J* = 9.6 Hz, 1H), 7.58–7.55 (m, 2H), 7.18–7.15 (m, 3H), 6.82 (d, *J* = 8.8 Hz, 2H), 4.62–4.60 (m, 1H), 3.68–3.64 (m, 2H), 3.62 (s, 3H), 3.58–3.56 (m, 2H), 3.28–3.22 (m, 2H), 3.18–3.16 (m, 1H), 2.92–2.72 (m, 3H), 1.34 (s, 9H); LCMS (ES, m/z) calculated for C_30_H_34_F_3_N_6_O_4_^+^ [(M + H)^+^] 599.2, observed 599.2.

##### Synthesis of *tert*-Butyl (R)-(3-(2,4-dichlorophenyl)-1-oxo-1-(4-(1-(trifluoromethyl)-[1,2,4] triazolo[4,3-a]quinolin-7-yl)piperazin-1-yl)propan-2-yl)carbamate (8q)

Intermediate **6d** was treated with commercial compound **7d** according to general procedure **8a-8u**. Compound **8q** was obtained as 0.12 g (yield: 80%) of a yellow solid.

^*1*^*H NMR (400 MHz, DMSO-d*_*6*_*)*: δ ppm 8.01–7.92 (m, 2H), 7.86 (d, *J* = 9.6 Hz, 1H), 7.62–7.58 (m, 3H), 7.38–7.32 (m, 2H), 7.26–7.23 (m, 1H), 4.75–4.72 (m, 1H), 3.68–3.62 (m, 4H), 3.28–3.16 (m, 2H), 3.08–3.02 (m, 1H), 2.68–2.65 (m, 3H), 1.28 (s, 9H); LCMS (ES, m/z) calculated for C_29_H_30_Cl_2_F_3_N_6_O_3_^+^ [(M + H)^+^] 637.2, observed 637.3.

##### Synthesis of *tert*-Butyl (R)-(3-(3-chlorophenyl)-1-oxo-1-(4-(1-(trifluoromethyl)-[1,2,4]triazolo[4,3-a]quinolin-7-yl)piperazin-1-yl)propan-2-yl)carbamate (8r)

Intermediate **6d** was treated with commercial compound **7e** according to general procedure **8a-8u**. Compound **8r** was obtained as 0.1 g (yield: 71%) of a yellow solid.

^*1*^*H NMR (400 MHz, DMSO-d*_*6*_*)*: δ ppm 8.01–7.94 (m, 2H), 7.86 (d, *J* = 9.6 Hz, 1H), 7.62–7.58 (m, 2H), 7.38–7.23 (m, 5H), 4.70–4.64 (m, 1H), 3.71–3.62 (m, 4H), 3.28–3.16 (m, 3H), 2.92–2. 82 (m, 3H), 1.31 (s, 9H); LCMS (ES, m/z) calculated for C_29_H_31_ClF_3_N_6_O_3_^+^ [(M + H)^+^] 603.2, observed 603.3.

##### Synthesis of *tert*-Butyl (R)-3-(4-(1-methyl-[1,2,4] triazolo[4,3-a]quinolin-7-yl)piperazine-1-carbonyl)-3,4-dihydroisoquinoline-2(1H)-carboxylate (8s)

Intermediate **6b** was treated with commercial compound **7f.** According to general procedure **8a-8u**. Compound **8s** was obtained as 0.12 g (yield: 75%) of a yellow solid.

^*1*^*H NMR (400 MHz, DMSO-d*_*6*_*)*: δ ppm 8.06 (d, *J* = 9.8 Hz, 1H), 7.55 (d, *J* = 9.6 Hz, 1H), 7.43–7.38 (m, 3H), 7.07–6.98 (m, 3H), 6.81–6.78 (m, 1H), 4.16–4.14 (m, 1H), 4.10–3.98 (m, 2H), 3.82–3.76 (m, 4H), 3.28–3.24 (m, 4H), 3.08 (s, 3H), 2.98–2.93 (m, 2H), 1.32 (s, 9H); LCMS (ES, m/z) calculated for C_30_H_35_N_6_O_3_^+^ [(M + H)^+^] 527.3, observed 527.2

##### Synthesis of *tert*-Butyl (R)-3-(4-(1-cyclopropyl-[1,2,4] triazolo[4,3-a]quinolin-7-yl)piperazine-1-carbonyl)-3,4-dihydroisoquinoline-2(1H)-carboxylate (8t)

Intermediate **6c** was treated with commercial compound **7f.** According to general procedure **8a-8u**. Compound **8t** was obtained as 0.1 g (yield: 66%) of a yellow solid.

^*1*^*H NMR (400 MHz, DMSO-d*_*6*_*)*: δ ppm 8.70 (d, *J* = 9.2 Hz, 1H), 7.66 (d, *J* = 9.6 Hz, 1H), 7.59–7.53 (m, 2H), 7.49–7.46 (m, 1H), 7.21–7.16 (m, 4H), 5.01–4.98 (m, 1H), 4.71–4.68 (m, 1H), 4.32–4.28 (m, 1H), 3.89–3.42 (m, 5H), 3.28–3.18 (m, 4H), 2.98–2.94 (m, 1H), 2.63–2.61 (m, 1H), 1.45 (s, 9H), 1.35–1.33 (m, 2H), 1.16–1.14 (m, 2H) LCMS (ES, m/z) calculated for C_32_H_37_N_6_O_3_^+^ [(M + H)^+^] 553.2, observed 553.2.

##### Synthesis of *tert*-Butyl (R)-3-(4-(1-(trifluoromethyl)-[1,2,4] triazolo[4,3-a]quinolin-7-yl)piperazine-1-carbonyl)-3,4-dihydroisoquinoline-2(1H)-carboxylate (8u)

Intermediate **6d** was treated with commercial compound **7f.** according to general procedure **8a-8u**. Compound **8u** was obtained as 90 g (yield: 60%) of a yellow solid.

^*1*^*H NMR (400 MHz, DMSO-d*_*6*_*)*: δ ppm 8.01–7.94 (m, 2H), 7.85 (d, *J* = 9.6 Hz, 1H), 7.59–7.56 (m, 2H), 7.28–7.26 (m, 4H), 4.70–4.64 (m, 1H), 3.73–3.54 (m, 5H), 3.28–3.16 (m, 3H), 3.02–2. 82 (m, 4H), 1.33 (s, 9H); LCMS (ES, m/z) calculated for C_30_H_32_F_3_N_6_O_3_^+^ [(M + H)^+^] 581.2, observed 581.

#### General procedure for the synthesis of final compounds 9a-9u

Compounds **8a** (1 equiv.) in 1,4-dioxane (10 volumes) at 0 °C was added 4 M HCl in 1,4-dioxane (4 equiv.). TLC was employed to monitor the reaction’s progress. After reaction complies, the solvent was evaporated under reduced pressure. The precipitate obtained was given diethyl ether washes and dried under high vacuum to produce the final compounds **9a** as HCl salt. Intermediate **8b-8u** was followed with similar procedure to obtain the desired products **9b-9u.**

##### (*R*)-1-(4-([1,2,4]Triazolo[4,3-a]quinolin-7-yl)piperazin-1-yl)-2-amino-3-phenylpropan-1-one hydrochloride (9a)

Yield: 60 mg (85%); Yellow solid, Melting point: 182–184 °C; ^1^H NMR (CD_3_OD, 400 MHz) δ 10.06 (s, 1H), 8.41 (d, *J* = 9.2 Hz, 1H), 8.29 (d, *J* = 9.6 Hz, 1H), 7.83 (d, *J* = 9.6 Hz, 1H), 7.66 (dd, *J* = 9.2 Hz, 2.4 Hz, 1H), 7.50 (d, *J* = 2.6 Hz, 1H), 7.39–7.28 (m, 5H), 4.78–4.76 (m, 1H), 3.79–3.76 (m, 2H), 3.58–3.54 (m, 1H), 3.42–3.39 (m, 2H), 3.28–3.14 (m 5H), 2.71–2.68 (m, 1H); ^13^C NMR (DMSO*-d*_*6*_*,* 101 MHz) δ 166.51, 149.04, 145.32, 136.15, 134.68, 133.80, 133.25, 131.58, 129.01, 127.80, 124.41, 123.11, 120.19, 117.63, 113.06, 111.99, 53.22, 47.92, 47.76, 47.71, 44.70, 33.97; HPLC: 97.52%; ESI–MS calculated [m/z] for C_23_H_25_N_6_O^+^ [(M + H)^+^] is 401.2, and observed 401.2; HRMS calculated [m/z] for C_23_H_25_N_6_O^+^ [(M + H)^+^] is 401.2090, and observed 401.2083.

##### (*R*)-1-(4-([1,2,4]Triazolo[4,3-a]quinolin-7-yl)piperazin-1-yl)-2-amino-3-(4-methoxy phenyl)propan-1-one hydrochloride (9b)

Yield: 60 mg (85%); Yellow solid, Melting point: 182–184 °C; ^1^H NMR (CD_3_OD, 400 MHz) δ10.13 (s, 1H), 8.46 (d, *J* = 9.2 Hz, 1H), 8.39 (d, *J* = 9.6 Hz, 1H), 7.87 (d, *J* = 9.6 Hz, 1H), 7.71 (dd, *J* = 9.2, 2.4 Hz, 1H), 7.53 (d, *J* = 2.4 Hz, 1H), 7.26 (d, *J* = 8.4 Hz, 2H), 6.93 (d, *J* = 8.4 Hz, 2H), 4.74–4.72 (m, 1H), 3.85–3.72 (m, 2H), 3.69 (s, 3H), 3.60–3.43 (m, 2H), 3.28–3.24 (m, 3H), 3.18–3.04 (m, 2H), 2.64–2.58 (m, 1H); ^13^C NMR (DMSO*-d*_*6*_*,* 101 MHz) δ 166.90, 158.61, 149.21, 136.16, 130.85, 126.31, 117.60, 114.08, 113.01, 55.02, 49.75, 47.69, 44.50, 36.26; HPLC: 97.15%; ESI–MS calculated [m/z] for C_24_H_27_N_6_O_2_^+^ [(M + H)^+^] is 431.2, and observed 431.2; HRMS calculated [m/z] for C_24_H_27_N_6_O_2_^+^ [(M + H)^+^] is 431.2195, and observed 431.2189.

##### (*R*)-1-(4-([1,2,4]Triazolo[4,3-a]quinolin-7-yl)piperazin-1-yl)-2-amino-3-(2,4-dichloro phenyl)propan-1-one hydrochloride (9c)

Yield: 54 mg (71%); Yellow solid, Melting point: 162–164 °C; ^1^H NMR (CD_3_OD, 400 MHz) δ 10.03 (s, 1H), 8.41 (d, *J* = 9.6 Hz, 1H), 8.25 (d, *J* = 9.6 Hz, 1H), 7.81 (d, *J* = 9.6 Hz, 1H), 7.68 (dd, *J* = 9.2, 2.8 Hz, 1H), 7.61 (d, *J* = 1.6 Hz, 1H), 7.52 (d, *J* = 2.8 Hz, 1H), 7.42–7.37 (m, 2H), 4.85–4.81 (m, 1H), 3.81–3.75 (m, 2H), 3.65–3.59 (m, 1H), 3.45–3.38 (m, 2H), 3.28–3.21 (m, 4H), 2.77–2.72 (m, 1H); ^13^C NMR (DMSO*-d*_*6*_*,* 101 MHz) δ 166.51, 149.04, 145.32, 136.15, 134.68, 133.80, 133.25, 131.58, 129.01, 127.80, 124.41, 123.11, 120.19, 117.63, 113.06, 111.99, 53.22, 47.92, 47.76, 47.71, 44.70, 33.97; HPLC: 99.38%; HRMS calculated [m/z] for C_23_H_23_Cl_2_N_6_O^+^ [(M + H)^+^] is 469.1310, and observed 469.1307.

##### (*R*)-1-(4-([1,2,4]Triazolo[4,3-a]quinolin-7-yl)piperazin-1-yl)-2-amino-3-(3-chlorophenyl) propan-1-one hydrochloride (9d)

Yield: 65 mg (93%); Yellow solid, Melting point: 173–175 °C; ^1^H NMR (CD_3_OD, 400 MHz) δ 10.07 (s, 1H), 8.43 (d, *J* = 9.2 Hz, 1H), 8.30 (d, *J* = 9.6 Hz, 1H), 7.83 (d, *J* = 9.6 Hz, 1H), 7.68 (dd, *J* = 9.6, 2.4 Hz, 1H), 7.54 (d, *J* = 2.8 Hz, 1H), 7.42–7.38 (m, 2H), 7.33–7.30 (m, 2H), 4.83–4.81 (m, 1H), 3.88–3.78 (m, 2H), 3.68–3.62 (m, 1H), 3.48–3.38 (m, 2H), 3.28–3.16 (m, 4H), 2.78–2.72 (m, 1H); ^13^C NMR (DMSO*-d*_*6*_*,* 101 MHz) δ 166.47, 148.90, 136.86, 135.96, 132.94, 130.24, 129.40, 128.41, 127.16, 124.24, 120.00, 117.47, 112.79, 56.58, 49.28, 47.70, 47.41, 44.39, 36.09; HPLC: 98.65%; ESI–MS calculated [m/z] for C_23_H_24_ClN_6_O^+^ [(M + H)^+^] is 435.17, and observed 435.1; HRMS calculated [m/z] for C_23_H_24_ClN_6_O^+^ [(M + H)^+^] is 435.1700, and observed 435.1692.

##### (*R*)-2-Amino-1-(4-(1-methyl-[1,2,4]triazolo[4,3-a]quinolin-7-yl)piperazin-1-yl)-3-phenyl propan-1-one hydrochloride (9e)

Yield: 57 mg (81%); Yellow solid, Melting point: 165–167 °C; ^1^H NMR (CD_3_OD, 400 MHz) δ 8.50 (d, *J* = 9.2 Hz, 1H), 8.30 (d, *J* = 9.6 Hz, 1H), 7.78 (d, *J* = 9.2 Hz, 1H), 7.64 (d, *J* = 8.8 Hz, 1H), 7.54 (s, 1H), 7.42–7.28 (m, 5H), 4.83–4.81 (m, 1H), 3.72–3.68 (m, 2H), 3.60–3.57 (m, 1H), 3.48–3.42 (m, 1H), 3.28–3.26 (m, 1H), 3.25 (s, 3H), 3.23–3.08 (m, 4H), 2.72–2.68 (m, 1H); ^13^C NMR (DMSO*-d*_*6*_*,* 101 MHz) δ 166.86, 148.70, 146.65, 134.61, 129.73, 128.62, 127.32, 125.55, 119.23, 117.97, 113.41, 111.62, 49.58, 47.21, 44.48, 37.09, 14.83; HPLC: 98.16%; ESI–MS calculated [m/z] for C_24_H_27_N_6_O^+^ [(M + H)^+^] is 415.2, and observed 415.1; HRMS calculated [m/z] for C_24_H_27_N_6_O^+^ [(M + H)^+^] is 415.2246, and observed 415.2236.

##### (*R*)-2-Amino-3-(4-hydroxyphenyl)-1-(4-(1-methyl-[1,2,4]triazolo[4,3-a]quinolin-7-yl) piperazin-1-yl)propan-1-one hydrochloride (9f)

Yield: 60 mg (86%); Yellow solid, Melting point: 166–168 °C; ^1^H NMR (CD_3_OD, 400 MHz) δ 8.50 (d, *J* = 9.6 Hz, 1H), 8.30 (d, *J* = 9.6 Hz, 1H), 7.78 (d, *J* = 9.2 Hz, 1H), 7.65 (dd, *J* = 9.4, 2.6 Hz, 1H), 7.54 (d, *J* = 2.8 Hz, 1H), 7.16 (d, *J* = 8.4 Hz, 2H), 6.79 (d, *J* = 8.4 Hz, 2H), 4.72–4.68 (m, 1H), 3.87–3.82 (m, 1H), 3.77–3.68 (m, 1H), 3.58–3.53 (m, 1H), 3.48–3.42 (m, 1H), 3.29–3.27 (m, 1H), 3.26 (s, 3H), 3.25–2.21 (m, 2H), 3.18–3.12 (m, 1H), 3.08–3.02 (m, 1H), 2.72–2.68 (m, 1H); ^13^C NMR (DMSO*-d*_*6*_*,* 101 MHz) δ 167.04, 156.82, 148.89, 146.77, 145.69, 135.42, 130.62, 125.64, 124.39, 123.97, 119.63, 118.14, 115.40, 113.46, 110.97, 53.66, 49.84, 47.33, 44.45, 14.80; HPLC: 99.27%; ESI–MS calculated [m/z] for C_24_H_27_N_6_O_2_^+^ [(M + H)^+^] is 431.2, and observed 431.1; HRMS calculated [m/z] for C_24_H_27_N_6_O_2_^+^ [(M + H)^+^] is 431.2195, and observed 431.2190.

##### (*R*)-2-Amino-3-(4-methoxyphenyl)-1-(4-(1-methyl-[1,2,4]triazolo[4,3-a]quinolin-7-yl) piperazin-1-yl)propan-1-one hydrochloride (9g)

Yield: 55 mg (78%); Yellow solid, Melting point: 149–151 °C; ^1^H NMR (CD_3_OD, 400 MHz) δ 8.50 (d, *J* = 9.6 Hz, 1H), 8.27 (d, *J* = 9.6 Hz, 1H), 7.78 (d, *J* = 9.2 Hz, 1H), 7.65 (dd, *J* = 9.4, 2.6 Hz, 1H), 7.52 (d, *J* = 3.2 Hz, 1H), 7.25 (dd, *J* = 6.8, 2.0 Hz, 2H), 6.93 (dd, *J* = 6.6, 2.2 Hz, 2H), 4.73–4.68 (m, 1H), 3.89–3.84 (m, 1H), 3.77–3.72 (m, 1H), 3.68 (s, 3H), 3.58–3.53 (m, 1H), 3.48–3.42 (m, 1H), 3.29–3.27 (m, 1H), 3.26 (s, 3H), 3.18–3.08 (m, 4H), 2.65–2.60 (m, 1H); ^13^C NMR (DMSO*-d*_*6*_*,* 101 MHz) δ 166.88, 158.60, 148.71, 130.84, 126.28, 125.49, 119.20, 117.79, 114.07, 113.47, 112.19, 55.02, 53.29, 49.75, 47.48, 36.24, 14.89; HPLC: 99.13%; ESI–MS calculated [m/z] for C_25_H_29_N_6_O_2_^+^ [(M + H)^+^] is 445.2, and observed 445.1; HRMS calculated [m/z] for C_25_H_29_N_6_O_2_^+^ [(M + H)^+^] is 445.2352, and observed 445.2344.

##### (*R*)-2-Amino-3-(2,4-dichlorophenyl)-1-(4-(1-methyl-[1,2,4]triazolo[4,3-a]quinolin-7-yl) piperazin-1-yl)propan-1-one hydrochloride (9h)

Yield: 62 mg (88%); Yellow solid, Melting point: 158–160 °C; ^1^H NMR (CD_3_OD, 400 MHz) δ 8.52 (d, *J* = 9.2 Hz, 1H), 8.32 (d, *J* = 9.2 Hz, 1H), 7.79 (d, *J* = 9.6 Hz, 1H), 7.68 (dd, *J* = 9.4, 2.0 Hz, 1H), 7.61 (d, *J* = 1.2 Hz, 1H), 7.57 (d, *J* = 2.4 Hz, 1H), 7.40–7.39 (m, 2H), 4.84–4.81 (m, 1H), 3.81–3.75 (m, 2H), 3.65–3.59 (m, 1H), 3.48–3.38 (m, 3H), 3.29–3.28 (m, 2H), 3.27(s, 3H), 3.26–3.24 (m, 1H), 2.80–2.72 (m, 1H); ^13^C NMR (DMSO*-d*_*6*_*,* 101 MHz) δ 167.04, 156.82, 148.89, 146.77, 145.69, 135.42, 130.62, 125.64, 124.39, 123.97, 119.63, 118.14, 115.40, 113.46, 110.97, 49.84, 47.33, 44.45, 36.39, 14.80; HPLC: 99.52%; ESI–MS calculated [m/z] for C_24_H_25_Cl_2_N_6_O^+^ [(M + H)^+^] is 483.1, and observed 483.0; HRMS calculated [m/z] for C_24_H_25_Cl_2_N_6_O^+^ [(M + H)^+^] is 483.1467, and observed 483.1460.

##### (*R*)-2-Amino-3-(3-chlorophenyl)-1-(4-(1-methyl-[1,2,4]triazolo[4,3-a]quinolin-7-yl) piperazin-1-yl)propan-1-one hydrochloride (9i)

Yield: 54 mg (77%); Yellow solid, Melting point: 154–156 °C; ^1^H NMR (CD_3_OD, 400 MHz) δ 8.52 (d, *J* = 9.2 Hz, 1H), 8.32 (d, *J* = 9.6 Hz, 1H), 7.79 (d, *J* = 9.6 Hz, 1H), 7.66 (dd, *J* = 9.6, 2.8 Hz, 1H), 7.58 (d, *J* = 2.8 Hz, 1H), 7.42–7.38 (m, 2H), 7.33–7.30 (m, 2H), 4.82–4.77 (m, 1H), 3.88–3.72 (m, 2H), 3.68–3.62 (m, 1H), 3.48–3.40 (m, 1H), 3.38–3.36 (m, 1H), 3.28–3.27 (m, 1H), 3.26(s, 3H), 3.25–3.21 (m, 3H), 2.78–2.74 (m, 1H); ^13^C NMR (DMSO*-d*_*6*_*,* 101 MHz) δ 166.70, 148.85, 146.78, 145.92, 137.08, 135.03, 133.14, 130.44, 129.60, 128.62, 127.35, 125.63, 124.08, 119.43, 118.11, 113.44, 111.28, 49.47, 47.55, 47.29, 44.55, 36.30, 14.82; HPLC: 98.39%; ESI–MS calculated [m/z] for C_24_H_26_ClN_6_O [(M + H)^+^] is 449.1, and observed 449.1; HRMS calculated [m/z] for C_24_H_26_ClN_6_O [(M + H)^+^] is 449.1857, and observed 449.1851.

##### (*R*)-2-Amino-1-(4-(1-cyclopropyl-[1,2,4]triazolo[4,3-a]quinolin-7-yl)piperazin-1-yl)-3-phenylpropan-1-one hydrochloride (9j)

Yield: 62 mg (88%); Yellow solid, Melting point: 178–180 °C; ^1^H NMR (CD_3_OD, 400 MHz) δ 8.93 (d, *J* = 9.2 Hz, 1H), 8.18 (d, *J* = 9.6 Hz, 1H), 7.72 (d, *J* = 9.6 Hz, 1H), 7.61 (dd, *J* = 9.6, *J* = 2.8 Hz, 1H), 7.50 (d, *J* = 2.8 Hz, 1H), 7.42–7.30 (m, 5H), 4.79–4.77 (m, 1H), 3.78–3.77 (m, 2H), 3.58–3.53 (m, 1H), 3.46–3.41 (m, 1H), 3.29–3.21 (m, 3H), 3.18–3.11 (m, 2H), 2.78–2.68 (m, 2H), 1.49–1.46 (m, 2H), 1.37–1.34 (m, 2H); ^13^C NMR (DMSO*-d*_*6*_, 101 MHz) δ 166.86, 148.70, 146.65, 134.61, 129.73, 128.62, 127.32, 125.55, 119.23, 117.97, 113.41, 111.62, 49.67, 47.31, 44.41, 36.18, 8.71, 7.14; HPLC: 99.0%; HRMS calculated [m/z] for C_26_H_29_N_6_O^+^ [(M + H)^+^] is 441.2403, and observed 441.2397.

##### *R*)-2-Amino-1-(4-(1-cyclopropyl-[1,2,4]triazolo[4,3-a]quinolin-7-yl)piperazin-1-yl)-3-(4-hydroxyphenyl)propan-1-one hydrochloride (9k)

Yield: 58 mg (82%); Yellow solid, Melting point: 164–166 °C; ^1^H NMR (400 MHz, DMSO-*d*_6_) δ 9.41 (brs, 1H), 8.73 (d, *J* = 9.2 Hz, 1H), 8.29–8.27 (m, 2H), 7.89 (d, *J* = 9.6 Hz, 1H), 7.71 (d, *J* = 9.6 Hz, 1H), 7.52–7.50 (m, 2H), 7.05 (d, *J* = 8.4 Hz, 2H), 6.73 (d, *J* = 8.4 Hz, 2H), 4.75–4.71 (m, 1H), 3.64–3.64 (m, 2H), 3.52–3.31 (m, 1H), 3.28–3.14 (m, 4H), 3.01–3.43 (m, 1H), 2.88–2.86 (m, 1H), 2.68–2.62 (m, 1H), 2.34–2.32 (m, 1H), 1.32–1.28 (m, 2H), 1.20–1.88 (m, 2H); ^13^C NMR (DMSO*-d*_*6*_*,* 101 MHz) δ 166.86, 154.97, 152.72, 150.23, 148.47, 134.58, 129.74, 128.63, 127.34, 118.93, 118.03, 114.57, 113.43, 49.61, 47.51, 47.37, 44.52, 37.10, 8.88, 7.02; HPLC: 91.43%; ESI–MS calculated [m/z] for C_26_H_29_N_6_O_2_^+^ [(M + H)^+^] is 457.2, and observed 457.2.

##### (*R*)-2-Amino-1-(4-(1-cyclopropyl-[1,2,4]triazolo[4,3-a]quinolin-7-yl)piperazin-1-yl)-3-(4-methoxyphenyl)propan-1-one hydrochloride (9l)

Yield: 54 mg (77%); Yellow solid, Melting point: 176–178 °C; ^1^H NMR (CD_3_OD, 400 MHz) δ 8.97 (d, *J* = 9.2 Hz, 1H), 8.29 (d, *J* = 9.6 Hz, 1H), 7.77 (d, *J* = 9.6 Hz, 1H), 7.66 (dd, *J* = 9.2, 2.0 Hz, 1H), 7.53 (d, *J* = 2.4 Hz, 1H), 7.26 (d, *J* = 8.4 Hz, 2H), 6.94 (d, *J* = 8.6 Hz, 2H), 4.75–4.71 (m, 1H), 3.88–3.85 (m, 1H), 3.75–3.71 (m, 1H), 3.69 (s, 3H), 3.59–3.56 (m, 1H), 3.48–3.43 (m, 1H), 3.29–3.08 (m, 5H), 2.77–2.75 (m, 1H), 2.62–2.58 (m, 1H), 1.52–1.49 (m, 2H), 1.40–1.37 (m, 2H); ^13^C NMR (DMSO*-d*_*6*_*,* 101 MHz) δ 166.85, 158.51, 150.28, 148.85, 135.25, 130.80, 126.31, 125.62, 124.10, 119.56, 118.30, 114.01, 113.31, 110.93, 55.02, 49.67, 47.31, 44.41, 36.18, 8.72, 7.14; HPLC: 98.79%; ESI–MS calculated [m/z] for C_27_H_31_N_6_O_2_^+^ [(M + H)^+^] is 471.2, and observed 471.2; HRMS calculated [m/z] for C_27_H_31_N_6_O_2_^+^ [(M + H)^+^] is 471.2508, and observed 471.2501.

##### (*R*)-2-Amino-1-(4-(1-cyclopropyl-[1,2,4]triazolo[4,3-a]quinolin-7-yl)piperazin-1-yl)-3-(2,4-dichlorophenyl)propan-1-one hydrochloride (9m)

Yield: 64 mg (91%); Yellow solid, Melting point: 193–195 °C; ^1^H NMR (CD_3_OD, 400 MHz) δ 8.92 (d, *J* = 9.6 Hz, 1H), 8.16 (d, *J* = 9.6 Hz, 1H), 7.72–7.69 (m, 1H), 7.64–7.59 (m, 2H), 7.51 (d, *J* = 2.4 Hz, 1H), 7.43–7.37 (m, 2H), 4.82–4.80 (m, 1H), 3.82–3.78 (m, 2H), 3.71–3.68 (m, 2H), 3.48–3.43 (m, 3H), 3.29–3.22 (m, 2H), 2.77–2.72 (m, 2H), 1.51–1.45 (m, 2H), 1.36–1.32 (m, 2H); ^13^C NMR (DMSO*-d*_*6*_*,* 101 MHz) δ 166.51, 150.30, 148.69, 146.20, 134.66, 133.79, 133.22, 131.57, 128.99, 127.78, 125.63, 124.36, 119.43, 118.24, 113.40, 111.55, 47.92, 47.45, 44.63, 33.95, 8.78, 7.11; HPLC: 94.66%; ESI–MS calculated [m/z] for C_26_H_27_Cl_2_N_6_O^+^ [(M + H)^+^] is 509.1, and observed 509.1; HRMS calculated [m/z] for C_26_H_27_Cl_2_N_6_O^+^ [(M + H)^+^] is 509.1623, and observed 509.1616.

##### (*R*)-2-Amino-3-(3-chlorophenyl)-1-(4-(1-cyclopropyl-[1,2,4]triazolo[4,3-a]quinolin-7-yl)piperazin-1-yl)propan-1-one hydrochloride (9n)

Yield: 57 mg (81%); Yellow solid, Melting point: 158–160 °C; ^1^H NMR (CD_3_OD, 400 MHz) δ 8.92 (d, *J* = 9.2 Hz, 1H), 8.08 (d, *J* = 9.6 Hz, 1H), 7.68 (d, *J* = 9.6 Hz, 1H), 7.59 (dd,* J* = 9.6, 2.4 Hz, 1H), 7.50 (d, *J* = 2.4 Hz, 1H), 7.43–7.38 (m, 2H), 7.34–7.29 (m, 2H), 4.82–4.79 (m, 1H), 3.86–3.74 (m, 2H), 3.64–3.61 (m, 1H), 3.48–3.42 (m, 1H), 3.29–3.17 (m, 5H), 2.71–2.65 (m, 2H), 1.47–1.42 (m, 2H), 1.35–1.31 (m, 2H); ^13^C NMR (DMSO*-d*_*6*_*,* 101 MHz) δ 166.67, 150.23, 148.47, 131.40, 130.43, 129.60, 128.61, 127.35, 125.50, 118.94, 118.02, 113.49, 49.47, 47.73, 47.49, 44.59, 36.28, 8.90, 7.02; HPLC: 98.24%; ESI–MS calculated [m/z] for C_26_H_28_ClN_6_O^+^ [(M + H)^+^] is 475.2, and observed 475.1; HRMS calculated [m/z] for C_26_H_28_ClN_6_O^+^ [(M + H)^+^] is 475.2013, and observed 475.2007.

##### (*R*)-2-Amino-3-phenyl-1-(4-(1-(trifluoromethyl)-[1,2,4]triazolo[4,3-a]quinolin-7-yl)piperazin-1-yl)propan-1-one hydrochloride (9o)

Yield: 56 mg (80%); Yellow solid, Melting point: 167–169 °C; ^1^H NMR (CD_3_OD, 400 MHz) δ 8.15 (d, *J* = 9.6 Hz, 1H), 7.97 (d, *J* = 9.6 Hz, 1H), 7.75 (d, *J* = 9.6 Hz, 1H), 7.50 (dd, *J* = 9.6, 2.8 Hz, 1H), 7.43–7.29 (m, 6H), 4.80–4.75 (m, 1H), 3.79–3.76 (m, 2H), 3.57–3.53 (m, 1H), 3.42–3.39 (m, 1H), 3.28–3.12 (m, 5H), 2.71–2.64 (m, 1H); ^13^C NMR (DMSO*-d*_*6*_*,* 101 MHz) δ 166.89, 151.05, 148.71, 134.56, 132.50, 129.73, 128.63, 127.36, 125.81, 122.61, 119.06, 117.35, 114.08, 113.46, 49.63, 47.21, 47.02, 44.43, 37.09; HPLC: 95.72%; ESI–MS calculated [m/z] for C_24_H_24_F_3_N_6_O^+^ [(M + H)^+^] is 469.2, and observed 469.2; HRMS calculated [m/z] for C_24_H_24_F_3_N_6_O^+^ [(M + H)^+^] is 469.1964, and observed 469.1957.

##### (***R***)-2-Amino-3-(4-methoxyphenyl)-1-(4-(1-(trifluoromethyl)-[1,2,4]triazolo[4,3-a]quinolin-7-yl)piperazin-1-yl)propan-1-one hydrochloride (9p)

Yield: 61 mg (87%); Yellow solid, Melting point: 178–180 °C; ^1^H NMR (CD_3_OD, 400 MHz) δ 8.16 (d, *J* = 9.6 Hz, 1H), 7.94 (d, *J* = 9.6 Hz, 1H), 7.75 (d, *J* = 9.2 Hz, 1H), 7.50 (dd, *J* = 9.6, 3.0 Hz, 1H), 7.42 (d, *J* = 2.8 Hz, 1H), 7.24 (d, *J* = 9.4 Hz, 2H), 6.94 (d, *J* = 9.4 Hz, 2H), 4.72–4.68 (m, 1H), 3.87–3.85 (m, 1H), 3.74–3.72 (m, 1H), 3.67 (s, 3H), 3.57–3.50 (m, 1H), 3.45–3.40 (m, 1H), 3.28–3.18 (m, 3H), 3.16–3.08 (m, 2H), 2.58–2.56 (m, 1H); ^13^C NMR (DMSO*-d*_*6*_*,* 101 MHz) δ 166.88, 158.56, 151.03, 148.82, 132.48, 130.82, 126.25, 125.76, 122.62, 119.13, 117.28, 114.04, 113.50, 54.97, 49.73, 47.18, 44.37, 36.20; HPLC: 97.26%; ESI–MS calculated [m/z] for C_25_H_26_F_3_N_6_O_2_^+^ [(M + H)^+^] is 499.2, and observed 499.2; HRMS calculated [m/z] for C_25_H_26_F_3_N_6_O_2_^+^ [(M + H)^+^] is 499.2069, and observed 499.2064.

##### (*R*)-2-Amino-3-(2,4-dichlorophenyl)-1-(4-(1-(trifluoromethyl)-[1,2,4]triazolo[4,3-a]quinolin-7-yl)piperazin-1-yl)propan-1-one hydrochloride (9q)

Yield: 53 mg (75%); Yellow solid, Melting point: 162–164 °C; ^1^H NMR (CD_3_OD, 400 MHz) δ 8.17 (d, *J* = 9.2 Hz, 1H), 7.94 (d, *J* = 9.6 Hz, 1H), 7.75 (d, *J* = 9.2 Hz, 1H), 7.63 (d, *J* = 1.6 Hz, 1H), 7.53 (dd, *J* = 9.6, 2.8 Hz, 1H), 7.46 (d, *J* = 2.8 Hz, 1H), 7.39–7.38 (m, 2H), 4.82–4.79 (m, 1H), 3.81–3.78 (m, 2H), 3.61–3.57 (m, 1H), 3.48–3.42 (m, 2H), 3.28–3.21 (m, 2H), 3.16–3.08 (m, 2H), 2.77–2.73 (m, 1H); ^13^C NMR (DMSO*-d*_*6*_*,* 101 MHz) δ 166.53, 151.05, 148.73, 134.66, 133.76, 133.25, 132.48, 131.48, 129.01, 127.78, 125.81, 122.69, 119.19, 114.11, 113.55, 47.96, 47.27, 44.53, 33.97; HPLC: 97.26%; ESI–MS calculated [m/z] for C_24_H_22_Cl_2_F_3_N_6_O^+^ [(M + H)^+^] is 537.1, and observed 537.2; HRMS calculated [m/z] for C_24_H_22_Cl_2_F_3_N_6_O^+^ [(M + H)^+^] is 537.1184, and observed 537.1179.

##### (*R*)-2-Amino-3-(3-chlorophenyl)-1-(4-(1-(trifluoromethyl)-[1,2,4]triazolo[4,3-a]quinolin-7-yl)piperazin-1-yl)propan-1-one hydrochloride (9r)

Yield: 65 mg (93%); Yellow solid, Melting point: 163–165 °C; ^1^H NMR (CD_3_OD, 400 MHz) δ 8.17 (d, *J* = 9.2 Hz, 1H), 7.94 (d, *J* = 9.6 Hz, 1H), 7.75 (d, *J* = 9.6 Hz, 1H), 7.53 (dd, *J* = 9.4, 3.0 Hz, 1H), 7.46 (d, *J* = 2.8 Hz, 1H), 7.43–7.28 (m, 4H), 4.79–4.75 (m, 1H), 3.86–3.78 (m, 2H), 3.61–3.57 (m, 1H), 3.44–3.42 (m, 1H), 3.28–3.21 (m, 3H), 3.19–3.16 (m, 2H), 2.78–2.74 (m, 1H); ^13^C NMR (DMSO*-d*_*6*_*,* 101 MHz) δ 166.69, 151.06, 136.93, 133.16, 130.46, 129.59, 128.61, 127.40, 119.15, 114.13, 113.52, 49.57, 47.37, 44.45, 36.27; HPLC: 96.35%; ESI–MS calculated [m/z] for C_24_H_23_ClF_3_N_6_O^+^ [(M + H)^+^] is 503.1, and observed 503.2; HRMS calculated [m/z] for C_24_H_23_ClF_3_N_6_O^+^ [(M + H)^+^] is 503.1574, and observed 503.1568.

##### (*R*)-(4-(1-Methyl-[1,2,4]triazolo[4,3-a]quinolin-7-yl)piperazin-1-yl)(1,2,3,4-tetrahydroisoquinolin-3-yl) methanone hydrochloride (9s)

Yield: 60 mg (86%); Yellow solid, Melting point: 175–177 °C; ^1^H NMR (CD_3_OD, 400 MHz) δ 8.28 (d, *J* = 10.0 Hz, 1H), 7.68 (d, *J* = 9.6 Hz, 1H), 7.51–7.43 (m, 3H), 7.17–7.15 (m, 3H), 7.1–7.09 (m, 1H), 4.20–4.17 (m, 1H), 4.12–4.10 (m, 2H), 3.91–3.87 (m, 4H), 3.43–3.41 (m, 4H), 3.11 (s, 3H), 2.98–2.93 (m, 2H); ^13^C NMR (DMSO*-d*_*6*_*,* 101 MHz) δ 166.81, 148.87, 146.60, 130.96, 128.73, 128.55, 127.46, 126.88, 126.55, 124.35, 119.32, 117.90, 113.57, 112.08, 72.57, 67.97, 53.27, 52.10, 47.63, 44.50, 43.97, 28.80, 16.70; HPLC: 95.16%; ESI–MS calculated [m/z] for C_25_H_27_N_6_O^+^ [(M + H)^+^] is 427.2, and observed 427.1; HRMS calculated [m/z] for C_25_H_27_N_6_O^+^ [(M + H)^+^] is 427.2246, and observed 427.2240.

##### (*R*)-(4-(1-Cyclopropyl-[1,2,4]triazolo[4,3-a]quinolin-7-yl)piperazin-1-yl)(1,2,3,4-tetrahydroisoquinolin-3-yl)methanone hydrochloride (9t)

Yield: 52 mg (74%); Yellow solid, Melting point: 162–164 °C; ^1^H NMR (CD_3_OD, 400 MHz) δ 9.01 (d, *J* = 9.2 Hz, 1H), 8.33 (d, *J* = 9.6 Hz, 1H), 7.79–7.77 (m, 2H), 7.69 (d, *J* = 2.4 Hz, 1H), 7.35–7.29 (m, 4H), 4.50–4.46 (m, 2H), 4.02–3.99 (m, 1H), 3.89–3.84 (m, 3H), 3.61–3.44 (m, 6H), 3.23–3.16 (m, 1H), 2.80–2.76 (m, 1H), 1.54–1.50 (m, 2H), 1.41–1.36 (m, 2H); ^13^C NMR (DMSO*-d*_*6*_*,* 101 MHz) δ 166.82, 150.31, 148.87, 146.08, 134.65, 130.96, 128.71, 128.56, 127.42, 126.85, 126.54, 125.69, 124.29, 119.53, 118.31, 113.47, 52.02, 48.12, 47.57, 44.49, 43.93, 28.80, 8.78, 7.13; HPLC: 97.74%; ESI–MS calculated [m/z] for C_27_H_29_N_6_O^+^ [(M + H)^+^] is 453.2, and observed 453.1; HRMS calculated [m/z] for C_27_H_29_N_6_O^+^ [(M + H)^+^] is 453.2403, and observed 453.2396.

##### (*R*)-(1,2,3,4-Tetrahydroisoquinolin-3-yl)(4-(1-(trifluoromethyl)-[1,2,4]triazolo[4,3-a]quinolin-7-yl)piperazin-1-yl)methanone hydrochloride (9u)

Yield: 48 mg (68%); Yellow solid, Melting point: 174–176 °C; ^1^H NMR (CD_3_OD, 400 MHz) δ 8.19 (d, *J* = 9.2 Hz, 1H), 8.03 (d, *J* = 9.6 Hz, 1H), 7.78 (d, *J* = 9.6 Hz, 1H), 7.64 (dd, *J* = 9.4, 2.4 Hz, 1H), 7.59 (d, *J* = 2.4 Hz, 1H), 7.35–7.28 (m, 4H), 4.50–4.48 (m, 2H), 4.02–3.98 (m, 1H), 3.89–3.84 (m, 3H), 3.68–3.66 (m, 1H), 3.58–3.42 (m, 5H), 3.23–3.18 (m, 1H); ^13^C NMR (DMSO*-d*_*6*_*,* 101 MHz) δ 166.82, 151.08, 148.90, 132.54, 130.96, 128.74, 128.53, 127.46, 126.88, 126.54, 125.86, 122.65, 119.27, 117.40, 116.87, 114.10, 113.59, 52.12, 47.92, 47.35, 43.98, 28.79; HPLC: 96.18%; ESI–MS calculated [m/z] for C_25_H_24_F_3_N_6_O^+^ [(M + H)^+^] is 481.2, and observed 481.2; HRMS calculated [m/z] for C_25_H_24_F_3_N_6_O^+^ [(M + H)^+^] is 481.1964, and observed 481.1952.

### Biological methods

#### Molecular docking and MM-GBSA calculations

##### Protein preparation and grid generation

To carry out the molecular docking studies, a crystal structure of human HDAC8 (PDB ID: 3SFH) with a resolution of 2.0 Å^45^ was selected and imported into the maestro viewer interface of the Schrödinger software which is freely available to visualize 3D structures of proteins and ligands (Schrödinger, LLC., NY, release 2022-2 suite)^61^. To prepare the protein, protein preparation wizard was executed during which missing residues were added using prime module, and water molecules in the radius of 5.0 Å around the ligand were removed. Further, to prepare the grid for carrying out molecular docking studies, receptor-grid generation wizard was used, and the co-ordinates of the co-crystallized ligand (X = 9.26; Y = 13.73 and Z = − 23.86) were selected to generate a grid box of 10 Å^62^.

##### Ligand preparation

The molecules **9m** and **9r** were sketched in the 2D sketcher of the maestro interface and prepared using the Ligprep module of the Schrödinger software (Schrödinger, 2022-2 suite)^62^. The forcefield used was optimized potential for liquid simulations 2005 (OPLS_2005), and the ionization sate was set to neutralize. The chiralities were determined from the 3D structure. These prepared ligands were used to carry out molecular docking study.

##### Molecular docking

Molecular docking helps to determine the binding pattern of the molecules within the active site of the protein. The ligand docking wizard which uses the glide module of the Schrödinger software (Schrödinger, 2022-2 suite), was used to carry out this analysis^63^. The mode of precision was set to extra precision (XP) and the ligand sampling was flexible to generate several poses and produce the best among them as output. All other parameters were set to default.

##### MMGBSA studies

Molecular Mechanics- Generalized Born surface Area (MM-GBSA) solvation helps to determine the free energy (ΔG) of the ligand by including van der Waals, bonded and electrostatic interactions^56^. The complexes from molecular docking were considered for MM-GBSA studies using prime module of the Schrödinger software. The solvation model used was Vacuum Solvent Generalized Born (VSGB) continuum and the forcefield was OPLS_2005.

#### Molecular dynamics and principal components analysis

Molecular dynamics (MD) helps to determine the behaviour of the ligand protein complex in the biological system, and also helps in observing the stability of the protein–ligand as well as their interactions over the course of time. The complexes from molecular docking were imported into the Desmond module of the Schrödinger software (Schrödinger, 2022-2 suite), the system was setup using transferable intermolecular potential with 3 points (TIP3P) solvent model^64^ and the boundary conditions were set to cubic box with dimension of 10 Å^62^. Salt concentration of 0.15 M NaCl is added to mimic the biological fluid. The complete solvated system is then used to carry out molecular dynamics study for 100 ns with recording interval of 100 pc at temperature of 310 K and 1.012 bar pressure using NTP ensembles. To maintain the constant temperature and pressure Nose–Hoover chain thermostat and Martyna-Tobias-Klein barostat were used respectively^65,66^. For comparative analysis, the protein in complex with Compound **A** was also subjected to MD analysis. The root mean square deviations (RMSD) and fluctuations (RMSF) were used to interpret the stability of the complexes. The trajectories from the molecular dynamics were used to carry out principal component analysis (PCA) that captures the variance of changes in the conformations of the protein–ligand complex during the MD simulation. To perform this, trajectories from MD simulations were used to generate a *.dcd file using visual molecular dynamics (VMD) software, and the same was subjected to Galaxy Europe web-server to perform principal component analysis using a Bio3D package^67,68^. This analysis helps in understanding the behaviour of the complexes during the simulations.

#### Cell culture

The human cancer cell lines HCT-116, MCF7, IMR32, and HEK293 were procured from NCCS, Pune, India. Cells were grown in Dulbecco-modified Eagle’s medium (DMEM) with 10% Heat-inactivated fetal bovine serum (FBS) and 1% penicillin/streptomycin antibiotics in an incubator with 5% CO_2_ at 37 °C.

#### Cell proliferation assay

For cell viability and proliferation, the MTT assay was performed. Cells were seeded at a concentration of 2000 cells/100μL medium/well in a 96-well plate. Once adhered, the cells were treated with various concentrations of representative compounds, along with HDAC inhibitors like SAHA and incubated for 72 h. This was followed by cell viability analysis using MTT according to the manufacturer’s instructions. Briefly, 10 μL of MTT solution was added to each well and incubated for 3 h at 37 °C. The formazan crystals formed were dissolved in 200 μL of solubilization solution (DMSO), and absorbance was recorded at 570 nm. Graphs were plotted according to the absorbance value, and the half-maximal inhibitory concentration (IC_50_) value was determined with the help of GraphPad Prism 8.0.2. (GraphPad Software, San Diego, California USA, www.graphpad.com).

#### Clonogenic assay

IMR32 cells were seeded in a six-well culture plate at a density of 500 cells/well. After the cells adhered completely, they were incubated for 9–12 days in the culture medium with **9h** at concentrations of 5 µM and 10 µM and **9m** at concentrations of 1 µM and 5 µM. The colonies formed were fixed with a 3:1 ratio of methanol and glacial acetic acid for 10 min. The wells were then washed with 1X PBS, and cells were stained with 0.5% v/v crystal violet solution for 10 min. The excess stain was washed off in running water, and images were taken. Colonies were counted visually or using ImageJ software, NIH (Version 1.53 K). The stain taken up by the colonies was dissolved in 10% SDS, and absorbance was taken at 540 nm. Graphs were plotted using GraphPad Prism 8.0.2. (GraphPad Software, San Diego, California USA, www.graphpad.com).

#### Wound healing assay

0.2 × 10^6^ IMR32 cells were seeded in a 12-well tissue culture plate and incubated till a uniform monolayer was formed. Once confluent, the cell layer was scraped in a straight line using a 200 μl pipette tip. After the scratch, a gentle wash was given with 1X PBS to remove detached cells. The cells were incubated with compounds **9h** at concentrations of 5 µM and 10 µM and **9m** at concentrations of 1 µM and 5 µM, and images were taken under a microscope on 4X magnification at 0 h, 12 h, 24 h, 36 h, and 48 h. The percentage of wound closure was calculated to evaluate the of the compounds.

#### Cell apoptosis analysis

The apoptosis assay was performed using an Annexin V–FITC Apoptosis detection kit (Sigma-Aldrich, APOAF) according to the manufacturer’s instructions. 0.1 × 10^6^ cells were seeded in a six-well cell culture plate and allowed to adhere, followed by treatment with DMSO as the control and compound **9h** at concentrations of 5 µM and 10 µM and **9m** at concentrations of 1 µM and 5 µM for 48 h. The cells were trypsinized, pelleted down, and resuspended in the 1X buffer provided in the kit. The cells were stained with Annexin V–FITC/PI and acquired immediately using flow cytometry. The data was analyzed, and graphs were plotted using BD FlowJo version10 software. (https://www.flowjo.com/flowjo/).

#### Cell-cycle analysis

Cell-cycle analysis was carried out using a cell-cycle analysis kit (Sigma-Aldrich, MAK344) according to the protocol given by the manufacturer. 0.2 × 10^6^ IMR32 cells were treated with DMSO or with **9h** at concentrations of 5 µM and 10 µM and **9m** at concentrations of 1 µM and 5 µM and incubated for 48 h. Cells were then trypsinized and pelleted down. The pellets were washed with 1X cell-cycle buffer and fixed in ice-cold 70% ethanol for 1 h at 4 °C. The fixed cells were treated with ribonuclease A (RNase A) and stained with PI at room temperature for 30 min. Analysis was carried out using Flow cytometry. Further analysis and graphs were plotted using BD Flow Jo version10 software. (https://www.flowjo.com/flowjo/).

#### Western blotting

Protein isolation and western blotting were performed as described previously^69^. Briefly, IMR-32 cells treated with and without **9h** and **9m** for 24 h were washed with PBS and lysed in lysis buffer (Triton X (1%), NaCl (150 mM), Tris base (10 mM), EDTA (1 mM), EGTA (0.2 mM), IGEPAL (0.5%), and protease inhibitor (1 µl/ml)). The BCA method was used to determine the protein concentration in cell lysates and equal amounts of protein were loaded and separated by sodium dodecyl sulfate–polyacrylamide gel electrophoresis (SDS-PAGE) and transferred to the PVDF membrane. The membrane blocking was done using 5% skimmed milk for 2 h followed by overnight incubation at 4 °C using primary anti-acetyl SMC3 antibody (1:1000, Sigma, MABE1073). Then the membrane was incubated with secondary antibody-HRP conjugated anti-mouse IgG (1:20,000, Invitrogen, A16072) for 2 h at room temperature. Blots were stripped and re-probed with a β-actin antibody (1:100,000, Sigma, A1978) to check equal loading. The protein levels were quantified using ImageJ software (National Institutes of Health, Bethesda, Maryland) (https://imagej.net/ij/plugins/).

### Biochemcal enzyme activity assays

HDAC4 and 8 were produced in *E.coli* using previously published procedures^70,71^. HDAC10 and HDAC11 were produced similar to HDAC8 with the following changes: The genes were synthesized, codon-optimized, and subsequently cloned into a pET14b vector, in-frame with either a His₆-MBP tag or a His₆-SUMO tag. Protein expression was performed in LB medium and induced with 0.2 mM IPTG at an OD₆₀₀ of 0.6–0.8. Cultures were then incubated overnight at 16 °C. Both proteins were purified via immobilized metal affinity chromatography (IMAC), using a Ni-IDA column for HDAC11 and a cOmplete™ His-Tag Purification Column for hHDAC10. Following purification, the proteins were buffer-exchanged into SEC buffer, and the volume was concentrated by ultrafiltration. HDAC1 and 6 were purchased from BPS Bioscience (USA). For HDAC4 and HDAC8, a serial dilution of inhibitor in assay buffer (25 mM Tris–HCl, pH 8.0, 75 mM KCl, 0.00001% Pluronic F-127) was incubated with 10 nM HDAC8 or 1 nM in a black 96-well microplate (Greiner, Germany) for 60 min at 30 °C. The reaction was initiated by the addition of substrate (20 μM Boc-Lys(trifluoroacetyl)-7-amino-4-methylcoumarin) (Bachem, Switzerland). After incubation for 60 min at 30 °C, the reaction was stopped by the addition of 1.7 μM 9,9,9-trifluoro-8-oxo-N-phenylnonanamide and the deacetylated substrate was converted into a fluorescent product by the addition of 0.4 mg/ml trypsin (Roth, Germany). The release of 7-amino-4-methylcoumarin was monitored in a microplate reader at 450 nm (λEx = 350 nm) and correlated with enzyme activity. Dose–response curves were generated using GraphPad Prism and fitted to a four-parameter logistic function to obtain IC_50_ values:

$$EA = E_{0} + \frac{{\left( {E_{max} - E_{0} } \right)}}{{1 - 10^{{log\left( {IC50} \right) - x*h}} }}$$

where EA is the enzyme activity at a given inhibitor concentration x. E_max_ and E_0_ are the enzyme activities in the absence of ligand and under complete inhibition, respectively. IC_50_ is the ligand concentration at which half of the enzyme activity is inhibited. The parameter *h* is a measure of the steepness of the dose–response curve.

The assays for the other isoenzymes deviated slightly from this instruction: The assays for HDAC1 and HDAC6 reacted with 50 µM Boc-Lys(acetyl)-7-amino-4-methylcoumarin as substrate and were stopped with 4.3 µM SAHA. For HDAC10, 100 nM enzyme was used and the enzyme reaction was stopped with 3.8 µM quisinostat. For HDAC11, 100 nM enzyme and 80 µM Boc-Lys(trifluoroacetyl)-7-amino-4-methylcoumarin substrate were used and the reaction was stopped with 3.8 µM FT895. In addition, MAL buffer (137 mmol/L NaCl, 50 mmol/L Tris–HCl, 2.7 mmol/L KCl, 1 mmol/L MgCl2, 0.5 mg/mL bovine serum albumin (BSA), pH 8.0) was used instead of the assay buffer mentioned above.

#### Statistical analysis

The data were expressed as the mean ± SEM. Statistical tests were performed using GraphPad Prism Software version 8.0.2. (GraphPad Software, San Diego, California USA, www.graphpad.com). Comparisons of the observed data were analysed by one-way ANOVA. The statistical significance was set at *p < 0.05, ** *p* < 0.01, *** *p* < 0.001, **** *p* < 0.0001.

## Supplementary Information

Supplementary Information.

## Data Availability

Most of the data generated or analysed during this study are included in this published article and its supporting information file. Further information is available from the corresponding authors on reasonable request.

## Abbreviations

DMF: *N**,N*-Dimethylformamide
EtOAc: Ethyl acetate
DIPEA: *N**,N*-Diisopropylethylamine
TFA: Trifluoroacetic acid
HATU: 1-[Bis(dimethylamino)methylene]-1*H*-1,2,3-triazolo[4,5-*b*] pyridinium 3-oxid hexafluorophosphate
RT: Room temperature
TLC: Thin layer chromatography
Na_2_SO_4_: Sodium sulphate
