# Supplementary material for: Targeted HDAC8 inhibition with non-hydroxamate [1,2,4]triazolo[4,3-a] quinoline compounds
Source: Sci Rep. 2026 Feb 20;16:7472. doi: 10.1038/s41598-026-38490-y (PMC12929686; doi:10.1038/s41598-026-38490-y)
Supplement: Supplementary file 1 — Supplementary Information. [file 41598_2026_38490_MOESM1_ESM.docx]

**Supporting Information**

**Targeted HDAC8 Inhibition with Non-Hydroxamate s85
[1,2,4]Triazolo[4,3-a] Quinoline Compounds**

N.V.M. Rao Bandaru^a,c^, Ashna Fathima^b^, Suryansh Sengar^b^, Markus Schweipert^d,e^,
Kosana Sai Chaitanya^a^, Muzaffar-Ur-Rehman Mohammed,^f^ Suraj T. Gore^c^, Trinath Jamma^b^, Vivek Sharma^b^, Chandrasekhar Abbineni^c^, Franz-Josef Meyer-Almes*^d,e^, Kondapalli Venkata Gowri Chandra Sekhar*^a^

*^a^Department of Chemistry, Birla Institute of Technology and Science, Pilani, Hyderabad Campus, Jawahar Nagar, Hyderabad 500 078, Telangana, India*

*^b^Department of Biological Sciences, Birla Institute of Technology and Science, Pilani, Hyderabad Campus, Jawahar Nagar, Hyderabad 500 078, Telangana, India*

*^c^Aurigene Oncology Limited, 39-40 KIADB Industrial Area Electronic City Phase II, Hosur Road, Bangalore 560 100, India*

*^d^Department of Chemical Engineering and Biotechnology, University of Applied Sciences Darmstadt, Haardtring 100, 64295 Darmstadt, Germany.*

*^e^European University of Technology, European Union*

*^f^Department of Pharmacy, Birla Institute of Technology and Science, Pilani Campus,*

*Pilani 333031, Rajasthan, India*

*Corresponding authors

E-mail: [kvgc@hyderabad.bits-pilani.ac.in](mailto:kvgc@hyderabad.bits-pilani.ac.in) (KVGCS); [franz-josef.meyer-almes@h-da.de](mailto:franz-josef.meyer-almes@h-da.de) (Franz)

**List of contents:**

| 1. | Analytical data of final compounds **9a** to **9u** |  |
| --- | --- | --- |
| 2. | IC50 graphs |  |
| 3. | Original blots of **9h** and **9m** |  |

1. **Analytical data of final compounds 9a to** **9u**


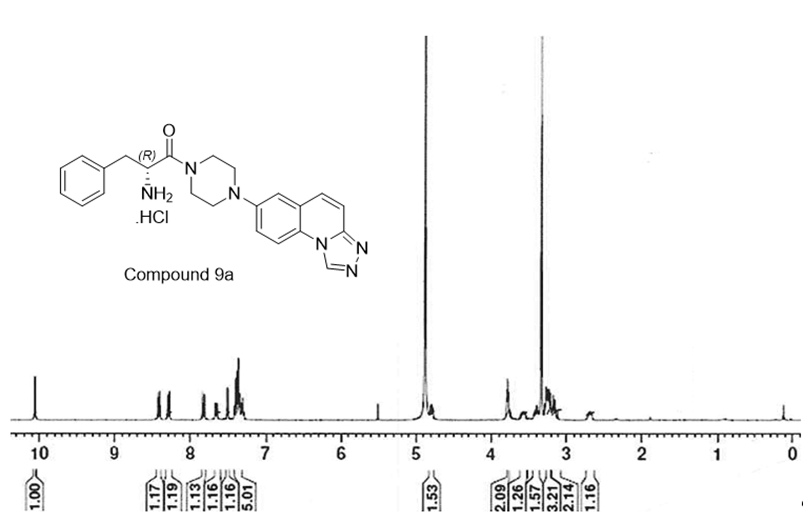


**Figure S1.** ^1^H NMR spectrum of compound **9a** in **CD3OD**


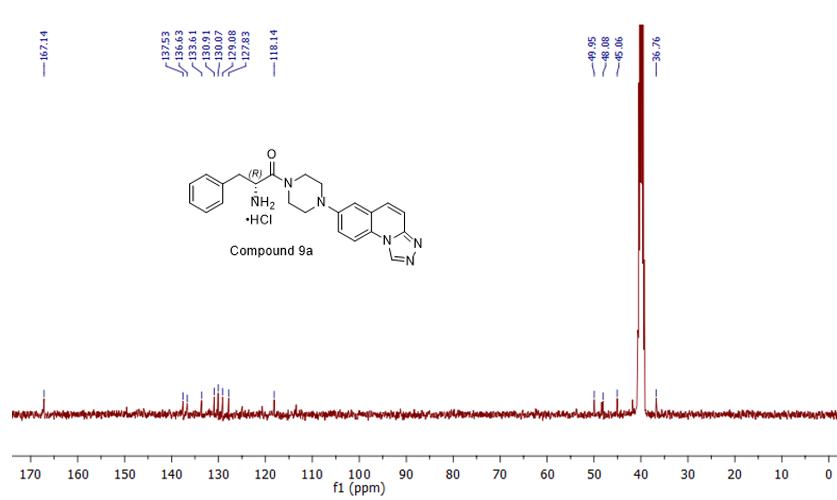


**Figure S2.** ^13^C NMR spectrum of compound **9a** in **DMSO-d_6_**


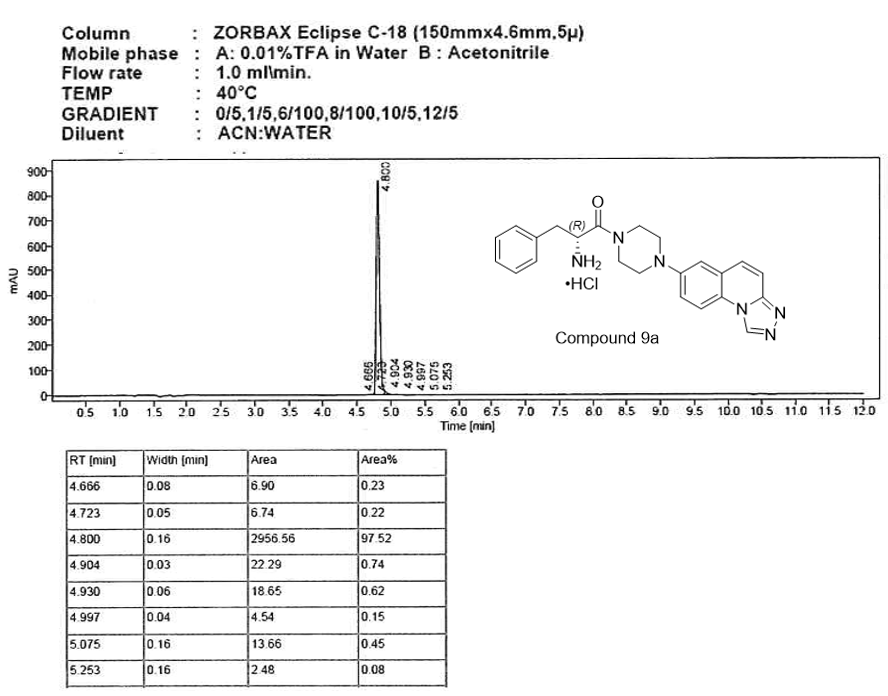


**Figure S3.** The HPLC chromatogram of compound **9a**


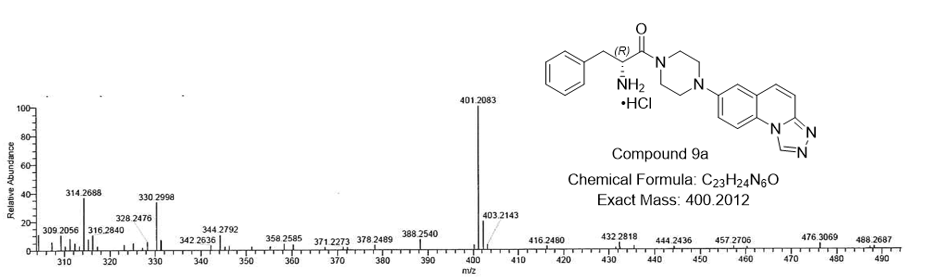


**Figure S4.** The HRMS chromatogram of compound **9a**


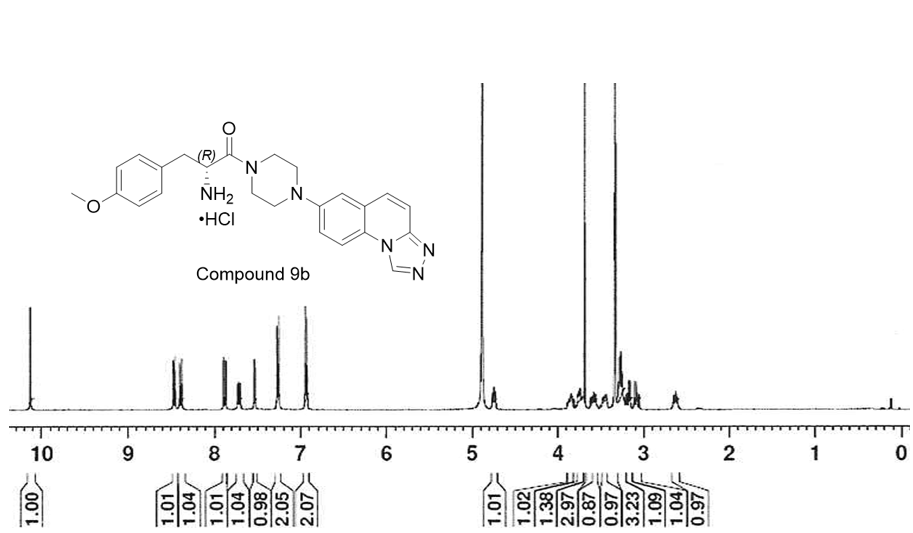


**Figure S5.** The ^1^H NMR spectrum of compound **9b** in **CD3OD**


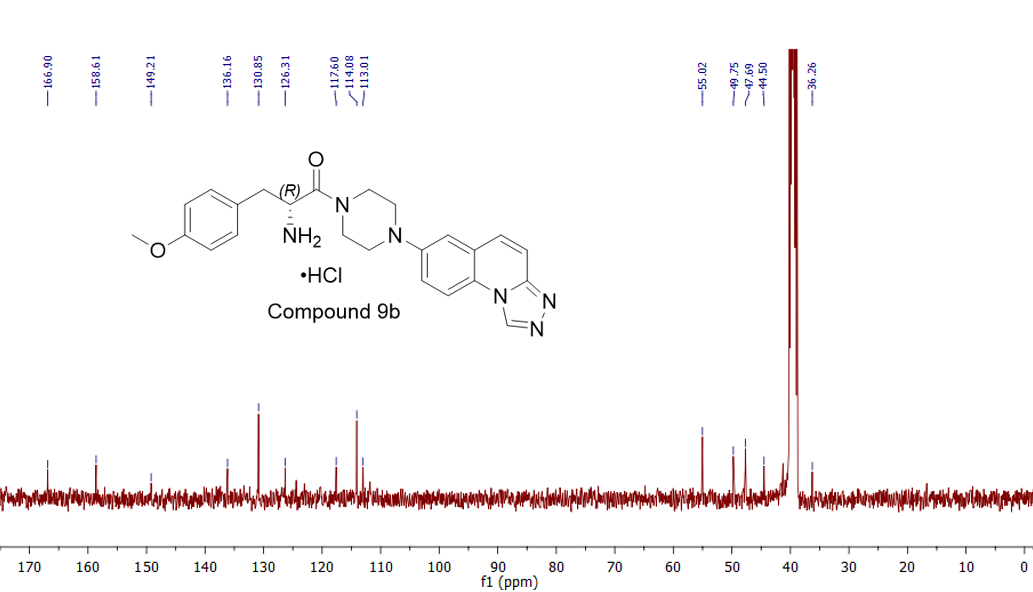


**Figure S6.** The ^13^C NMR spectrum of compound **9b** in **DMSO-d_6_**


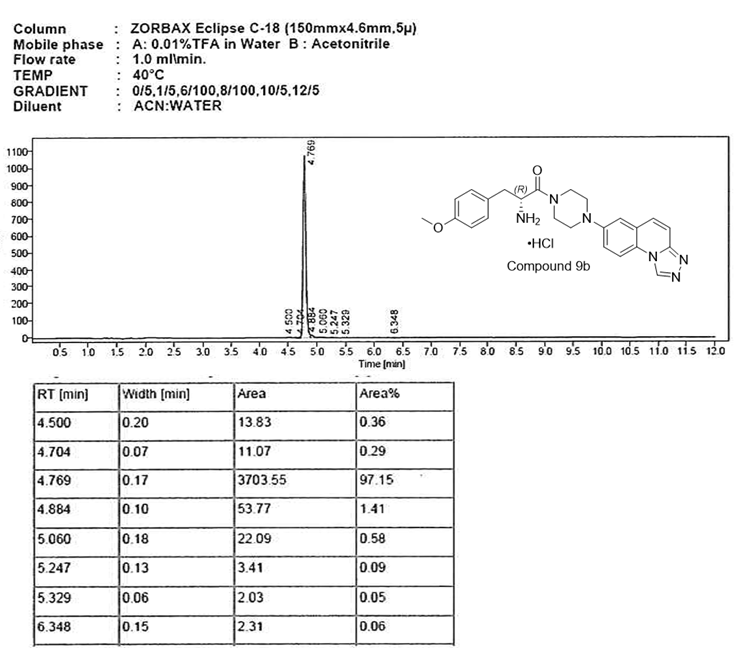


**Figure S7.** The HPLC chromatogram of compound **9b**


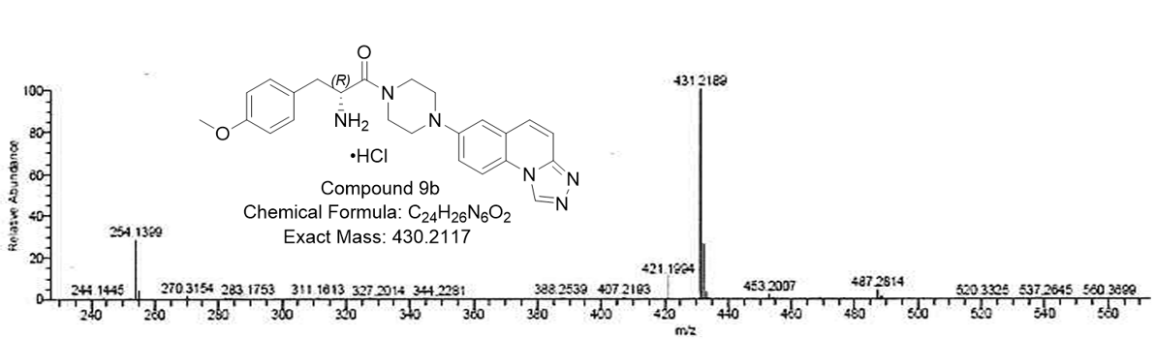


**Figure S8.** The HRMS chromatogram of compound **9b**


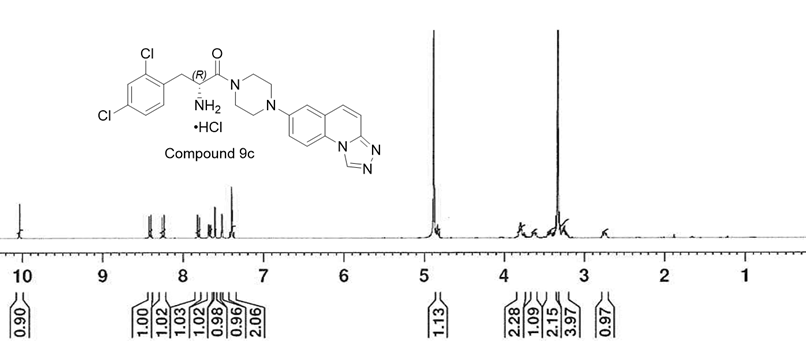


**Figure S9.** The ^1^H NMR spectrum of compound **9c** in **CD3OD**


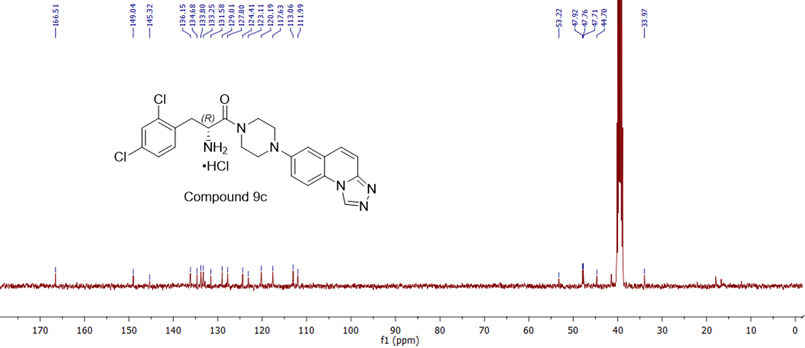


**Figure S10.** The ^13^C NMR spectrum of compound **9c** in **DMSO-d_6_**


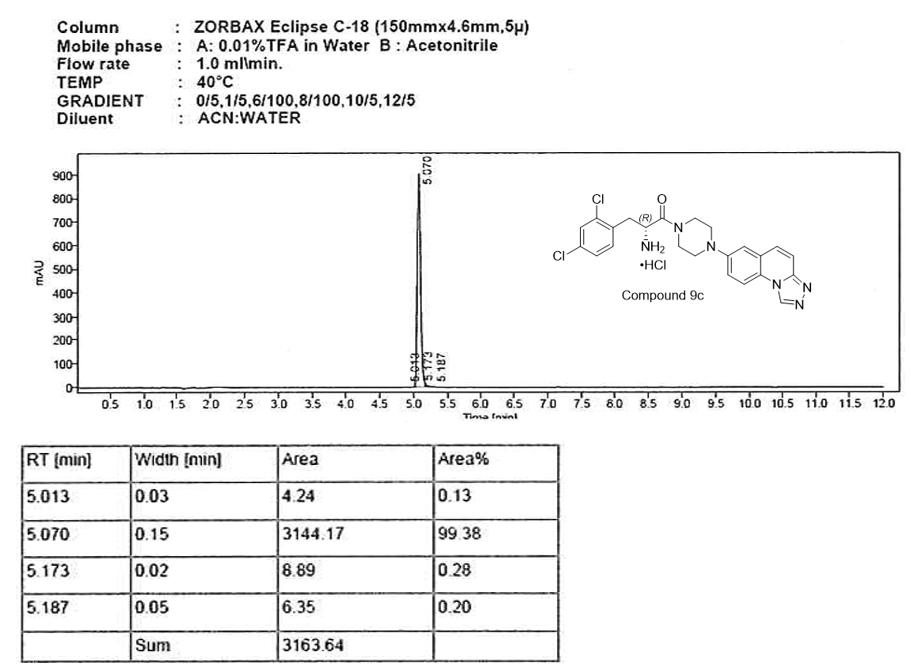


**Figure S11.** The HPLC chromatogram of compound **9c**


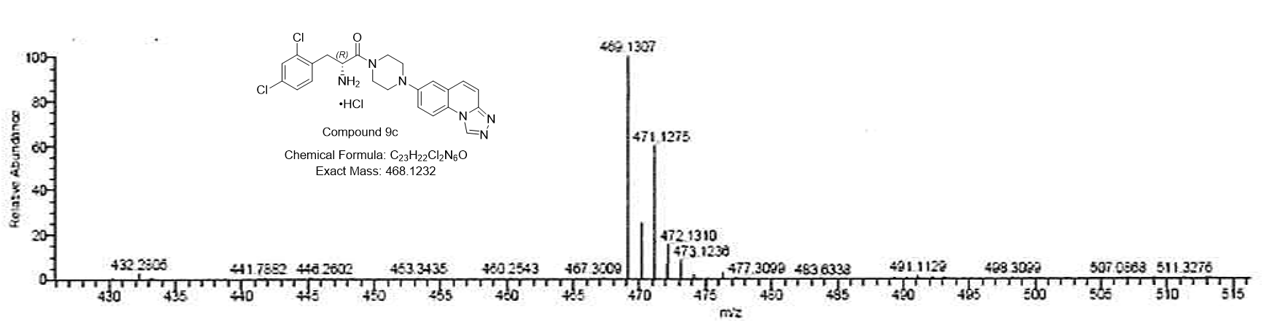


**Figure S12.** The HRMS chromatogram of compound **9c**


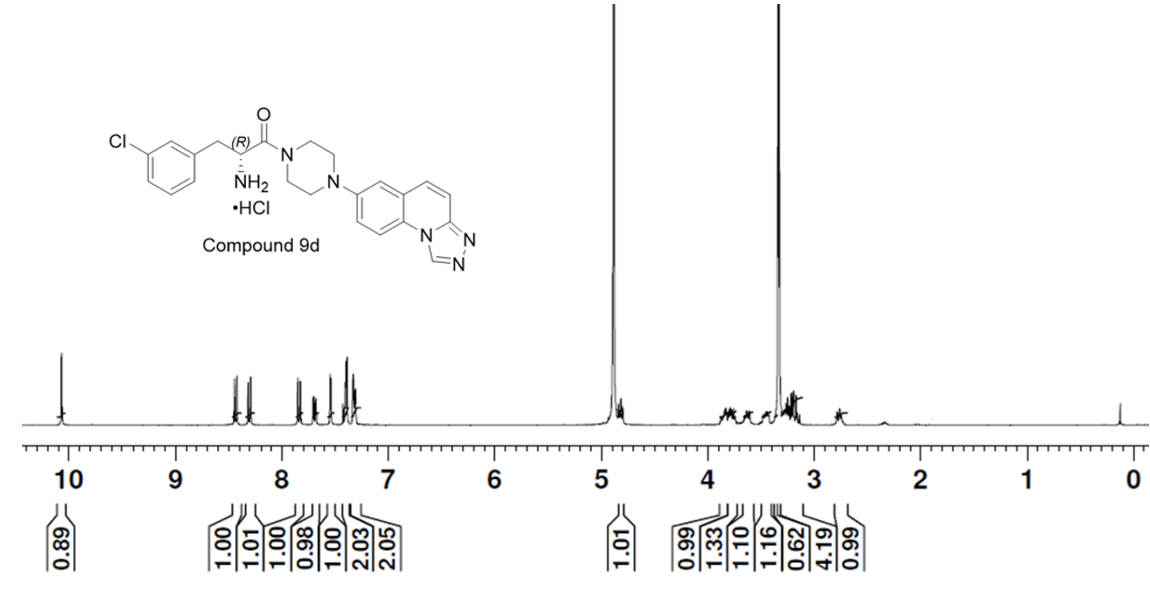


**Figure S13.** The ^1^H NMR spectrum of compound **9d** in **CD3OD**


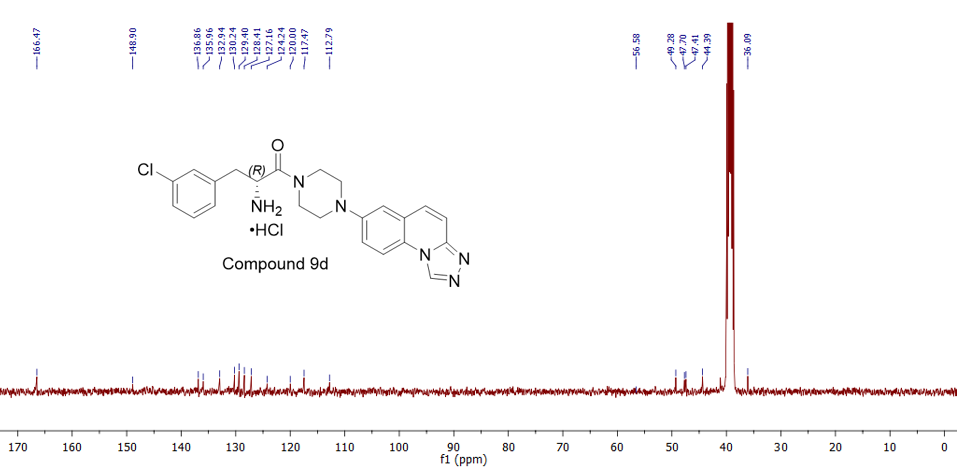


**Figure S14.** The ^13^C NMR spectrum of compound **9d** in **DMSO-d_6_**


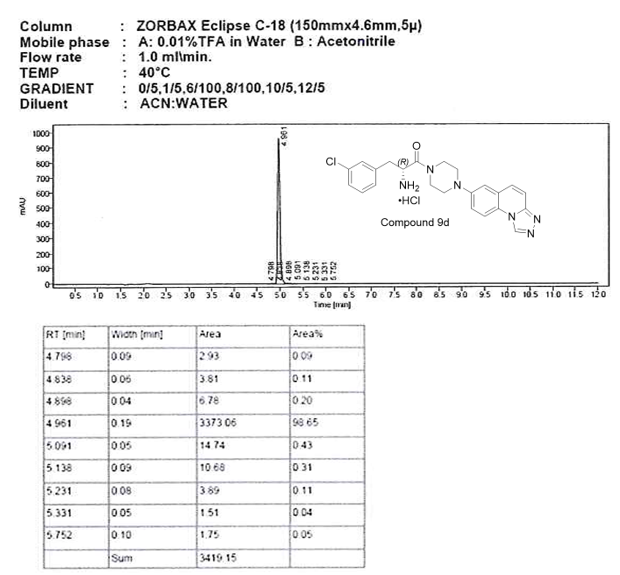


**Figure S15.** The HPLC chromatogram of compound **9d**


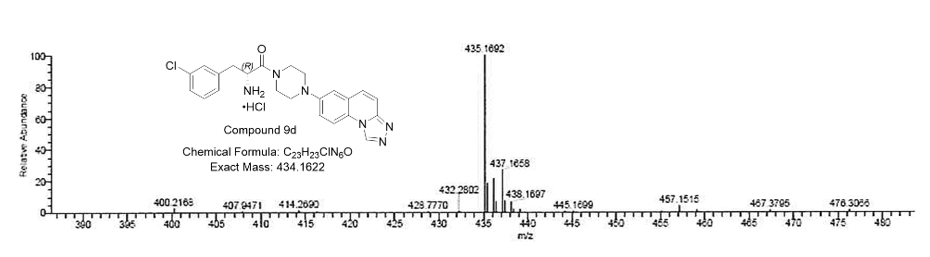


**Figure S16.** The HRMS chromatogram of compound **9d**


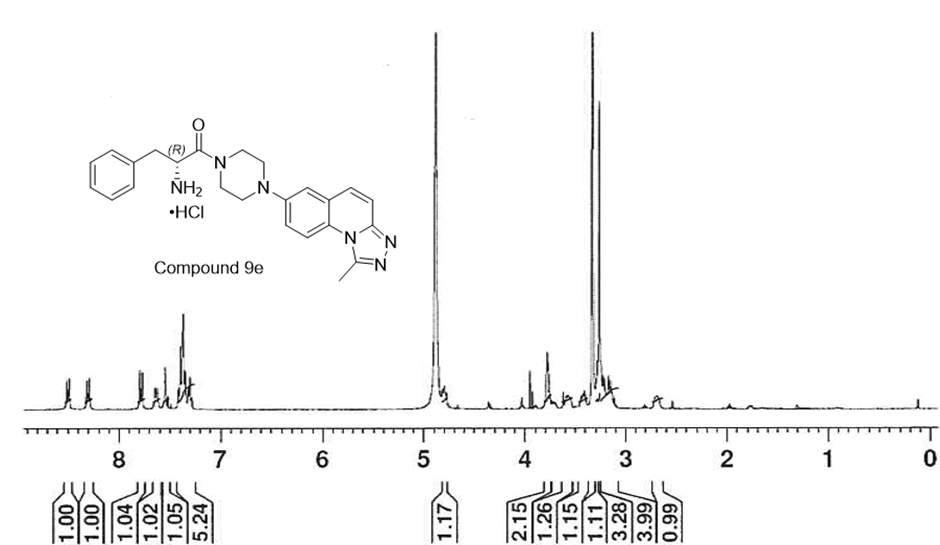


**Figure S17.** The ^1^H NMR spectrum of compound **9e** in **CD3OD**


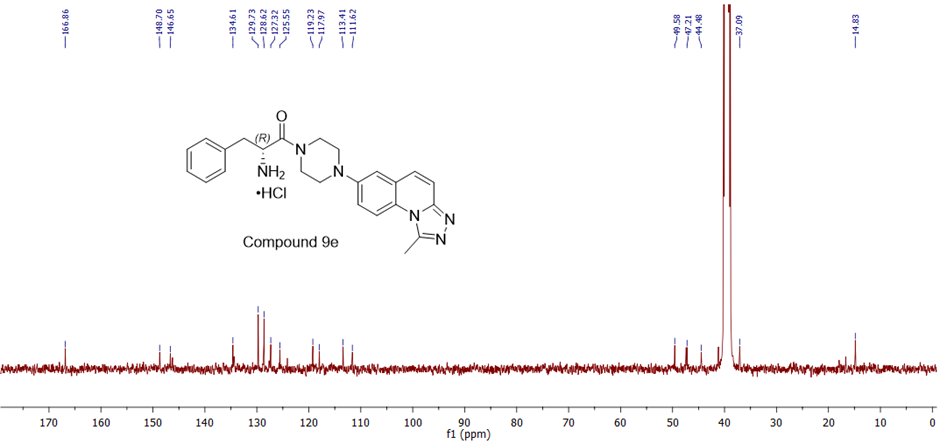


**Figure S18.** The ^13^C NMR spectrum of compound **9e** in **DMSO-d_6_**


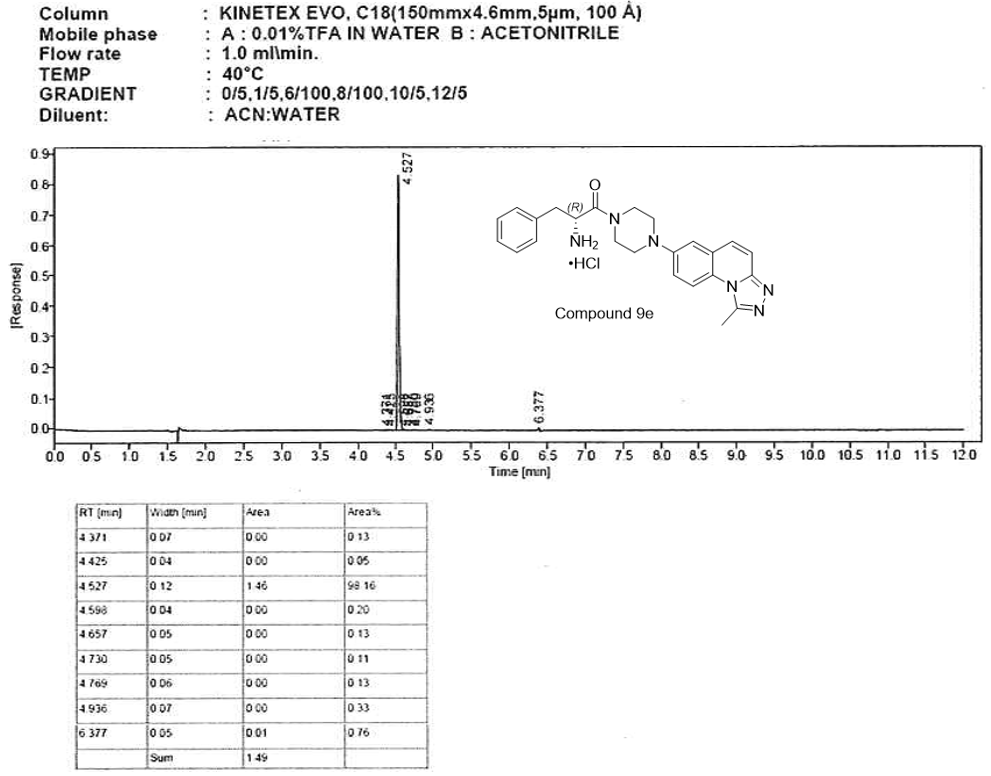


**Figure S19.** The HPLC chromatogram of compound **9e**


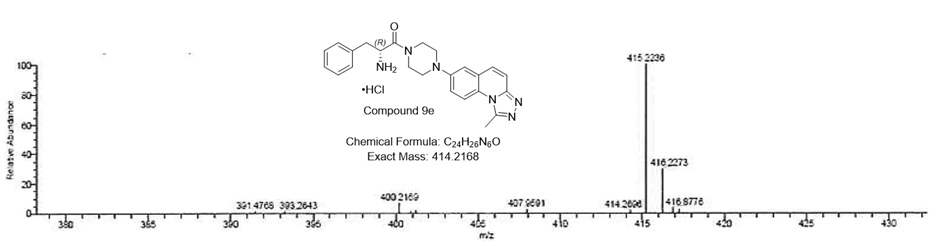


**Figure S20.** The HRMS chromatogram of compound **9e**


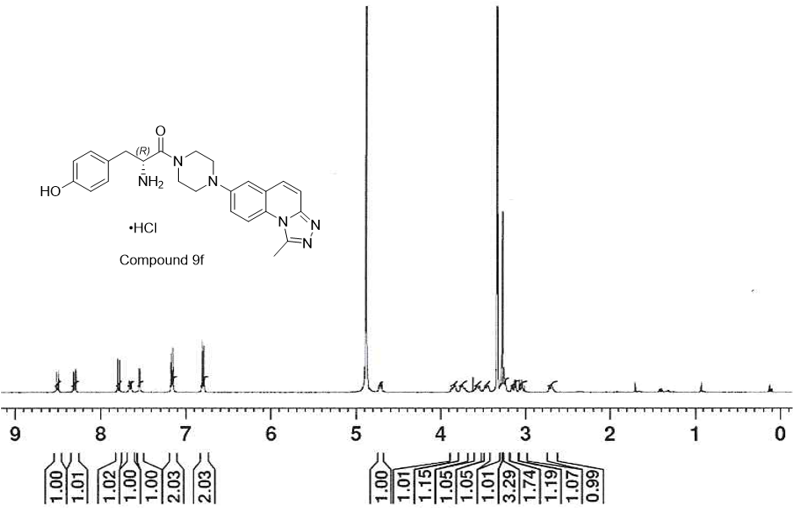


**Figure S21.** The ^1^H NMR spectrum of compound **9f** in **CD3OD**


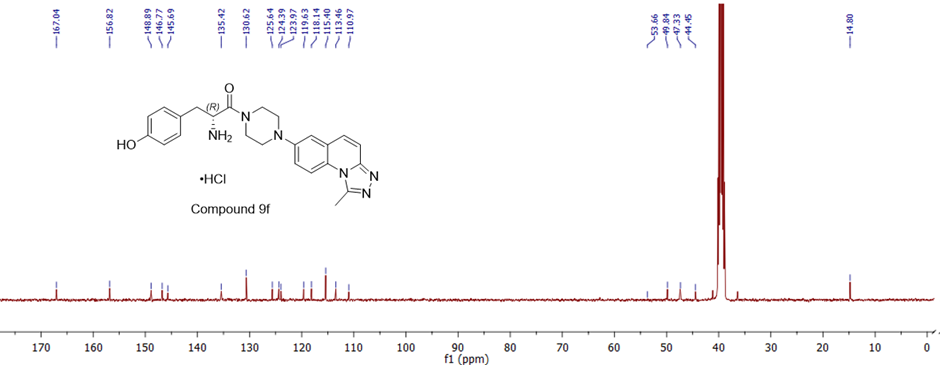


**Figure S22.** The ^13^C NMR spectrum of compound **9f** in **DMSO-d_6_**


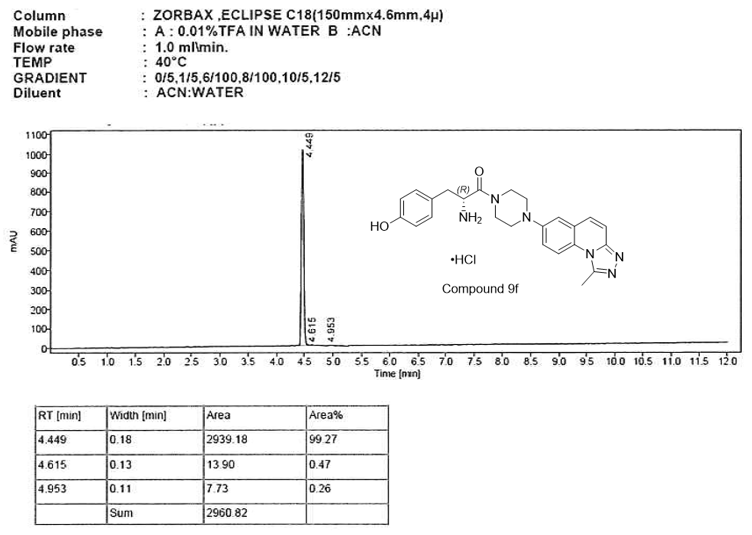


**Figure S23.** The HPLC chromatogram of compound **9f**


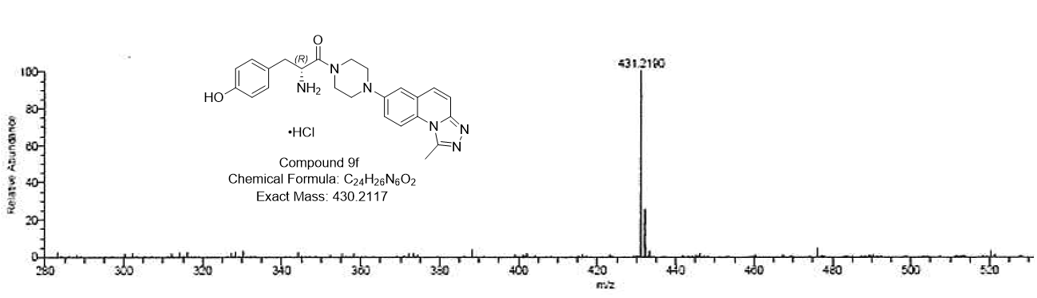


**Figure S24.** The HRMS chromatogram of compound **9f**


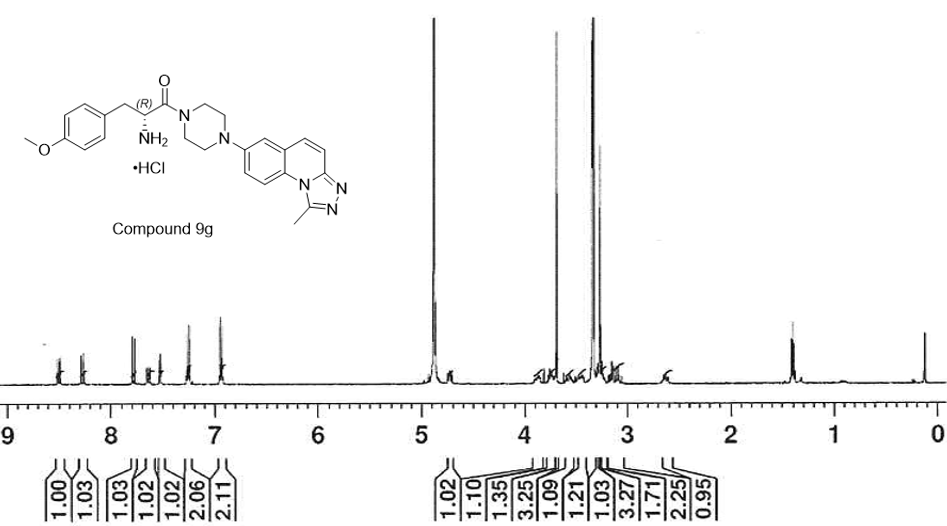


**Figure S25.** The ^1^H NMR spectrum of compound **9g** in **CD3OD**


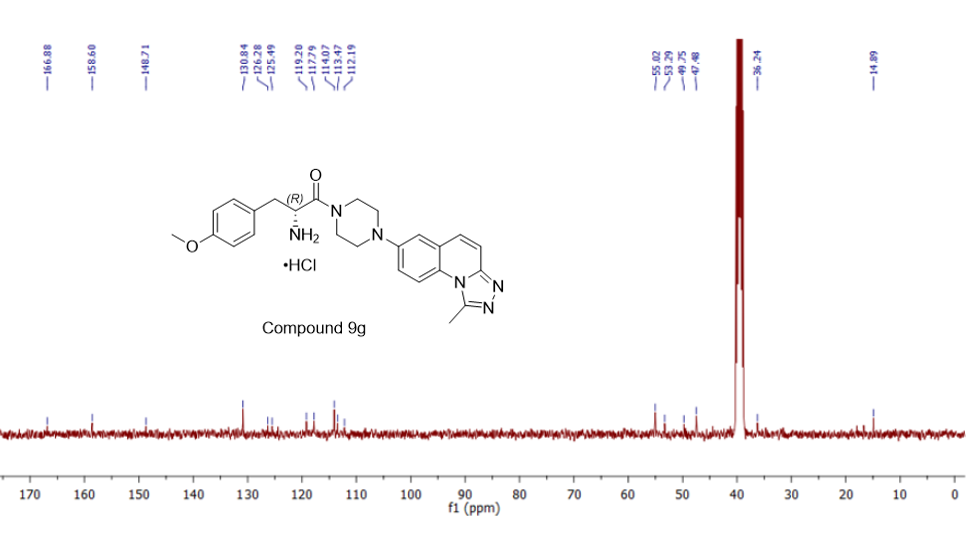


**Figure S26.** The ^13^C NMR spectrum of compound **9g** in **DMSO-d_6_**


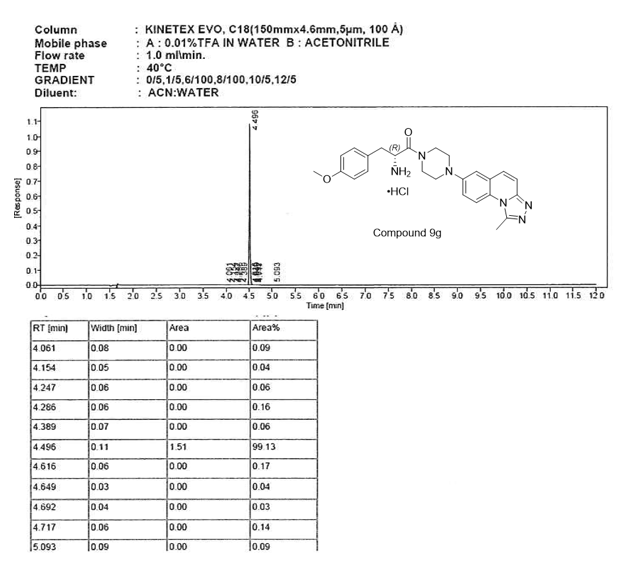


**Figure S27.** The HPLC chromatogram of compound **9g**


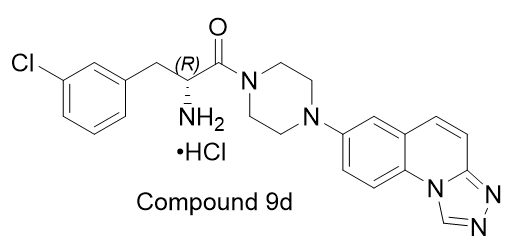

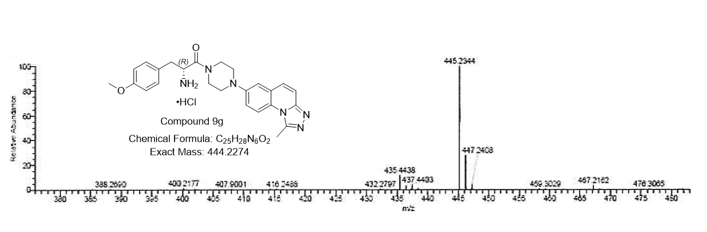


**Figure S28.** The HRMS chromatogram of compound **9g**


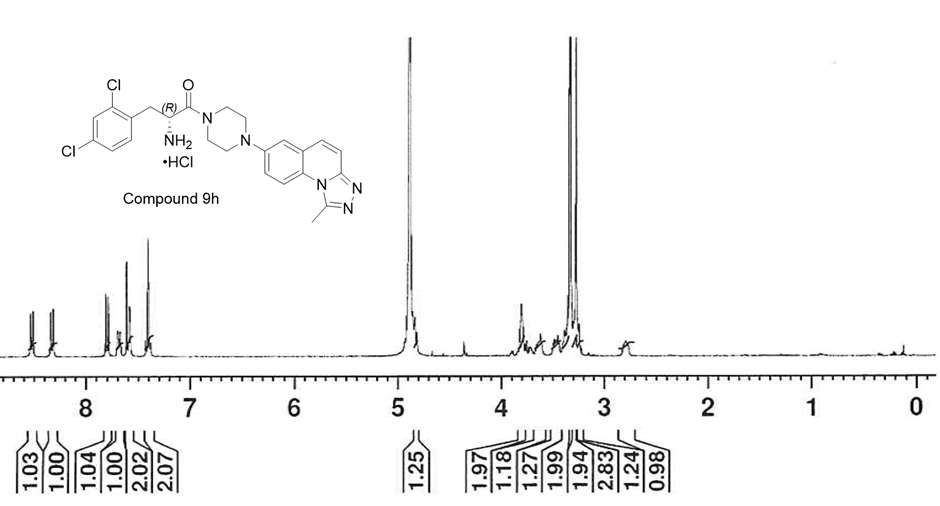


**Figure S29.** The ^1^H NMR spectrum of compound **9h** in **CD3OD**


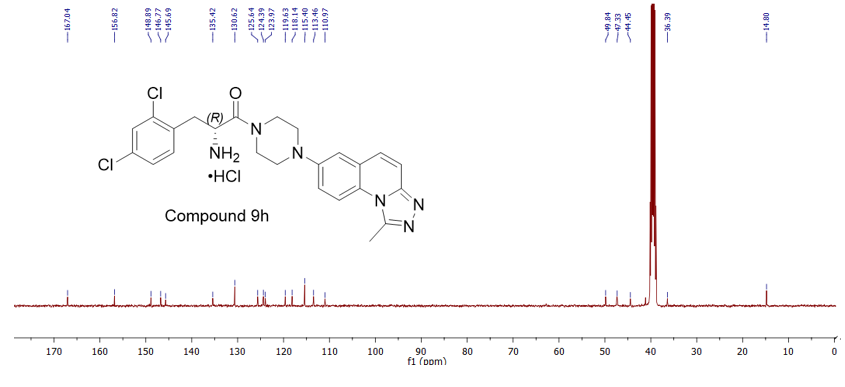


**Figure S30.** The ^13^C NMR spectrum of compound **9h** in **DMSO-d_6_**


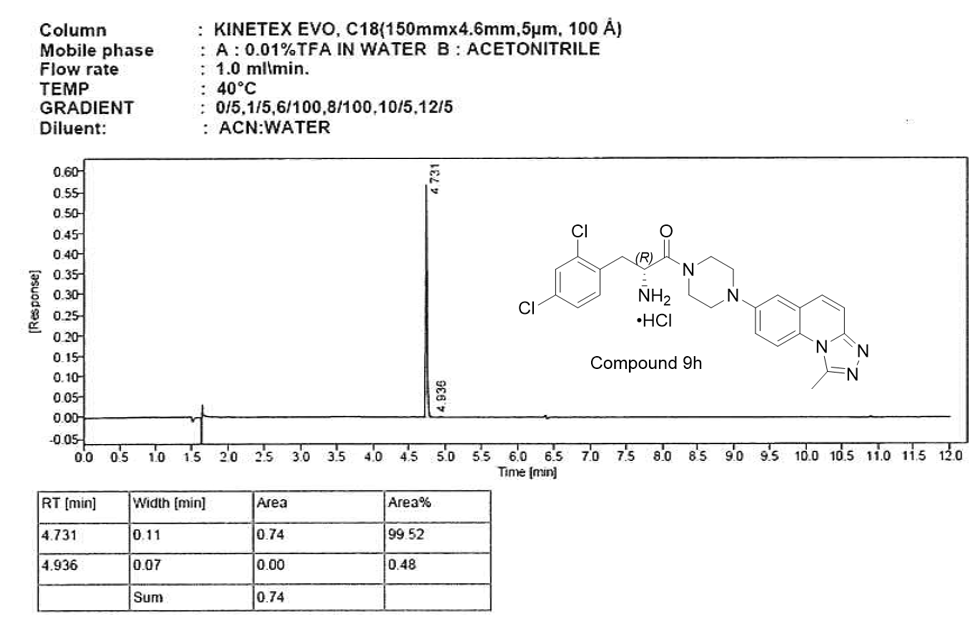


**Figure S31.** The HPLC chromatogram of compound **9**
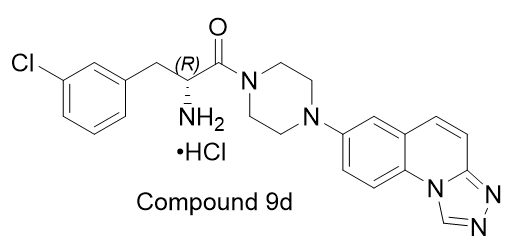
**h**


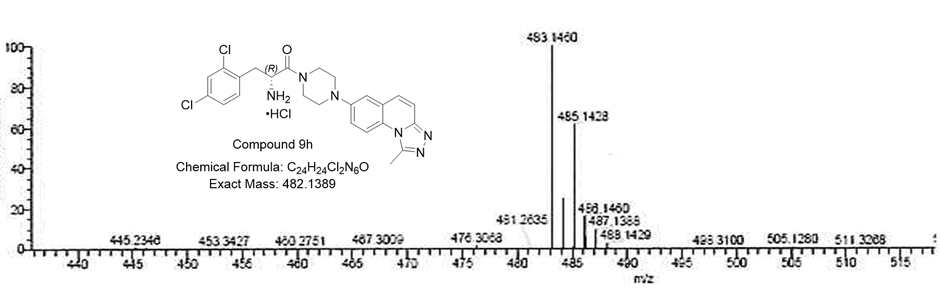


**Figure S32.** The HRMS chromatogram of compound **9h**


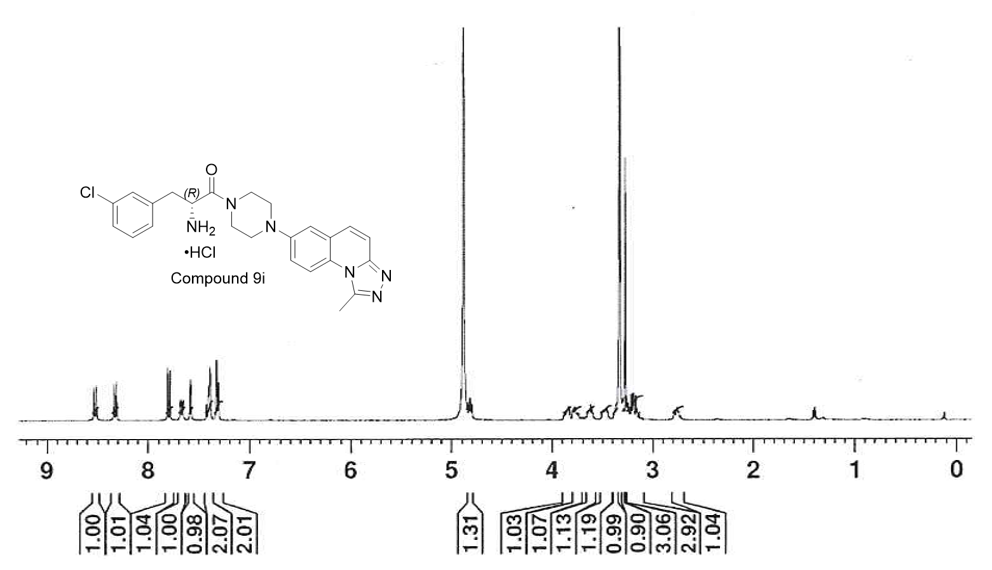


**Figure S33.** The ^1^H NMR spectrum of compound **9i** in **CD3OD**


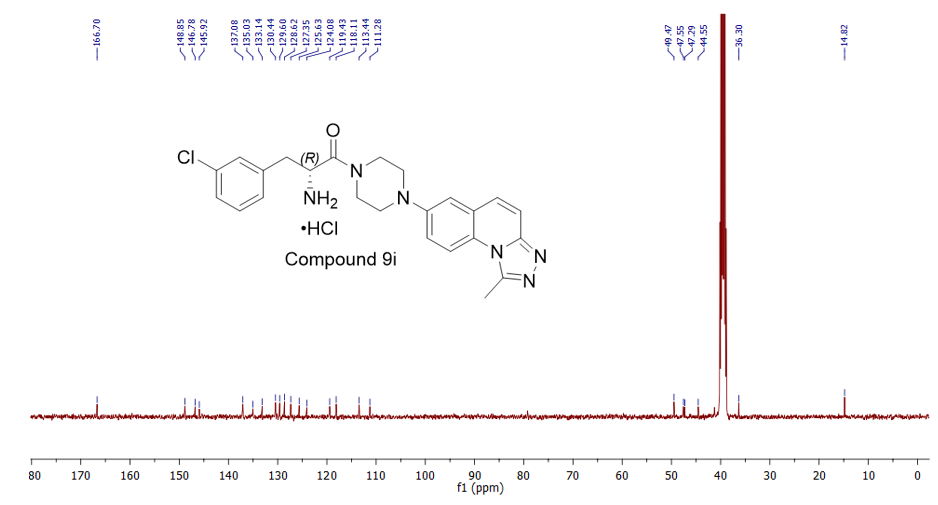


**Figure S34.** The ^13^C NMR spectrum of compound **9i** in **DMSO-d_6_**


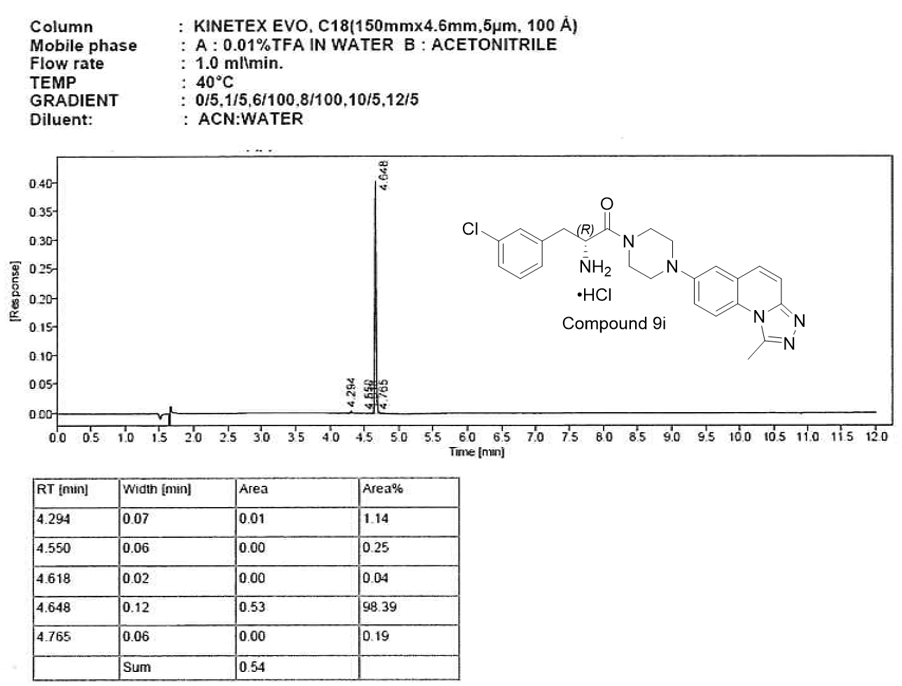


**Figure S35.** The HPLC chromatogram of compound **9**
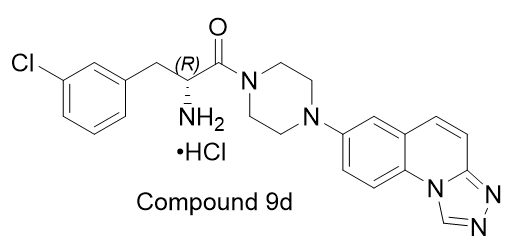
**i**


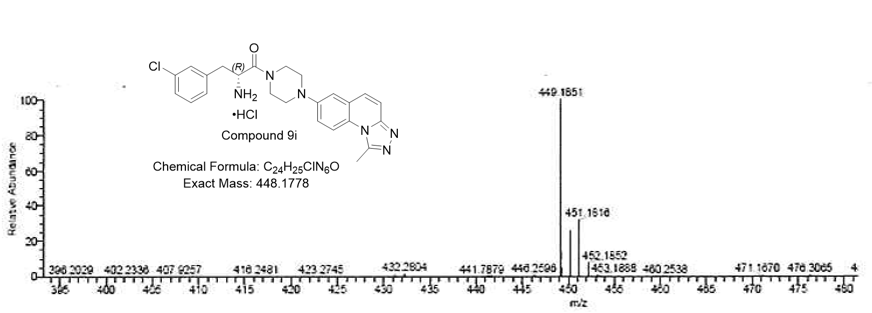


**Figure S36.** The HRMS chromatogram of compound **9i**


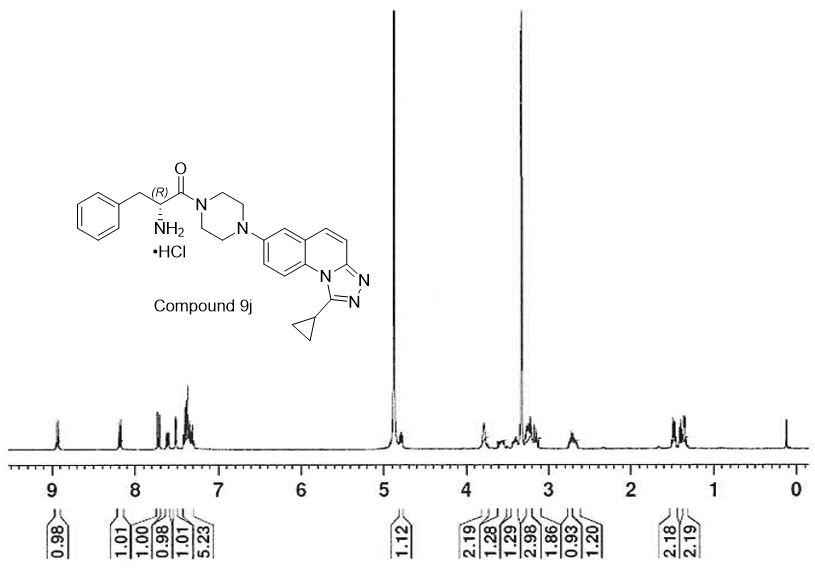


**Figure S37.** The ^1^H NMR spectrum of compound **9j** in **CD3OD**


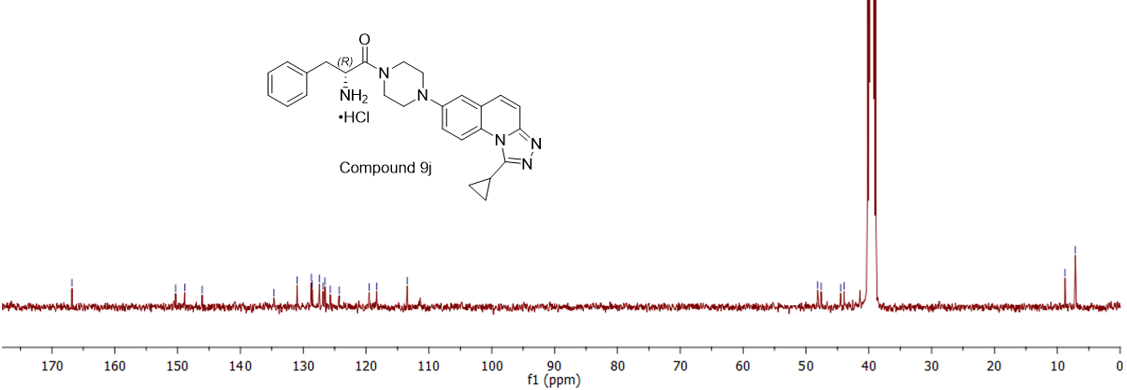


**Figure S38.** The ^13^C NMR spectrum of compound **9j** in **DMSO-d_6_**


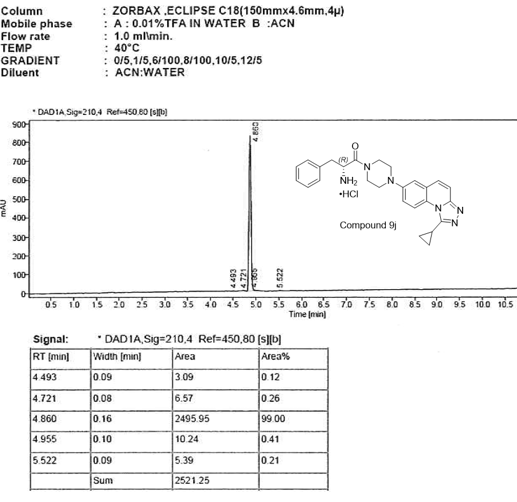


**Figure S39.** The HPLC chromatogram of compound **9j**


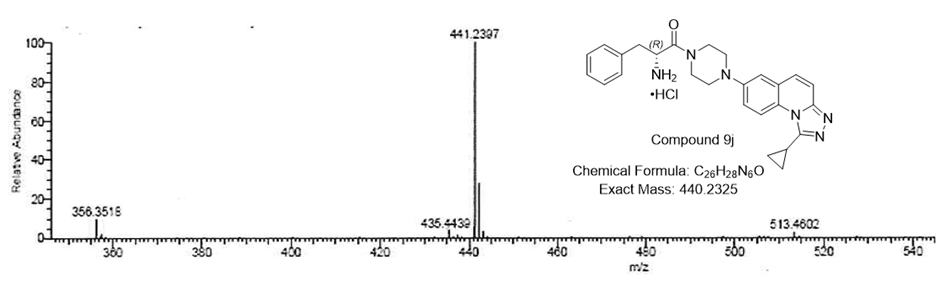


**Figure S40.** The HRMS chromatogram of compound **9j**


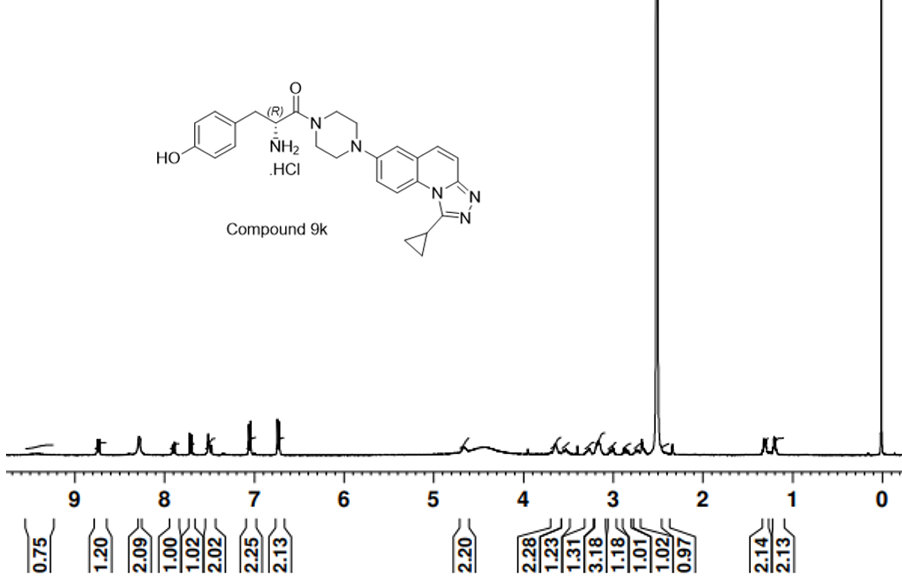


**Figure S41.** The ^1^H NMR spectrum of compound **9k** in **DMSO-d**_6_


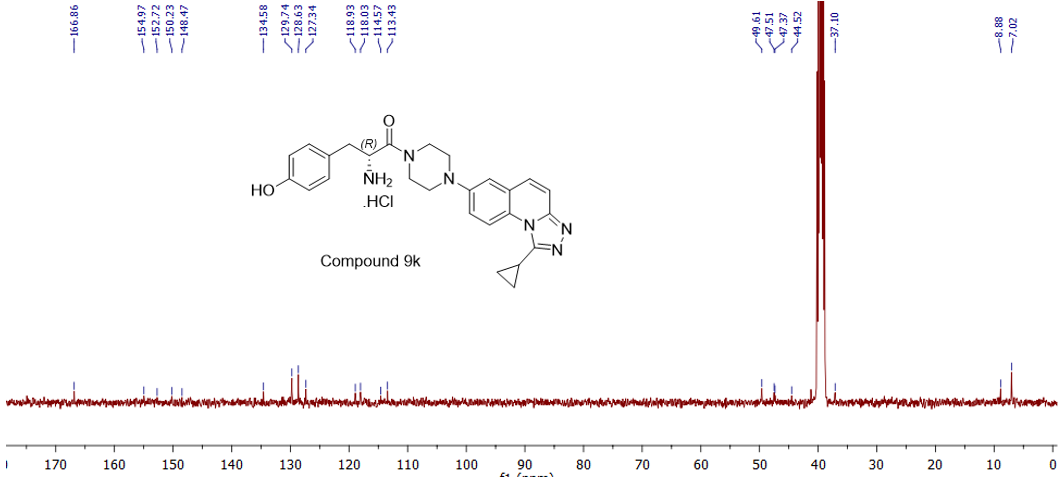


**Figure S42.** The ^13^C NMR spectrum of compound **9k** in **DMSO-d**_6_


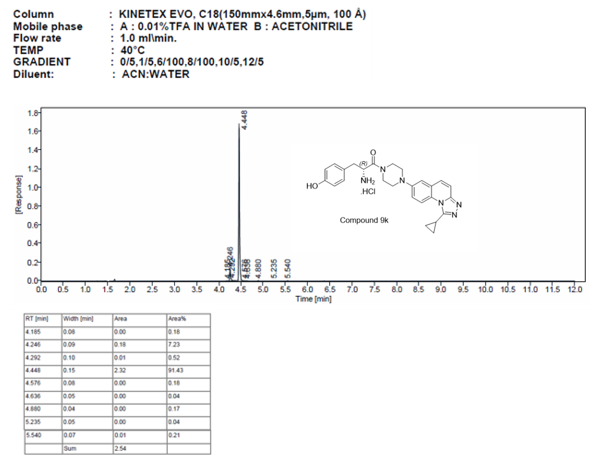


**Figure S43.** The HPLC chromatogram of compound **9**
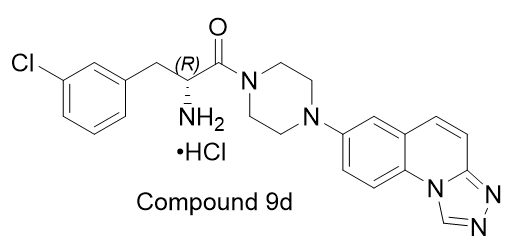
**k**


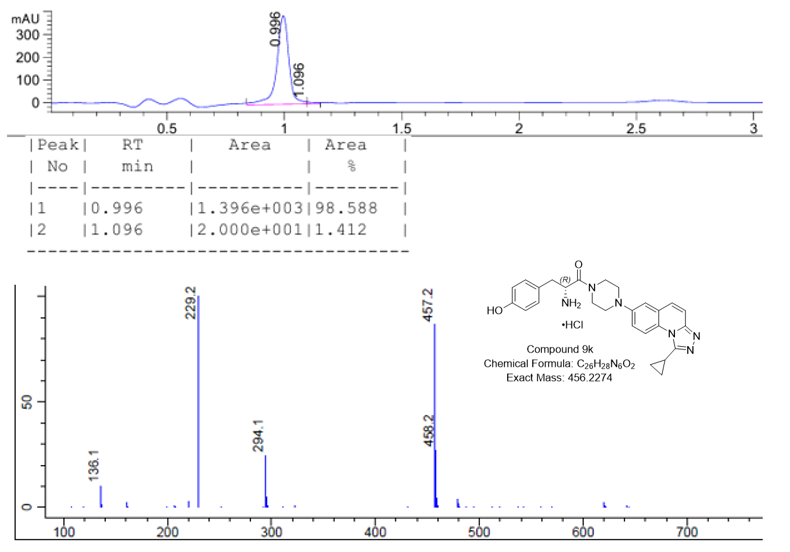


**Figure S44.** The LC-MS chromatogram of compound **9**
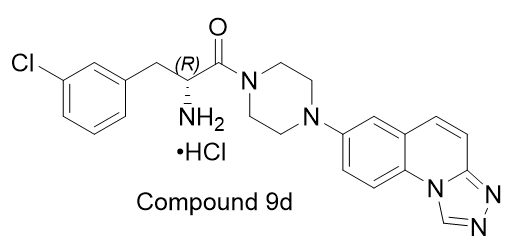
**k**


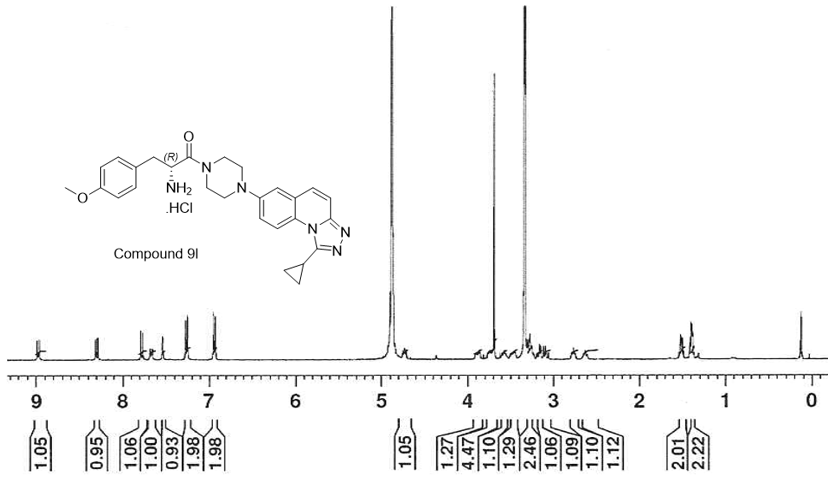


**Figure S45.** The ^1^H NMR spectrum of compound **9l** in **CD3OD**


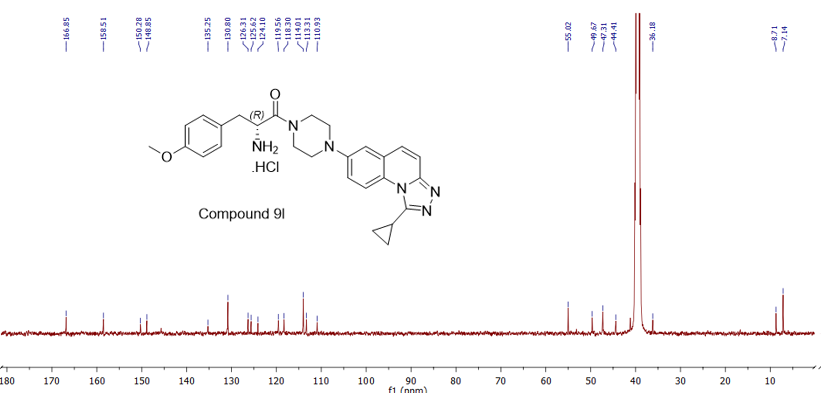


**Figure S46.** The ^13^C NMR spectrum of compound **9l** in **DMSO-d**_6_


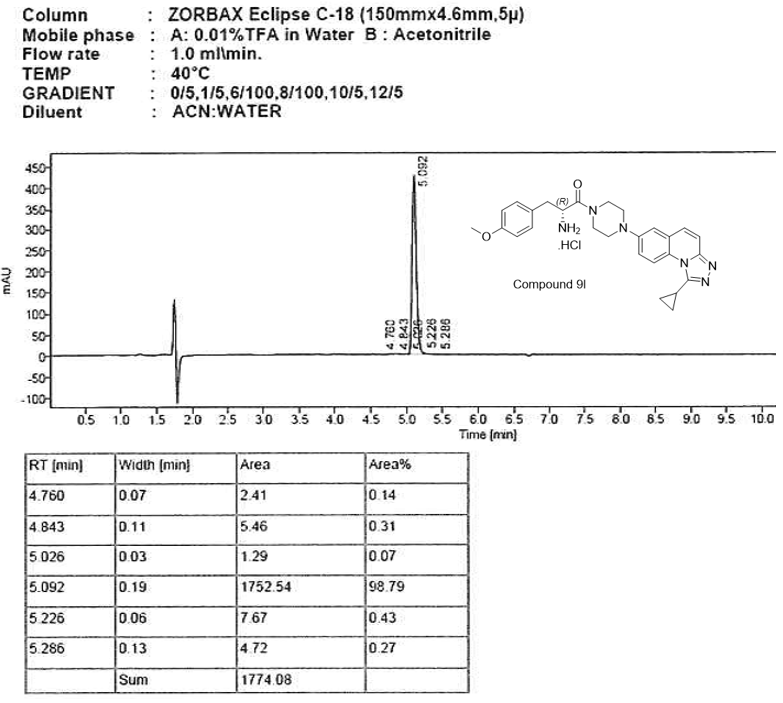


**Figure S47.** The HPLC chromatogram of compound **9**
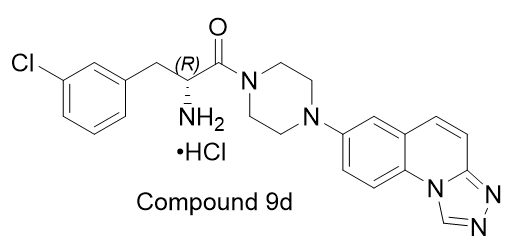
**l**


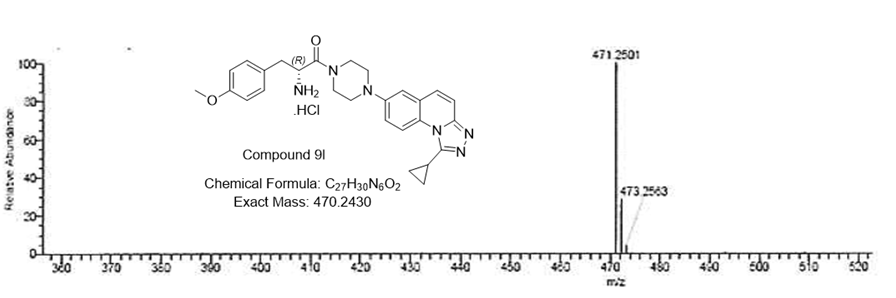


**Figure S48.** The HRMS chromatogram of compound **9l**


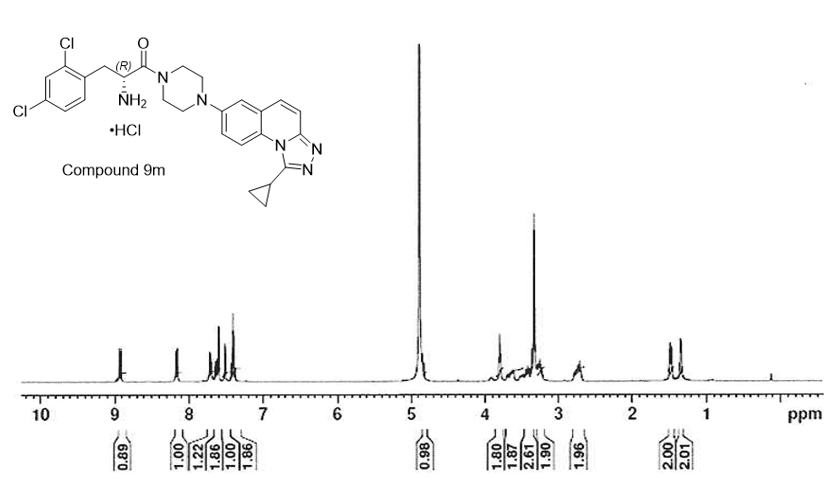


**Figure S49.** The ^1^H NMR spectrum of compound **9m** in **CD3OD**


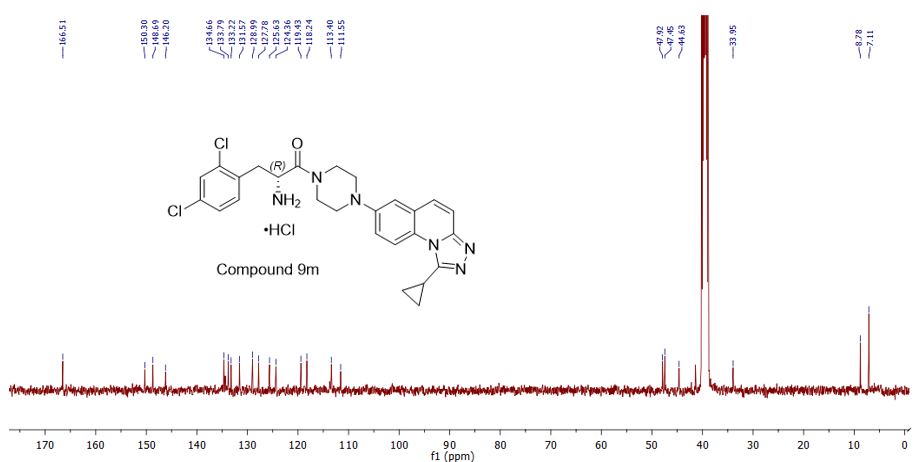


**Figure S50.** The ^13^C NMR spectrum of compound **9m** in **DMSO-d**_6_


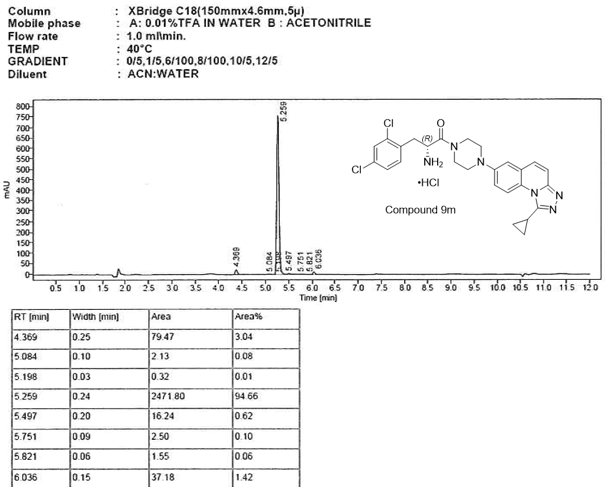


**Figure S51.** The HPLC chromatogram of compound **9**
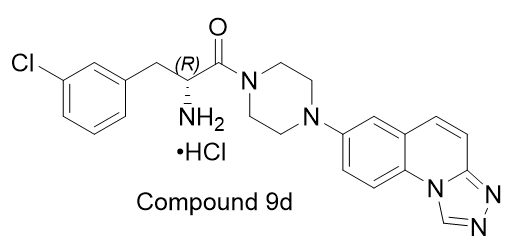
**m**


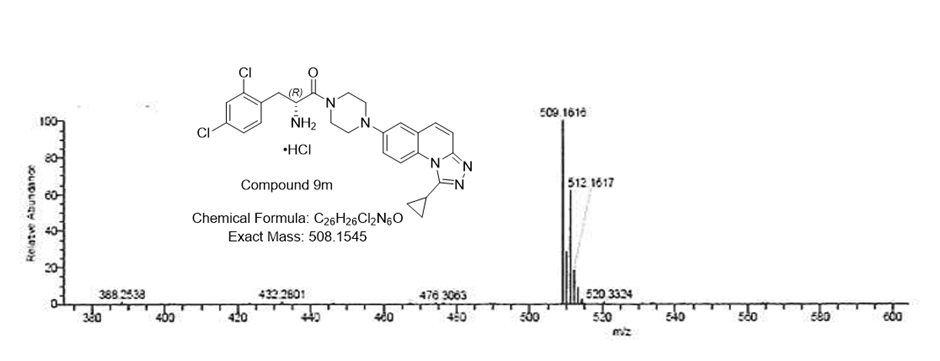


**Figure S52.** The HRMS chromatogram of compound **9m**


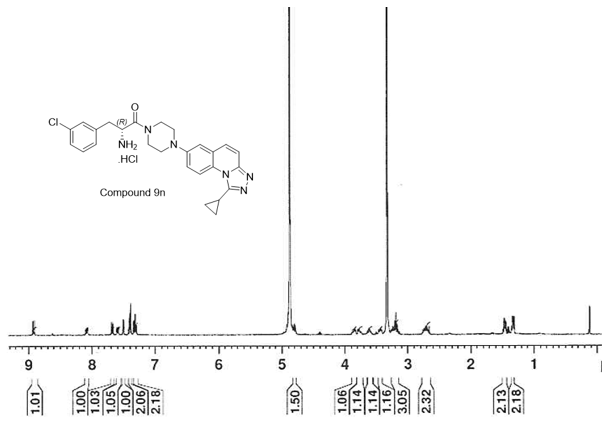


**Figure S53.** The ^1^H NMR spectrum of compound **9n** in **CD3OD**


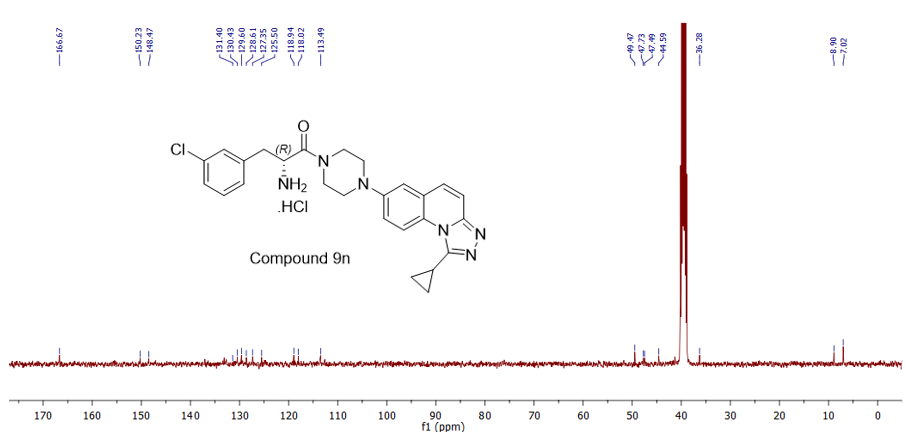


**Figure S54.** The ^13^C NMR spectrum of compound **9n** in **DMSO-d**_6_


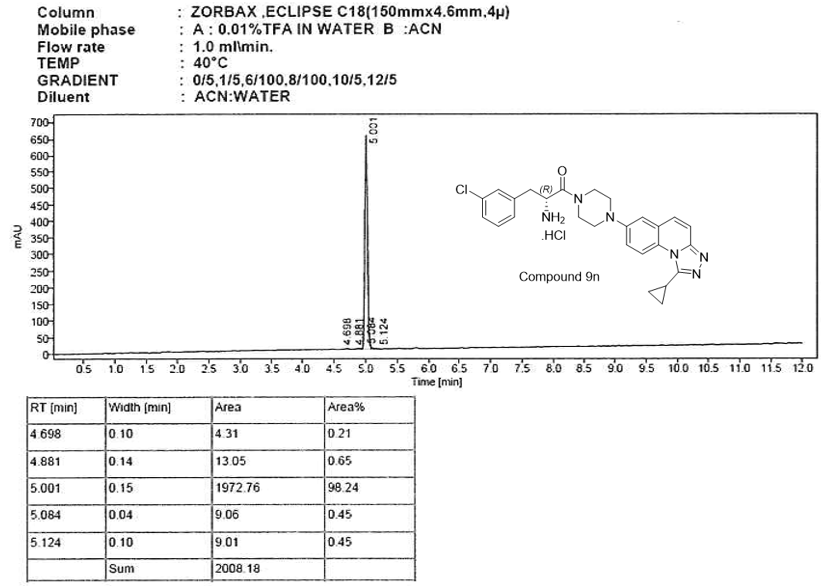


**Figure S55.** The HPLC chromatogram of compound **9**
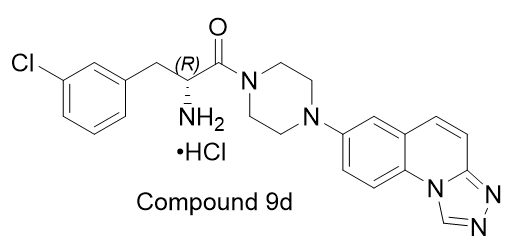
**n**


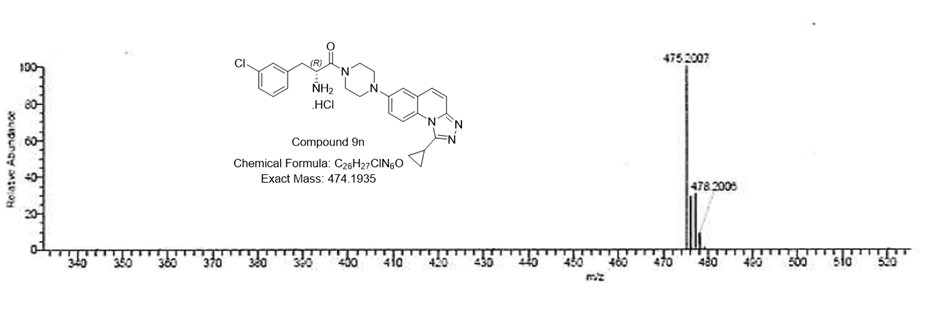


**Figure S56.** The HRMS chromatogram of compound **9n**


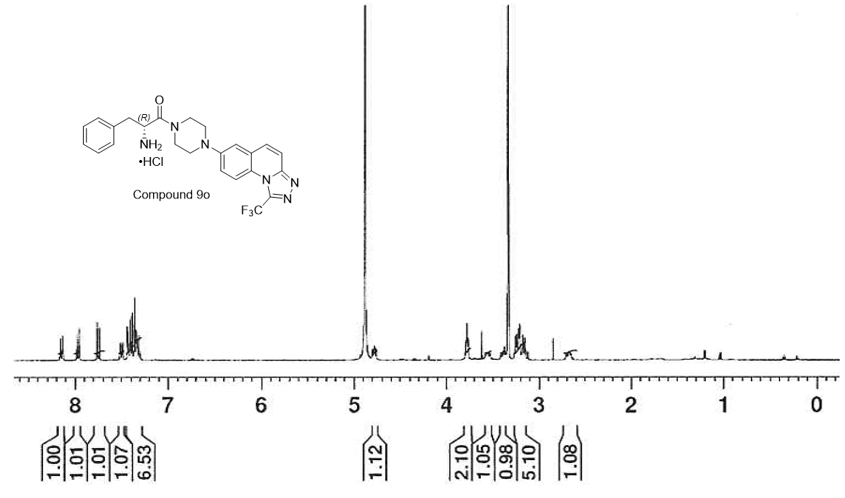


**Figure S57.** The ^1^H NMR spectrum of compound **9o** in **CD3OD**


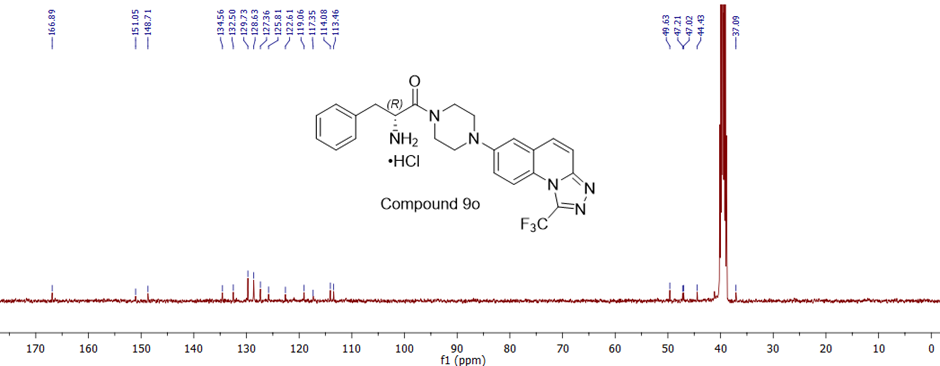


**Figure S58.** The ^13^C NMR spectrum of compound **9o** in **DMSO-d**_6_


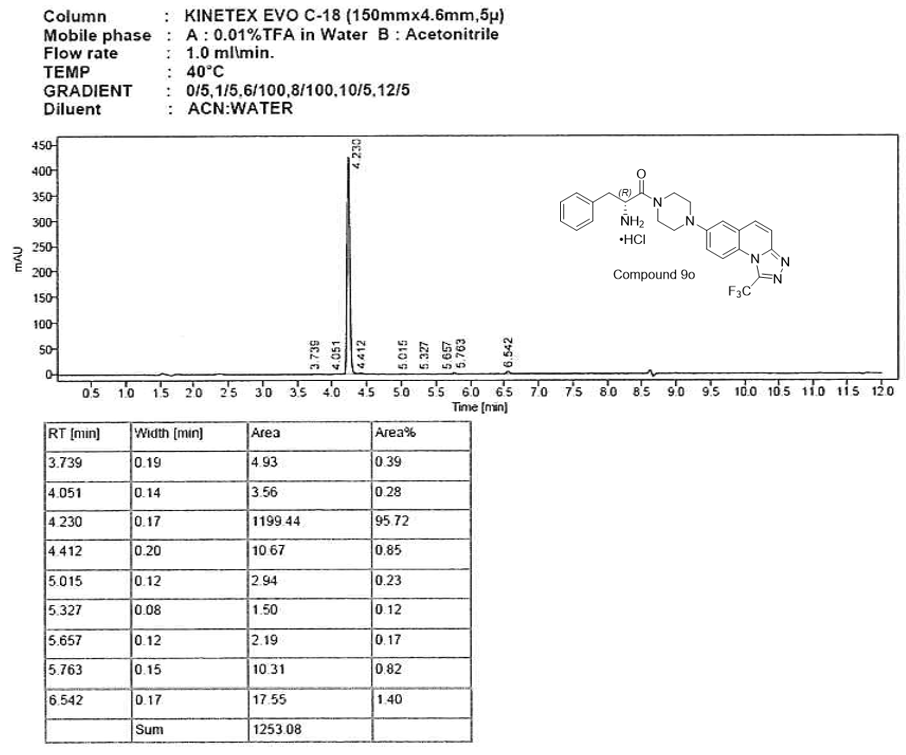


**Figure S59.** The HPLC chromatogram of compound **9**
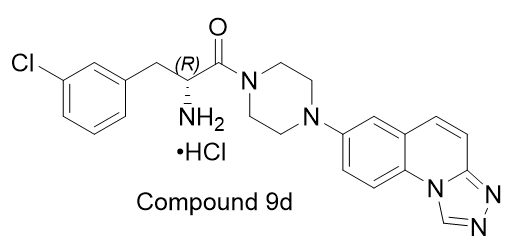
**o**


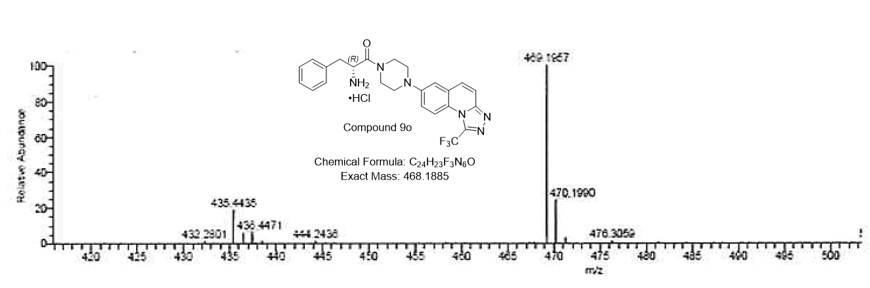


**Figure S60.** The HRMS chromatogram of compound **9o**


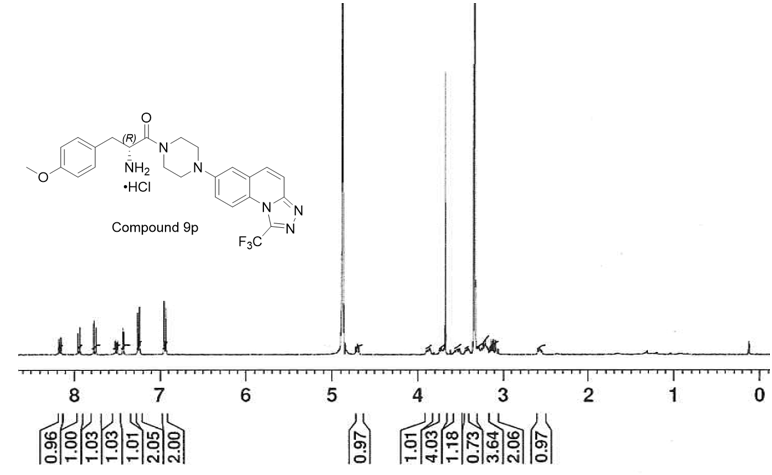


**Figure S61.** The ^1^H NMR spectrum of compound **9p** in **CD3OD**


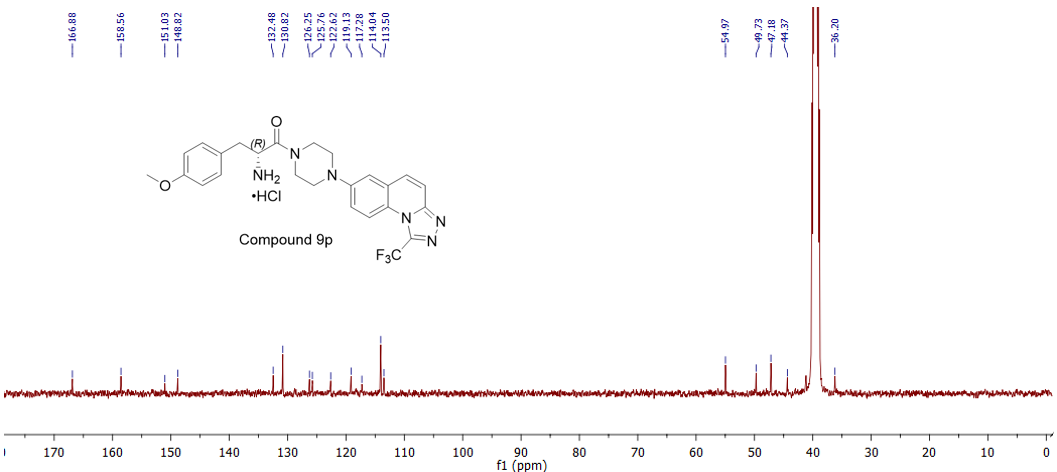


**Figure S62.** The ^13^C NMR spectrum of compound **9p** in **DMSO-d**_6_


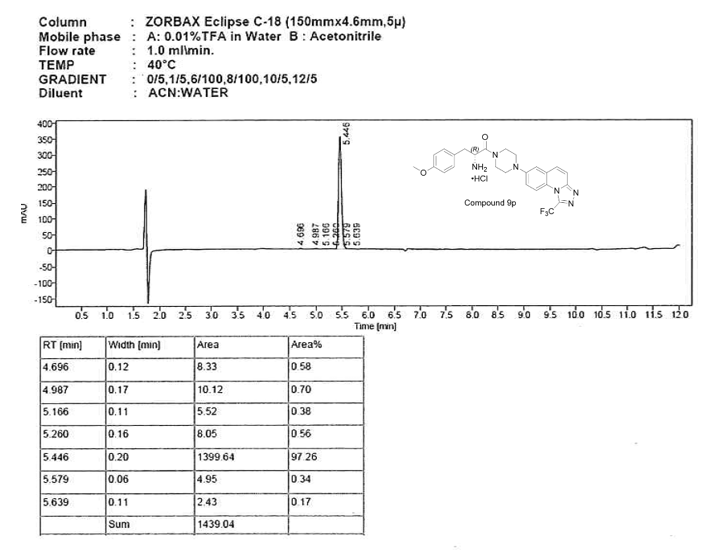


**Figure S63.** The HPLC chromatogram of compound **9**
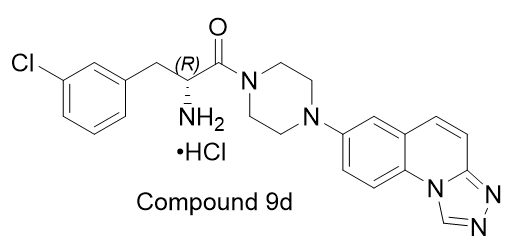
**p**


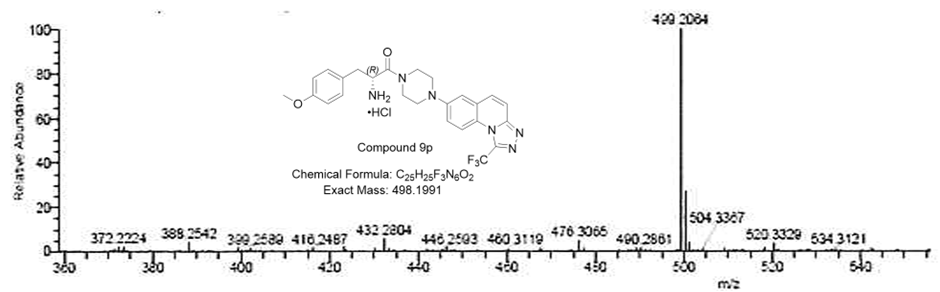


**Figure S64.** The HRMS chromatogram of compound **9p**


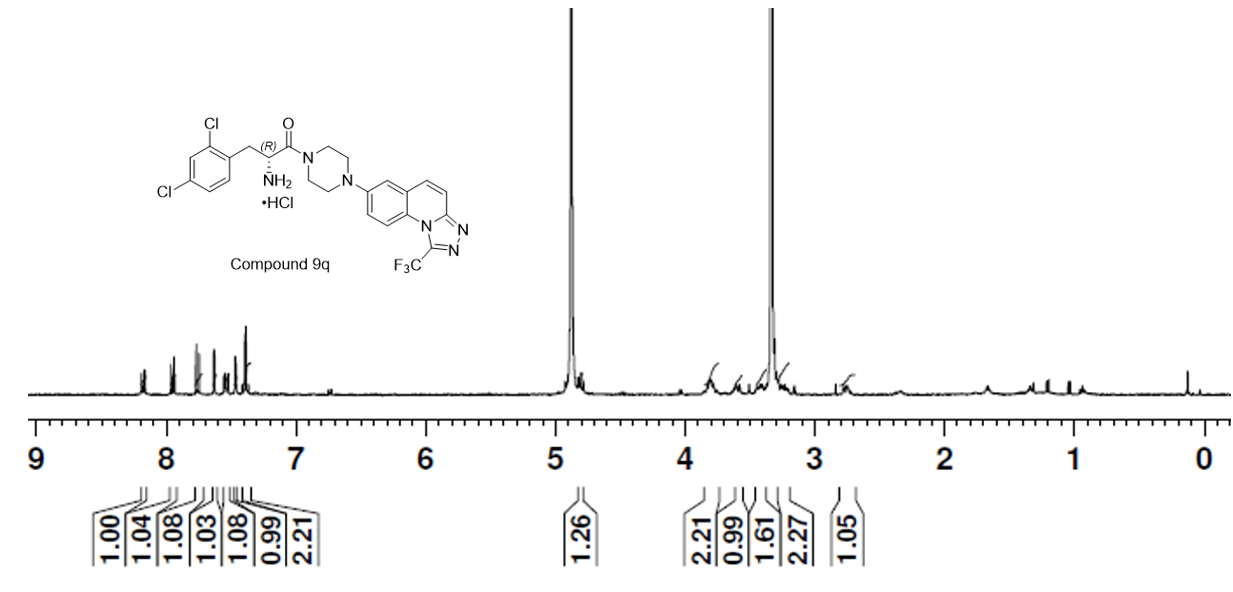


**Figure S65.** The ^1^H NMR spectrum of compound **9q** in **CD3OD**


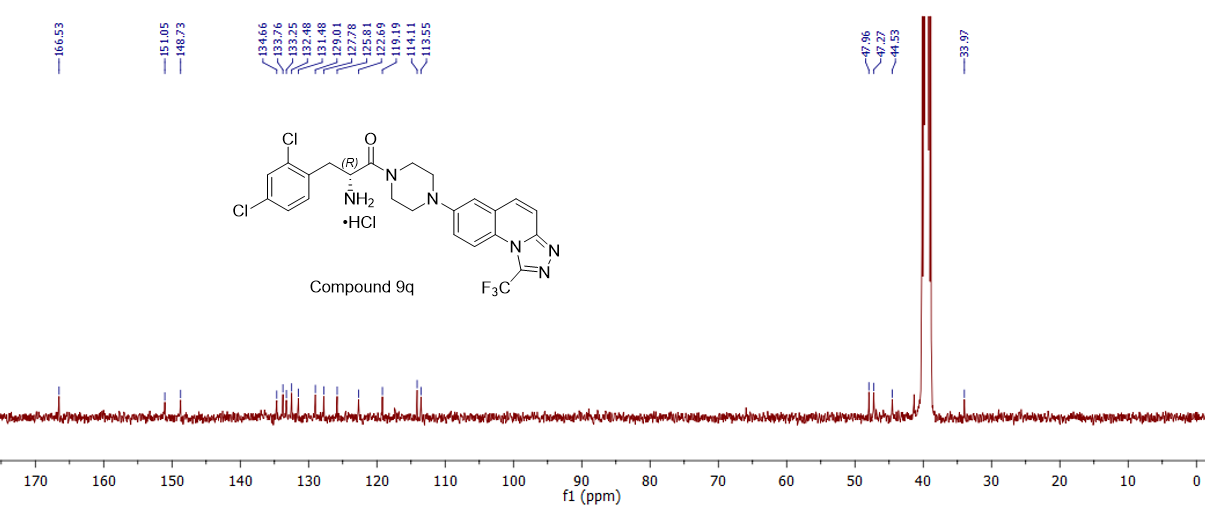


**Figure S66.** The ^13^C NMR spectrum of compound **9q** in **DMSO-d**_6_


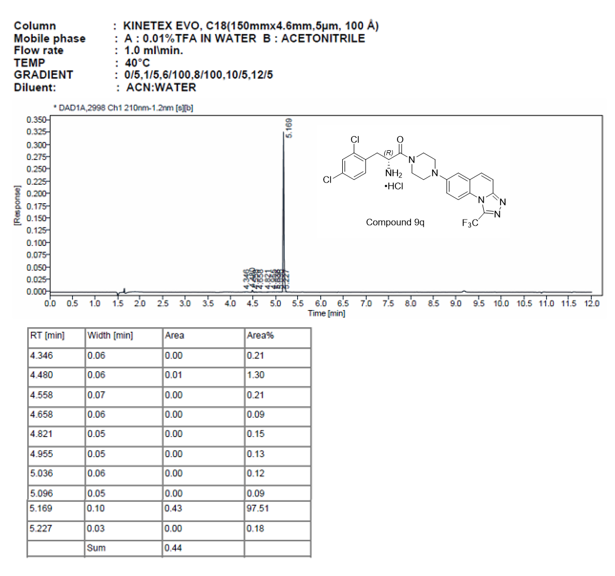


**Figure S67.** The HPLC chromatogram of compound **9**
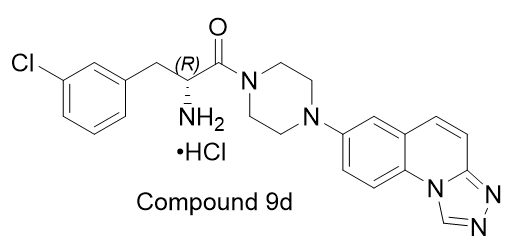
**q**


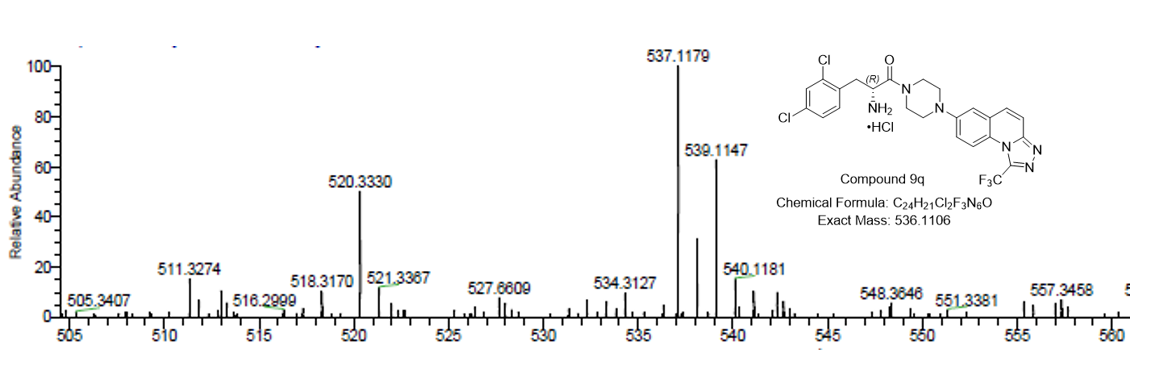


**Figure S68.** The HRMS chromatogram of compound **9**
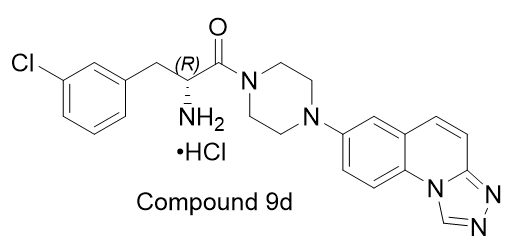
**q**


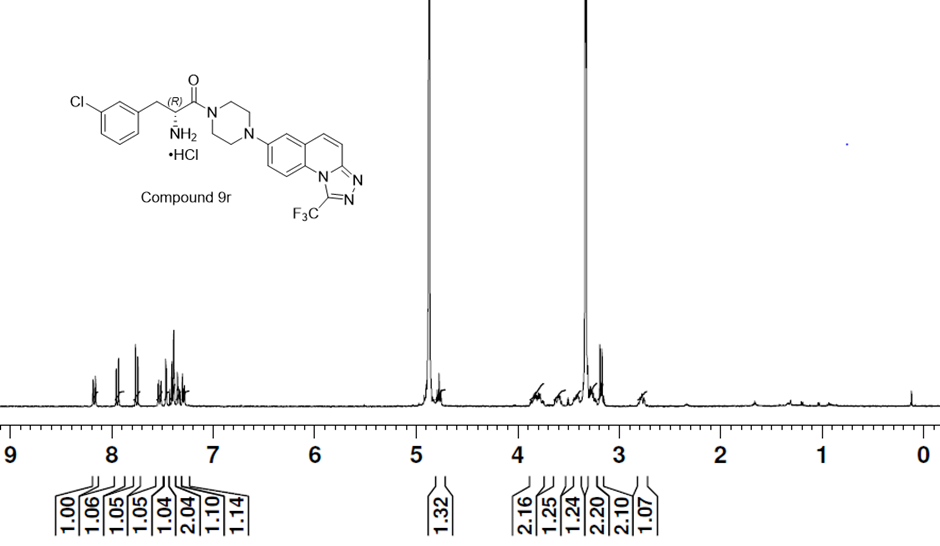


**Figure S69.** The ^1^H NMR spectrum of compound **9r** in **CD3OD**


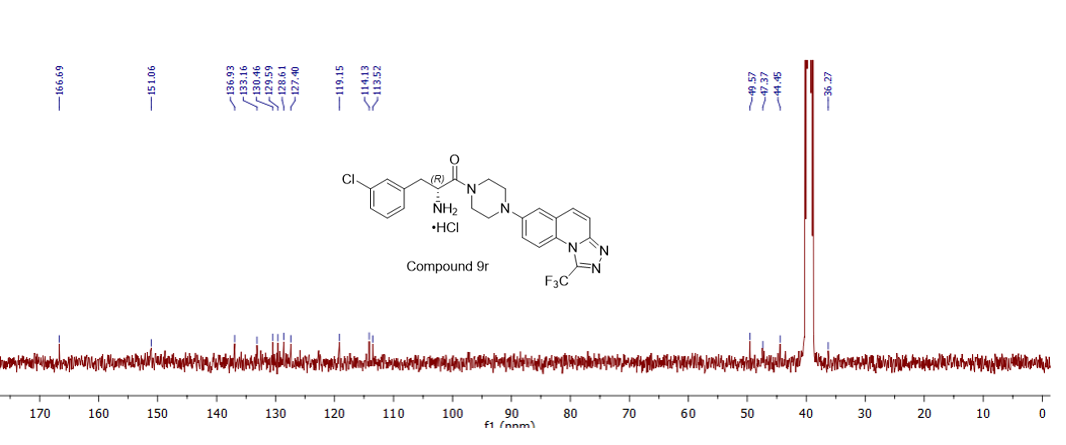


**Figure S70.** The ^13^C NMR spectrum of compound **9r** in **DMSO-d**_6_


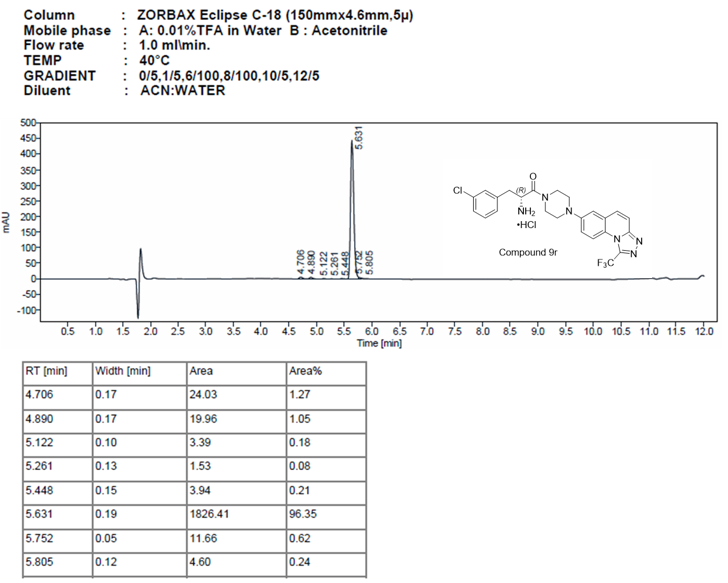


**Figure S71.** The HPLC chromatogram of compound **9**
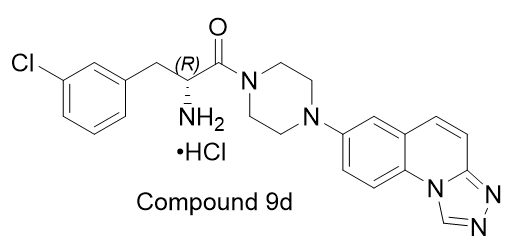
**r**


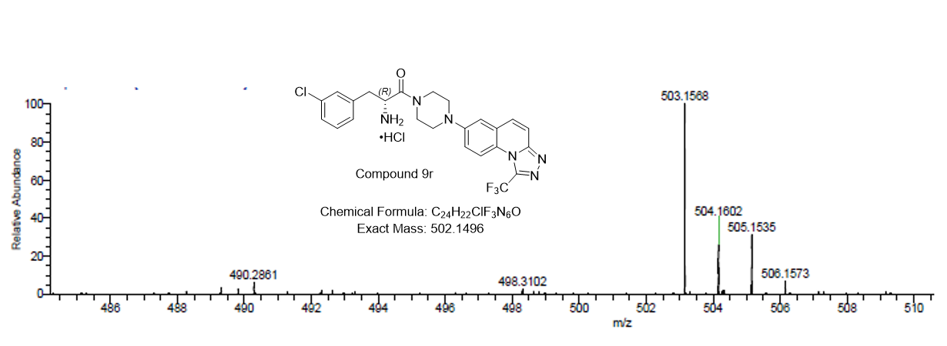


**Figure S72.** The HRMS chromatogram of compound **9**
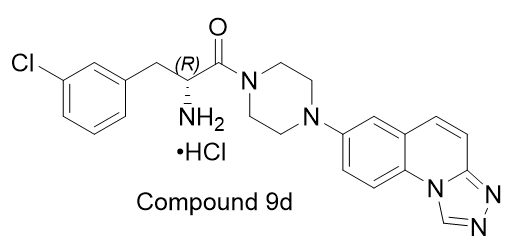
**r**


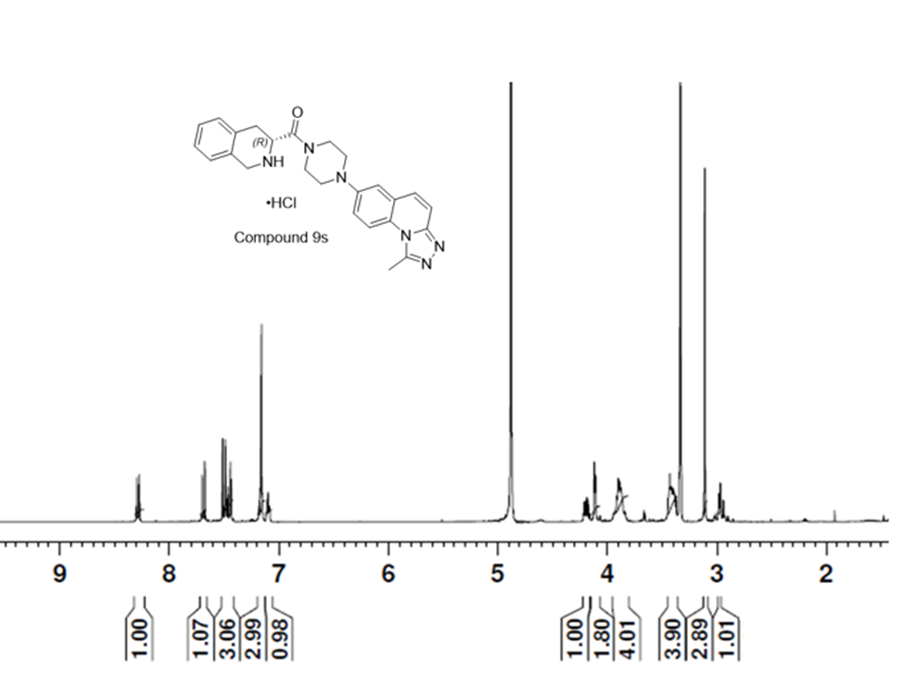


**Figure S73.** The ^1^H NMR spectrum of compound **9s** in **CD3OD**


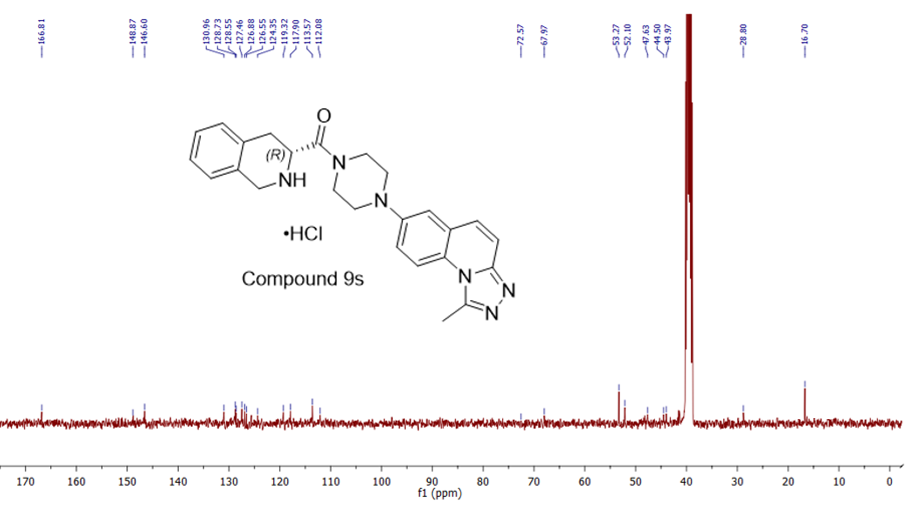


**Figure S74.** The ^13^C NMR spectrum of compound **9s** in **DMSO-d**_6_


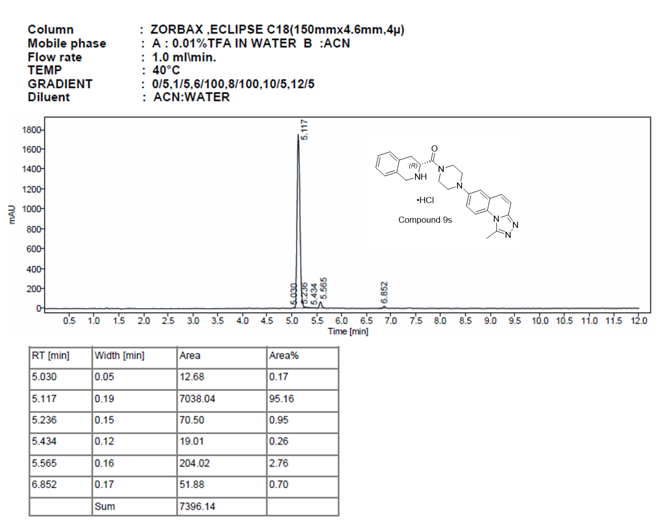


**Figure S75.** The HPLC chromatogram of compound **9**
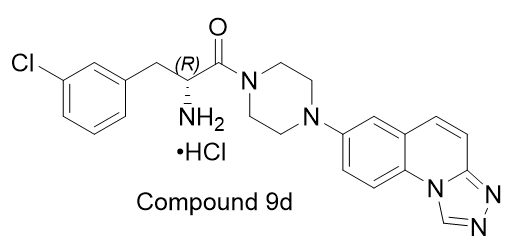
**s**


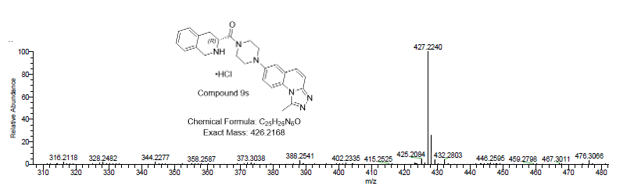


**Figure S76.** The HRMS chromatogram of compound **9**
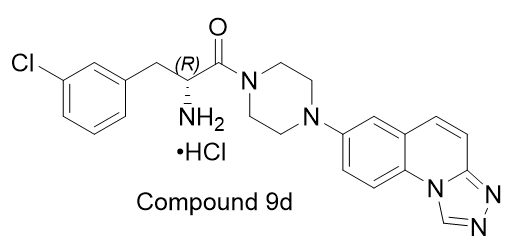
**s**


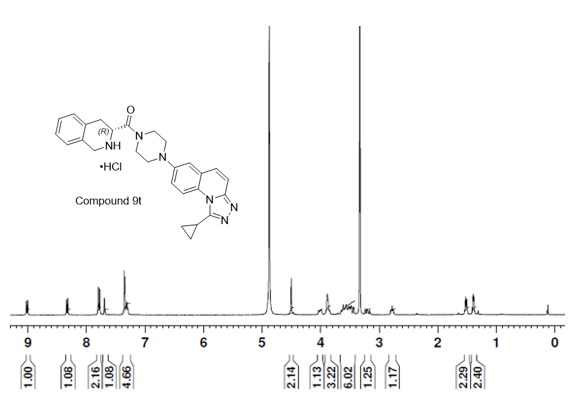


**Figure S77.** The ^1^H NMR spectrum of compound **9t** in **CD3OD**


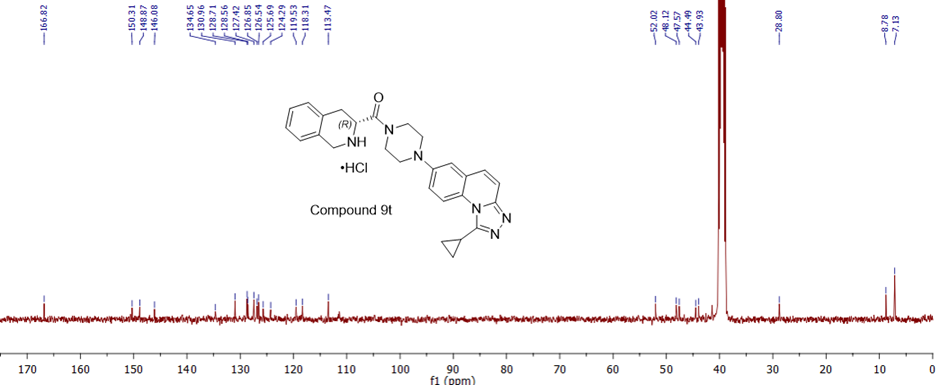


**Figure S78.** The ^13^C NMR spectrum of compound **9t** in **DMSO-d**_6_


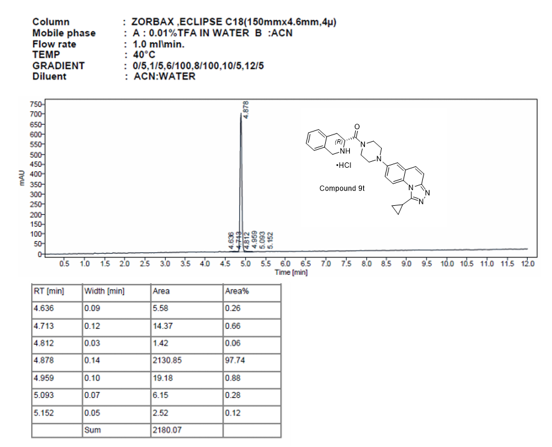


**Figure S79.** The HPLC chromatogram of compound **9**
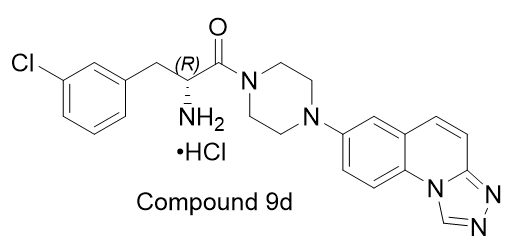
**t**


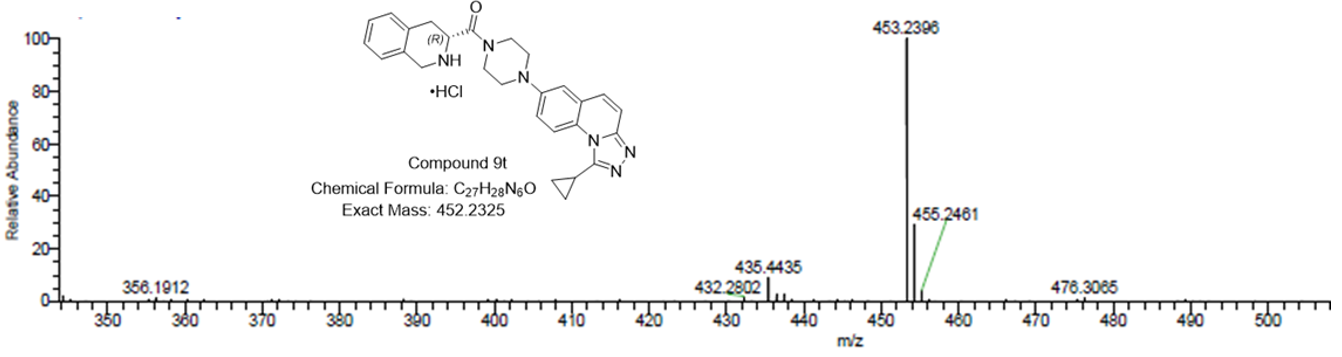


**Figure S80.** The HRMS chromatogram of compound **9**
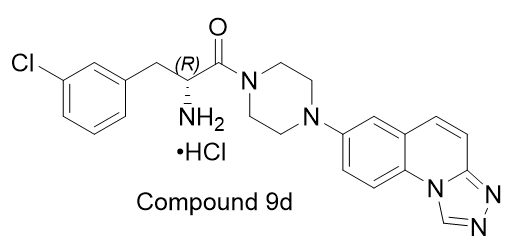
**t**


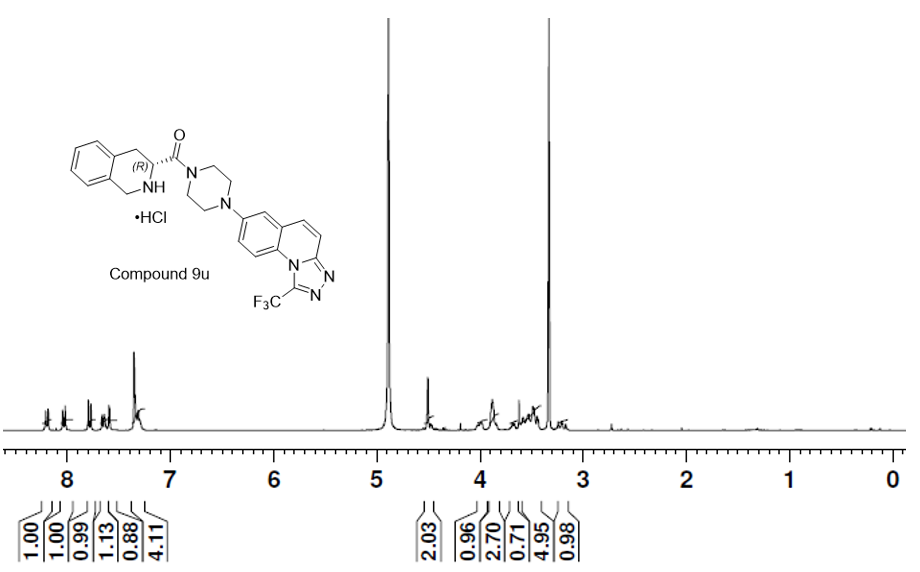


**Figure S81.** The ^1^H NMR spectrum of compound **9u** in **CD3OD**


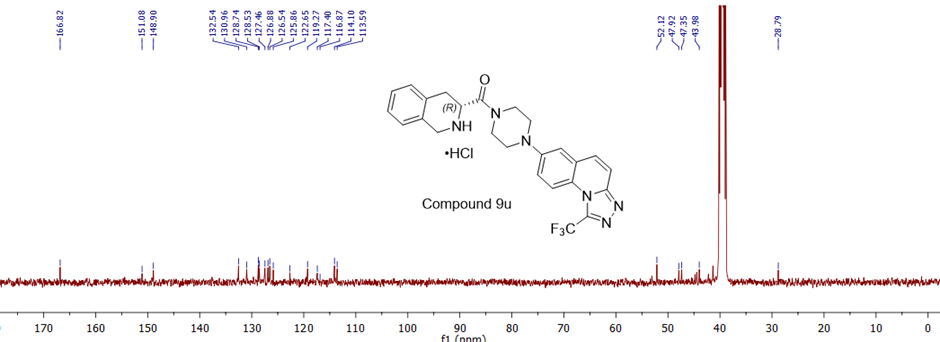


**Figure S82.** The ^13^C NMR spectrum of compound **9u** in **DMSO-d**_6_

**Figure S83.** The HPLC chromatogram of compound **9****u**

**Figure S84.** The HRMS chromatogram of compound **9****u**

1. **IC_50_ graphs**

**Figure S85.** Isoenzyme selectivity of most potent inhibitors (**9d, 9h, 9m & 9r**) against HDAC8.

**Figure S86.** IC_50_ graphs of tested compounds against HDAC8.

1. **Original blots of 9h and 9m**

**Fig. 87 A. Full blot of acetyl SMC3 (Compound 9h) used in fig.9A. The blot shows three biological replicates. The blot used in fig.9A is marked with red square.**

**Fig. 87 B. Full blot of acetyl SMC3 actin (Compound 9h) used in fig.9A. The blot shows three biological replicates. The blot used in fig.9A is marked with red square.**

**.**

**Fig. 87 C. Full length ladder of acetyl smc3 and actin ladder (Compound 9h).**

**Fig. 87 D. Full blot of total SMC3 (Compound 9h) used in fig.9A. The blot shows three biological replicates. The blot used in fig.9A is marked with red square.**

**Fig. 87 E. Full length ladder of total SMC3 (Compound 9h)**

**Fig. 88 A. Full blot of acetyl SMC3 (Compound 9m) used in fig.9B. The blot shows three biological replicates. The blot used in fig.9B is marked with red square.**

**Fig. 88 B. Full blot of acetyl SMC3 actin (Compound 9m) used in fig.9B. The blot shows three biological replicates. The blot used in fig.9B is marked with red square.**

**Fig. 88 C. Full length acetyl SMC3 and actin ladder (Compound 9m)**

**Fig. 88 D. Full blot of total SMC3 (Compound 9m) used in fig.9B. The blot shows three biological replicates. The blot used in fig.9B is marked with red square.**

**Fig. 88 E. Full length total SMC3 ladder (Compound 9m).**
